# Self-organization and time-stability of social hierarchies

**DOI:** 10.1371/journal.pone.0211403

**Published:** 2019-01-29

**Authors:** Joseph Hickey, Jörn Davidsen

**Affiliations:** Complexity Science Group, Department of Physics and Astronomy, University of Calgary, Calgary, Alberta, Canada; Rutgers The State University of New Jersey, UNITED STATES

## Abstract

The formation and stability of social hierarchies is a question of general relevance. Here, we propose a simple generalized theoretical model for establishing social hierarchy via pair-wise interactions between individuals and investigate its stability. In each interaction or fight, the probability of “winning” depends solely on the relative societal status of the participants, and the winner has a gain of status whereas there is an equal loss to the loser. The interactions are characterized by two parameters. The first parameter represents how much can be lost, and the second parameter represents the degree to which even a small difference of status can guarantee a win for the higher-status individual. Depending on the parameters, the resulting status distributions reach either a continuous unimodal form or lead to a totalitarian end state with one high-status individual and all other individuals having status approaching zero. However, we find that in the latter case long-lived intermediary distributions often exist, which can give the illusion of a stable society. As we show, our model allows us to make predictions consistent with animal interaction data and their evolution over a number of years. Moreover, by implementing a simple, but realistic rule that restricts interactions to sufficiently similar-status individuals, the stable or long-lived distributions acquire high-status structure corresponding to a distinct high-status class. Using household income as a proxy for societal status in human societies, we find agreement over their entire range from the low-to-middle-status parts to the characteristic high-status “tail”. We discuss how the model provides a conceptual framework for understanding the origin of social hierarchy and the factors which lead to the preservation or deterioration of the societal structure.

## 1 Introduction

Animals, including humans, form social hierarchies [1–5]. How these hierarchies form and what makes them remain stable over time is a central question across many different fields. In the humanities, social and political theorists have studied the origin of class structures and the conditions under which these structures are preserved or change [6–9]. Archaeologists and other researchers from diverse fields study the factors that lead to the collapse of civilizations [10, 11]. Anthropological research has focused on the roles of norms, sanctions, and cooperative behaviour in creating and maintaining hierarchy [12–14]. In the biological sciences, researchers have questioned whether hierarchy emerges primarily from differences in intrinsic qualities of individuals (e.g. physical strength, intelligence, or aggressive tendency) or as a self-organizing process in which a hierarchy arises as a result of many interactions between the members of the society [15–18].

From a high-level perspective, a fundamental question arises: Can a stable or long-lived hierarchical structure occur entirely by self-organization, based solely on inter-individual interactions, modeled as independent pair-wise “fights”? And, if so, what are the typical structures of hierarchy, and what are the characteristic times of formation and evolution of the said structures?

“Winner-loser” models are a class of mathematical models that have been used to study the self-organization of social hierarchy in biology [18–29] and economics [30–34]. In these models, individuals are characterized by a property, such as “strength”, “resource holding potential”, or “wealth”, that determines the individual’s position in society (in the following, we use “strength” as a generic term for this property). Pairs of individuals come into contact and engage in an interaction (or “fight”). The fight has a winner and a loser, where the winner experiences a gain in strength, and the loser loses strength. The models have two basic rules: one that determines who wins in a given fight, and another that determines the amount of strength gained or lost in a fight. The distribution of strength, which changes as individuals interact with each other, represents the societal structure resulting from the model. While stable societal structures have been analyzed in previous studies of winner-loser models, the time evolution and intermediary, potentially long-lived societal structures have been mostly neglected. Here, we aim to close this crucial knowledge gap.

To do so, we construct a generalized winner-loser model in which we intend the strength property to represent societal status. The amount of status gained by the winner and lost by the loser of each fight is proportional to the pre-fight status of the losing individual. We define a probability for winning that is determined by the relative statuses of the two competitors, modulated by a parameter spanning a continuous range of degree of authoritarianism from redistributive (lower-status opponent always wins) to totalitarian (higher-status opponent always wins). The latter modulation for winning contains previous models as special cases at specific values of the authoritarianism parameter, and allows a more general description of the dynamics. Over a large range of parameters and excluding these special cases, we find the emergence of long-lived intermediary societal structures (distributions of societal status) for the first time. Establishing the existence of these long-lived structures—which can give the illusion of a stable society—and the relationship between the characteristic time of their evolution and the model parameters is one of the main contributions of our study.

To demonstrate the relevance of our generalized model and the long-lived structures, we analyze real-world data. Specifically, we compare data from observational studies on wins and losses in animal interactions with the results from simulations of our model, and we compare the distributions of societal status produced by the model with real-world social hierarchies. To make the latter comparison, we use proxies for societal status in large social groups. In both cases, the real-world data are consistent with our model. Specifically, in our model, long-lived intermediary societal structures (distributions of societal status) arise independent of whether any pair of individuals are equally likely to interact or not. In the latter case, however, status distributions with more complex shapes consistent with the household income proxy emerge. We are able to fit the simulated status distributions to USA household income data with good agreement. To our knowledge, this is the first model that produces the two-part structure of the proxy distribution by self-organization based solely on interacting individuals.

The model is presented in section 2 and an extended version of it in which similar-status individuals interact more frequently than individuals with large differences in status is presented in section 2.1. Details about the shapes of the status distributions and their evolution in time are presented in section 3. Comparison of model results to data from real societies is contained in section 4, where we consider data on agonistic interactions in non-human animals in section 4.1 and proxies for societal status in large social groups (social insects and humans) in section 4.2. The article concludes with a summary of results and some comments regarding future research directions.

## 2 Definition of the model

Winner-loser models have been constructed using many variations of the rule determining who wins the fight and the rule determining the amount of strength gained or lost in a fight, where the particular formulation chosen for each rule depends on the system under study.

In the rule determining the amount of strength gained and lost in a fight, two formulations have been applied previously. In one version (“additive” rule), the effect of fighting on an individual’s strength accumulates additively, for example, by the addition or subtraction of a fixed increment of status [20–25]. In an additive rule, the amount of strength gained or lost in a fight does not depend on the current value of either individual’s strength. This means that the amount of strength won or lost in a fight is always the same, regardless of the strength of one’s opponent.

The other version of this model rule is a “multiplicative” one. Here, the amount of strength gained or lost is proportional to the strength of one of the individuals involved in the fight, such that effect of fighting accumulates multiplicatively [27, 31–33]. Defeating a strong opponent produces a large increase in strength, whereas defeating a weak opponent produces a small increase in strength. It is clear from animal behaviour studies that wins against high ranking individuals increase the rank of an individual more than wins against low ranking individuals. In this case, a multiplicative rule is therefore more realistic than an additive rule, in which it is no more advantageous for an individual to defeat a strong rather than a weak rival. Moreover, whether an additive or multiplicative rule is used leads to substantially different distributions of strength [19]. For example, in many models with additive rules, strength becomes distributed such that individuals of adjacent ranks are separated by the same amount of strength. In multiplicative models, on the other hand, highly skewed distributions can result, and such multiplicative processes have been proposed as a common underlying cause of observed inequalities in natural and social systems [35, 36].

Here, we implement a formulation of the multiplicative rule in which the amount of strength won or lost is proportional to the pre-fight status of the losing individual (“loser scheme”). In another formulation that has been used in several econophysics models [32–34, 37–39], the amount of strength won or lost is proportional to the pre-fight status of the weaker individual, regardless of who wins or loses (“poorer scheme”). The loser scheme formulation is more realistic in the context of dominance hierarchies, because upset victories, in which the lower-strength individual in the pair wins, produce large rewards for the winner and large penalties for the loser. For example, in primate dominance hierarchies, only a small number of repeated defeats of a higher-strength individual by the same lower-strength individual are required for their rankings to be reversed [40]. This scenario is captured by the loser scheme but not by the poorer scheme.

With regards to the rule determining which individual wins in a pairwise fight, two primary formulations have been applied: one in which the probability that the stronger individual wins depends on the difference in the strengths of the two individuals [20–23, 26, 33], and one in which this probability depends on a ratio of the strengths of the two individuals [27–29]. We focus on the latter of these two formulations. This choice is related to our choice of the multiplicative rule for the amount of strength won or lost in the fight. In a multiplicative rule, large absolute differences in strength typically exist among individuals of similar rank, at the top-end of the strength distribution. Therefore upsets, in which the lower-strength individual defeats the higher-strength individual, become very unlikely or impossible at the top-end of the distribution of strength when the probability of winning depends on the difference in strengths of the two individuals. When the probability of winning depends on a ratio of the statuses of the two individuals, upsets tend to be more likely, especially between two high-strength individuals separated by a large absolute amount of strength.

Conversely, in a model with an additive rule for the amount of strength won or lost, it may well be appropriate for the probability of winning to depend on the difference in strengths of the two individuals, since the status of an individual is equal to the difference in the number of times the individual has won and lost fights. However, especially in more complex animals, it is unrealistic to assume that the probability of winning is based on a tally of the number of fights won and lost, as this information is unavailable to the individuals involved in the fight. Rather, a more realistic assumption is that a psychological process occurs in which the two individuals make a rough comparison of one another’s relative strengths, where this comparison influences each individual’s probability of winning via characteristics such as confidence, willingness to take risks, and aggressiveness [2, 27]. This assumption is supported by psychological research showing that perceived change of a physical stimulus depends on the relative rather than the absolute change in the stimulus [19, 41].

Our specific model is constructed as follows. We consider a system of *N* individuals, each possessing a strength property, *S*, that determines the individual’s societal position. We intend *S* to represent the societal status of the individual, and accordingly we refer to “status” rather than the generic term “strength” in the remainder of this article. At each step in the simulation, a pair of individuals is randomly selected, and engages in a “fight”. The probability, *p*, that the higher-status individual wins the fight is expressed as a function of its status, *S*_1_, and that of its (lower or equal status) opponent, *S*_2_:

$$\begin{array}{c} p=\frac{1}{1+{\left(S_{2}/S_{1}\right)}^{\alpha }}. \end{array}$$ (1)

**Fig 1:**
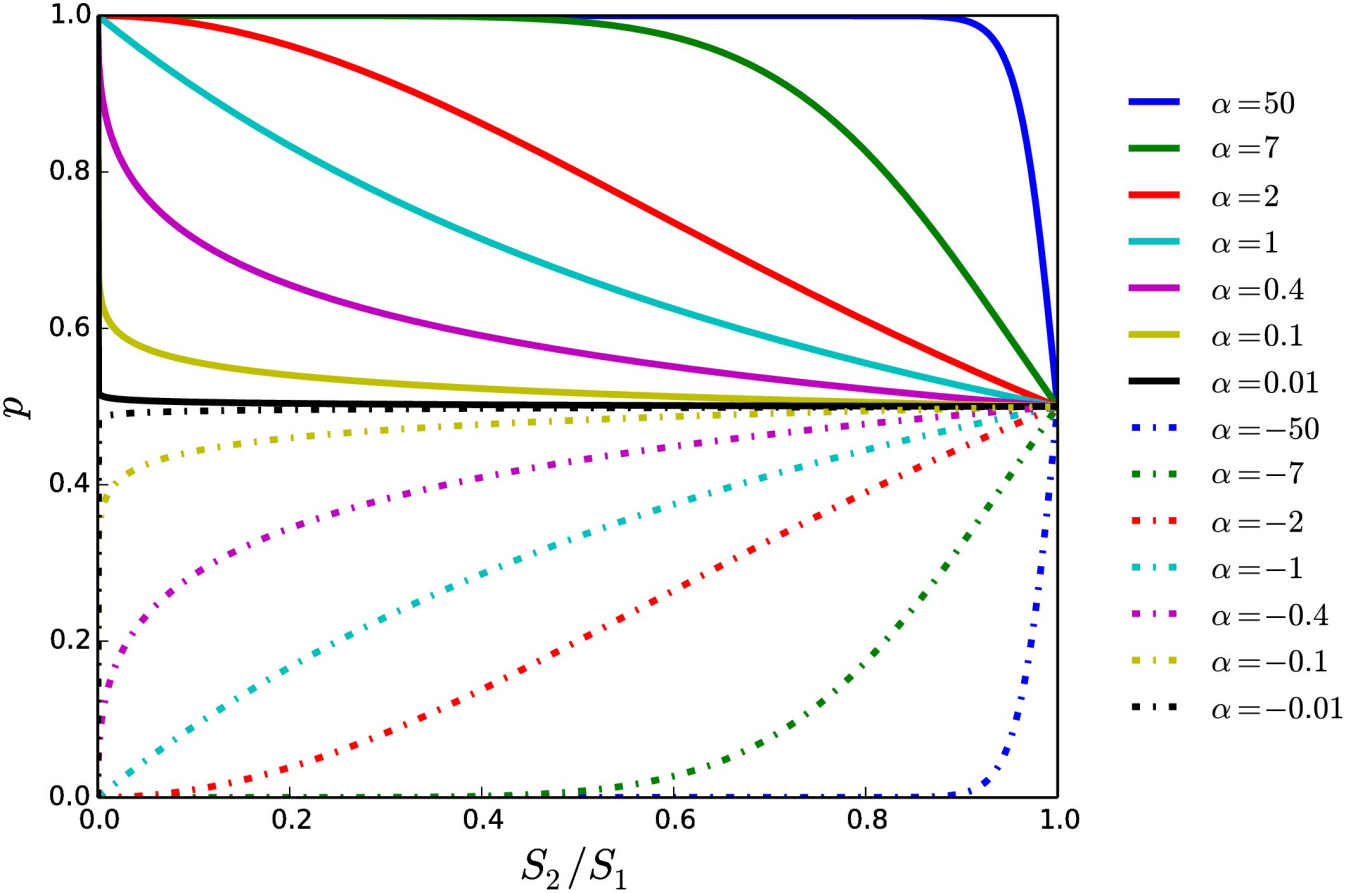
Probability that higher-status individual wins in a pairwise fight. The probability *p*(*S*_2_/*S*_1_) (Eq 1) is shown for different values of *α*. Solid lines correspond to *α* > 0 and dash-dotted lines to *α* < 0.

When *α* = 1, the probability that either individual wins is equal to the ratio of its own status to the sum of its and its opponents statuses. However, as *α* is tuned to values other than 1, the advantage held by the higher-status individual changes (Fig 1). As *α* → ∞, the higher-status individual is virtually guaranteed to win, regardless of how strong its opponent is. On the other hand, when *α* is small but positive, the higher-status individual only has a large advantage in fights against opponents with much lower status. When *α* is negative, 0 ≤ *p* < 0.5, indicating that the lower-status individual in any given fight is more likely to win. The parameter *α* thus generalizes previous modeling approaches, by allowing the probability for winning a pairwise fight to be continuously adjusted between end-points where the lower-status individual always wins (*α* = −∞) and where the higher-status always wins (*α* = ∞).

To interpret the societal meaning of the parameter *α*, we note that the probability *p* depends on the relative statuses of the two individuals. This means that as long as the ratio *S*_2_/*S*_1_ is constant, and given a constant value of *α*, the probability, *p*, that the higher-status individual will win is constant, independent of the absolute values of *S*_1_ and *S*_2_. In a general sense, the probability that a high-status individual will win in a fight against a medium-status individual is the same as the probability that a medium-status individual will win in a fight against a low-status individual. If having a higher societal status can be considered as having a higher level of “authority” in a hierarchical society, then the parameter *α* represents the degree to which there is deference to authority in the society or, in other words, the society’s overall level of “authoritarianism”.

Next, we explain the rule determining the amount of status transferred from loser to winner following each fight interaction. Let *S*_*W*_ be the before-fight status of the winner of the fight, and *S*_*L*_ the before-fight status of the loser. Following the fight, a portion Δ of the loser’s before-fight status is transferred to the winner, such that

$$\begin{array}{c} \begin{array}{c} S_{W}^{\prime }=S_{W}+\Delta \\ S_{L}^{\prime }=S_{L}-\Delta , \end{array} \end{array}$$

where the primed quantities represent after-fight statuses. In our model, the amount of status transferred, Δ, is equal to a proportion of the before-fight status of the individual who loses the fight. That is, Δ = *δS*_*L*_, where *δ* is a fraction between 0 and 1. This gives us:

$$\begin{array}{c} \begin{array}{c} S_{W}^{\prime }=S_{W}+\delta S_{L}\\ S_{L}^{\prime }=S_{L}-\delta S_{L}, \end{array} \end{array}$$ (2)

This rule for the amount of status transferred has realistic implications from the perspective of formation and maintenance of social hierarchy, because it means that upsets (in which the lower-status individual defeats the higher-status individual) produce large rewards for the winner and large penalties for the loser.

The two rules contained in Eqs 1 and 2 constitute our “original” (two-parameter) model that is the main focus of this work. We note that special cases of Eq 1 were investigated in previous work. Specifically, the case when *p* = 0.5 in all fights, regardless of the statuses of the two individuals (*α* = 0 in our model) and the case when *p* = 1 in all fights (*α* = ∞ in our model) were investigated in a model that uses the same rule as our Eq 2 [31]. The special case when *α* = 1 has been used in other winner-loser models, but never in conjunction with a multiplicative rule like our Eq 2. The novel parameter *α* thus allows us to generalize previous models and opens a previously unexplored region of parameter space. We also present a simple extension to our two-parameter model, in the following section.

### 2.1 Model extension: Restricting fights between individuals with large differences in status

One of the main assumptions of winner-loser models based solely on the two categories of rules described in section 2 is that any pair of individuals are equally likely to interact, regardless of their strengths. Some biologically-oriented winner-loser models have included mechanisms that adjust the interaction probability of individuals based on their spatial positions or on their strengths. For example, in Ref. [27], each individual decides whether to engage in a fight by comparing the ratio of its strength to its opponent’s strength with a threshold; in Ref. [29] individuals move in a spatial territory and interact if they are within visual range of one another; and in Ref. [21], individuals interact with a probability equal to the product of a function of their strengths, such that stronger individuals interact more frequently than weaker individuals. In a similar vein, we can extend our model by implementing a third model rule under which pairs of individuals with large differences in status fight less often than similar-status individuals. Unlike other rules that control the probability that two individuals interact, our rule allows all individuals with similar statuses to interact frequently, while also reducing the frequency of interactions (and thus the exchange of status) between individuals with large differences in status.

These are needed realistic features, because evidence from studies of hierarchies in animal groups suggests that a large proportion of the status-determining interactions experienced by high status individuals pit these individuals against “challengers” who themselves have higher than average status [40, 42, 43]. Meanwhile, low status individuals tend not to challenge high status individuals, such that low status individuals are more likely to interact amongst themselves [43]. Similarly, humans are more likely to interact with members of their own social classes, especially at the extremes of the social class spectrum [44], and residential segregation, which impedes interactions between members of different social classes, is considered to be a primary factor in the creation and exacerbation of social stratification [45].

Specifically, our extended model introduces two additional parameters. First, following from observations that high status individuals are more likely to engage in fights with similarly high status challengers, the new rule imposes that two selected individuals only engage in a fight if their absolute statuses are separated by not more than a threshold amount $\eta \bar{S}$. Here, *η* ≥ 0 is a new parameter that sets the size of the threshold relative to the (conserved) average status of the system, $\bar{S}$, which is a natural reference point for the threshold position. Secondly, notwithstanding the above-noted observations regarding the higher frequency of interactions between similar-status individuals, animal behaviour studies also show that high status individuals do interact with low status individuals at times. This occurs, for example, through seemingly random acts of aggression which may play an important role in maintaining hierarchical rank-ordering [46]. For this reason, a realistic model should not exclude the possibility that fights between high and low status individuals will occasionally occur. Therefore, regardless of the result of the threshold criterion, the fight between the two selected individuals takes place if *r* < *ϵ*, where 0 ≤ *ϵ* ≤ 1 is a new parameter and *r* is a random number such that 0 ≤ *r* < 1.

In summation, the new rule to limit interactions based on the statuses of the two competitors can be stated as follows: two individuals are selected at random, and they fight if $S_{1}-S_{2}\leq \eta \bar{S}$ OR *r* ≤ *ϵ*. We note that, in the implementation of the simulation, this rule does not change the probability with which any two particular individuals are selected from the population, but only adds a threshold criterion to decide whether or not the fight occurs between the selected pair.

## 3 Time-evolution and structure of status distributions

In the society envisioned in the model, pairs of individuals interact such that they gain or lose societal status in accordance with the rules described in section 2. As interactions take place, and status is exchanged between the members of the society, a distribution of societal status takes shape. Our primary goal in this study is to investigate the structure of these status distributions and how they evolve in time. In this section, we therefore investigate the shapes of the status distributions as functions of the model parameters *δ* and *α* (section 3.2), and then quantify their time evolution in terms of two characteristic times (sections 3.3-3.5). Before presenting these results, we first (section 3.1) establish how time is defined in the model. This introduces the first characteristic time of the system’s evolution, which gives us a basis on which to present the results in the following sections. Please note that in the directly following sections we focus on the original (two-parameter) version of the model first as many features are qualitatively the same as for the extended version of the model. The features specific to the extended model are then discussed in section 3.6.

### 3.1 Definition of time and the characteristic time *τ*_1_

In order to discuss the shapes and time-evolution of the status distributions formed by simulations of the model, we must establish how time is defined. For simplicity and without loss of generality, we model the pairwise interactions as instantaneous such that they can be described as sequential events. We consider that one unit of time has passed once all members of the society have, on average, engaged in one pairwise interaction or fight. Under this definition of time, the rate at which an individual participates in a fight is an intrinsic frequency of the system, independent of system size, *N*, where *N* is the number of individuals in the system. Time, *t*, is therefore defined as *t* = 2*t*′/*N*, where *t*′ is the number of fights that have occurred since the initiation of the simulation, and the factor of 2 comes from having each interaction involve two individuals. One unit of time is equal to *N*/2 fights.

Previous work by Ispolatov et al. [31] shows the existence of a characteristic time in a model that is mathematically equivalent to our original (two-parameter) model in the case where *α* = 0. An analytic solution for the time evolution of the variance of the status distribution (wealth distribution, in Ref. [31]) was found to be as follows:

$$\begin{array}{c} M_{2}\left(t\right)=\frac{\delta \bar{S}}{1-\delta }(1-e^{-\delta (1-\delta )t}), \end{array}$$ (3)

where the variance (second central moment) *M*_2_(*t*) can be calculated directly from the status distribution:

$$\begin{array}{c} M_{2}\left(t\right)=\frac{\sum_{i=1}^{N}(S_{i}\left(t\right)-\bar{S}{)}^{2}}{N}, \end{array}$$ (4)

where *S*_*i*_(*t*) is the status of individual *i* at time *t*, and $\bar{S}$ is the (conserved) average status.

Similar to Eq 3, higher moments of the status distribution converge to constant values, and the status distribution attains a steady state. Eq 3 therefore shows that the variance approaches a steady-state value of $M_{2}=\delta \bar{S}/\left(1-\delta \right)$ at large times and that the approach to the steady-state is characterized by a time constant equal to (*δ*(1 − *δ*))^−1^. Eq 3 can be re-written in a form that is useful for our purposes:

$$\begin{array}{c} M_{2}=c_{1}\left(1-e^{-t/\tau_{1}}\right), \end{array}$$ (5)

where *c*_1_ and *τ*_1_ are generally functions of *δ* and *α*. In the following, we use the symbols $\hat{c_{1}}$ and $\hat{\tau_{1}}$ to represent these functions when *α* = 0, such that $\hat{\tau_{1}}={\left(\delta \left(1-\delta \right)\right)}^{-1}$ and $\hat{c_{1}}=\delta \bar{S}/\left(1-\delta \right)$, as per Eq 3. Fig A1 in S1 Appendix demonstrates that our definition of time corresponds to how time is defined in the analytical result in Eq 3.

### 3.2 Overview of status distributions produced by the model

In this section, we present the shapes of the societal structures (distributions of status) that emerge in simulations of the model. We begin by setting *α* = 0 (*p* = 0.5 for all fights, regardless of the statuses of the competitors as per Eq 1) because, in this limiting case, there always results a stable steady-state status distribution as we show in the following.

**Fig 2:**
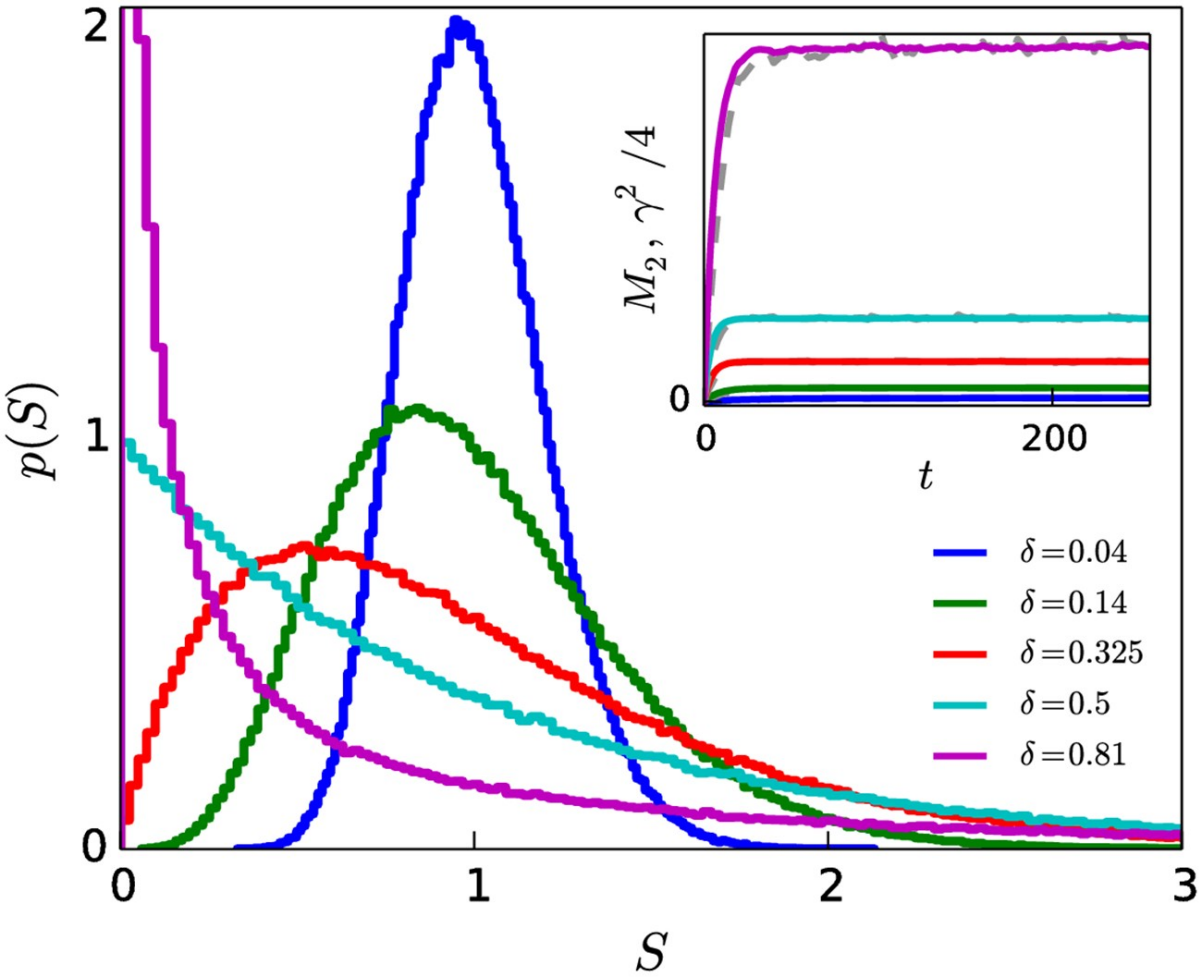
Shape of status distributions as function of *δ*, with *α* = 0. Distributions range from more egalitarian (e.g. *δ* = 0.04: all individuals have close to the average status) to highly unequal (e.g. *δ* = 0.81: most individuals have very low status and small portion of the population has very high status). Inset of (a): plateau in *M*_2_ (coloured) and squared skewness (grey) indicate that status distributions are in steady-state. Larger values of *δ* correspond to larger steady-state value of *M*_2_, such that *M*_2_ provides a measure of the level of inequality of the society. Distributions obtained after simulating up to time $t=64\hat{\tau_{1}}$. *N* = 10^5^, *n*_*r*_ = 5.

In Fig 2, we show graphs of steady-state distributions for several values of *δ* when *α* = 0. As can be seen, the shape of the steady-state distribution varies from rather egalitarian for small *δ* (e.g. *δ* = 0.04: all individuals have close to the average status) to highly unequal (e.g. *δ* = 0.81: most individuals have very low status and small portion of the population has very high status). As was noted in section 3.1, when *α* = 0, our model is mathematically equivalent to the model of Ispolatov et al. [31], who showed that the tail of the distribution decays exponentially for all values of *δ*.

The inset of Fig 2 shows the time evolution of the variance of the status distribution, *M*_2_, which provides a measure of the level of inequality of the society. Larger values of *δ* give rise to larger steady-state value of *M*_2_. As expected from Eq 3, *M*_2_ approaches a steady-state plateau with a value of $\hat{c_{1}}$. The skewness *γ* = *M*_3_/(*M*_2_)^3/2^, where *M*_3_ is the third central moment of the distribution, also arrives at a steady-state plateau at large time (dashed grey lines in inset of Fig 2). This plateau in *M*_2_ and *γ* indicates that the shape of the status distribution is unchanging in time. The plateau in *γ*^2^ is equal to four times that of the plateau in *M*_2_, as can be shown by solving for the third moment following the approach presented in Ref. [31].

**Fig 3:**
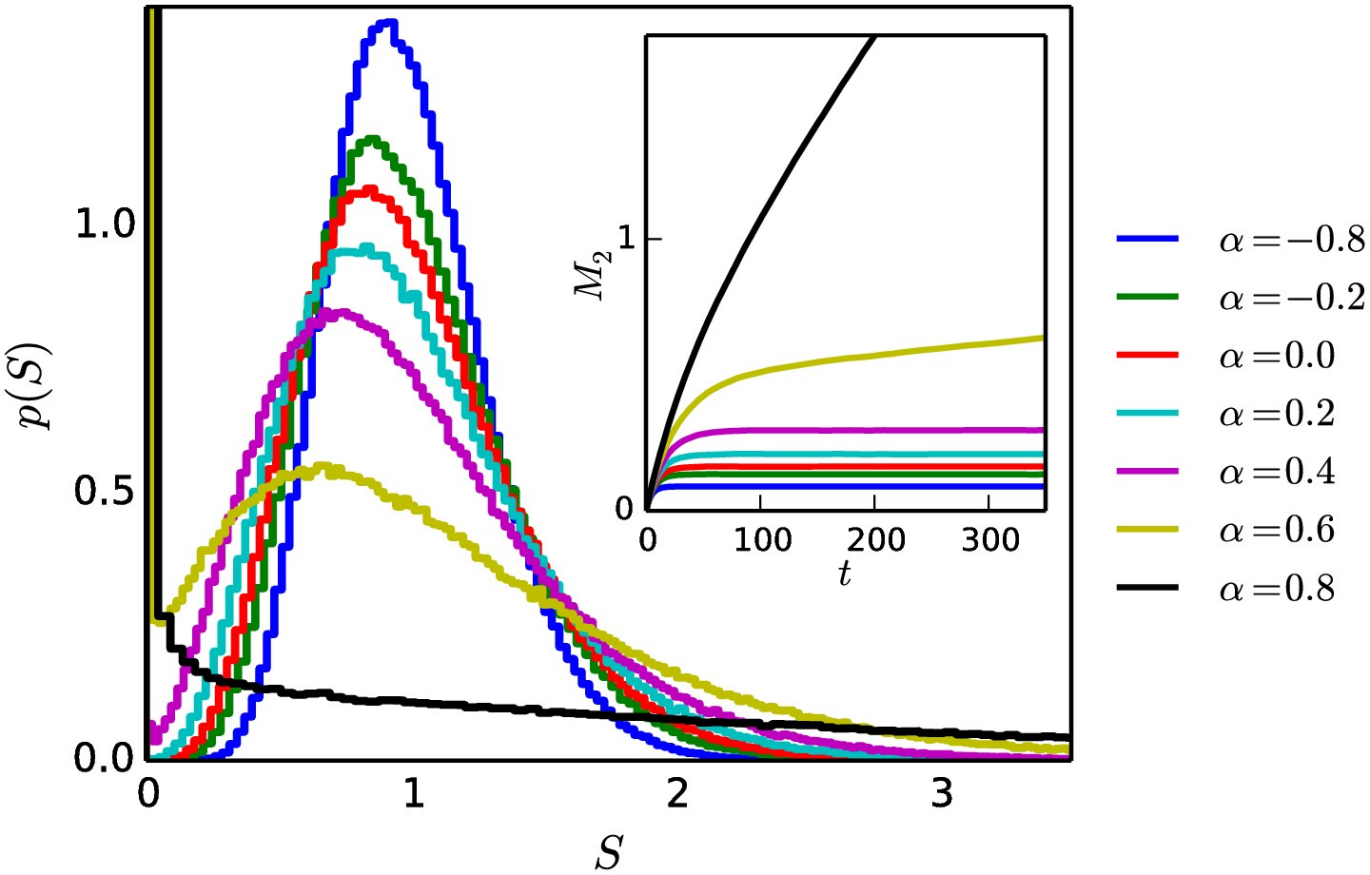
Shape of status distributions as functions of *α*, with *δ* = 0.14. Increasing *α* leads to an increase in the level of inequality of the society, while decreasing *α* leads to a decrease in the level of inequality. Inset: when *α* > 0, *M*_2_ does not attain a plateau but continues to increase with *t*, at a rate that depends on *α*. The status distribution appears to be in steady-state for small values of *α* > 0 (e.g. *α* = 0.2 and *α* = 0.4 curves in the inset) when observed on short enough time scales, while they are in fact not. For larger values of *α* > 0 (e.g. *α* = 0.6 and *α* = 0.8 curves in the inset), the level of inequality noticeably increases on the observation timescale, indicating a runaway of the status distribution toward an end-state of maximum inequality. Distributions obtained after simulating up to time $t=64\hat{\tau_{1}}$. *N* = 10^5^, *n*_*r*_ = 5.

Fig 3 shows distributions of societal status obtained for a fixed value of *δ* and for various values of the authoritarianism *α*. The curve for *α* = 0, *δ* = 0.14 from Fig 2 is reproduced in Fig 3, along with the inset, showing that *M*_2_(*t*) undergoes an initial transient period before arriving at a plateau value for times $t\gg \hat{\tau_{1}}$.

However, for large values of *α* > 0 (*α* = 0.6 and *α* = 0.8), the inset of Fig 3 shows a rapid increase of *M*_2_(*t*) that continues beyond the initial transient period. The status distributions are rapidly evolving (“running away”) toward a totalitarian end-state in which a single individual possesses virtually all of the societal status of the system and all other individuals have status approaching zero. The shape of the status distribution changes rapidly during this evolution, becoming more and more skewed with time. Section E in S1 Appendix contains figures showing how the status distributions evolve over long times, and Section F in S1 Appendix contains a proof that only one individual with non-negligible status remains in the totalitarian end-state.

For smaller values of *α* > 0 (*α* = 0.2 and *α* = 0.4), *M*_2_(*t*) (and therefore the shape of the status distribution) appears to be virtually unchanged in the time following the initial transient period shown in Fig 3. Yet, as we show below (sections 3.3 and 3.4), *M*_2_(*t*) does increase with *t*, albeit much more slowly, such that the status distributions can be considered to be in a long-lived state, where the shape of the distribution can change so slowly that it is essentially unchanged over sufficiently short observation times.

Fig 3 also shows the status distributions that arise when *α* < 0. For this region of parameter-space, the lower-status individual has a higher probability of winning the fight than the higher-status individual. Our numerical simulations show that the distributions are in steady-state (as for *α* = 0) and become more egalitarian (smaller *M*_2_) as *α* is decreased while *δ* is held constant.

The plots in Figs 2 and 3 were obtained using an “egalitarian” initial condition, in which all individuals have an initial status of *S* = 1. Figs B1 and B2 in S1 Appendix show that the same distributions that arise under an egalitarian initial condition also arise when the system is prepared in a “uniform” initial condition in which the initial statuses are randomly selected from a uniform distribution with $\bar{S}=1$.

### 3.3 Long-lived behaviour

**Fig 4:**
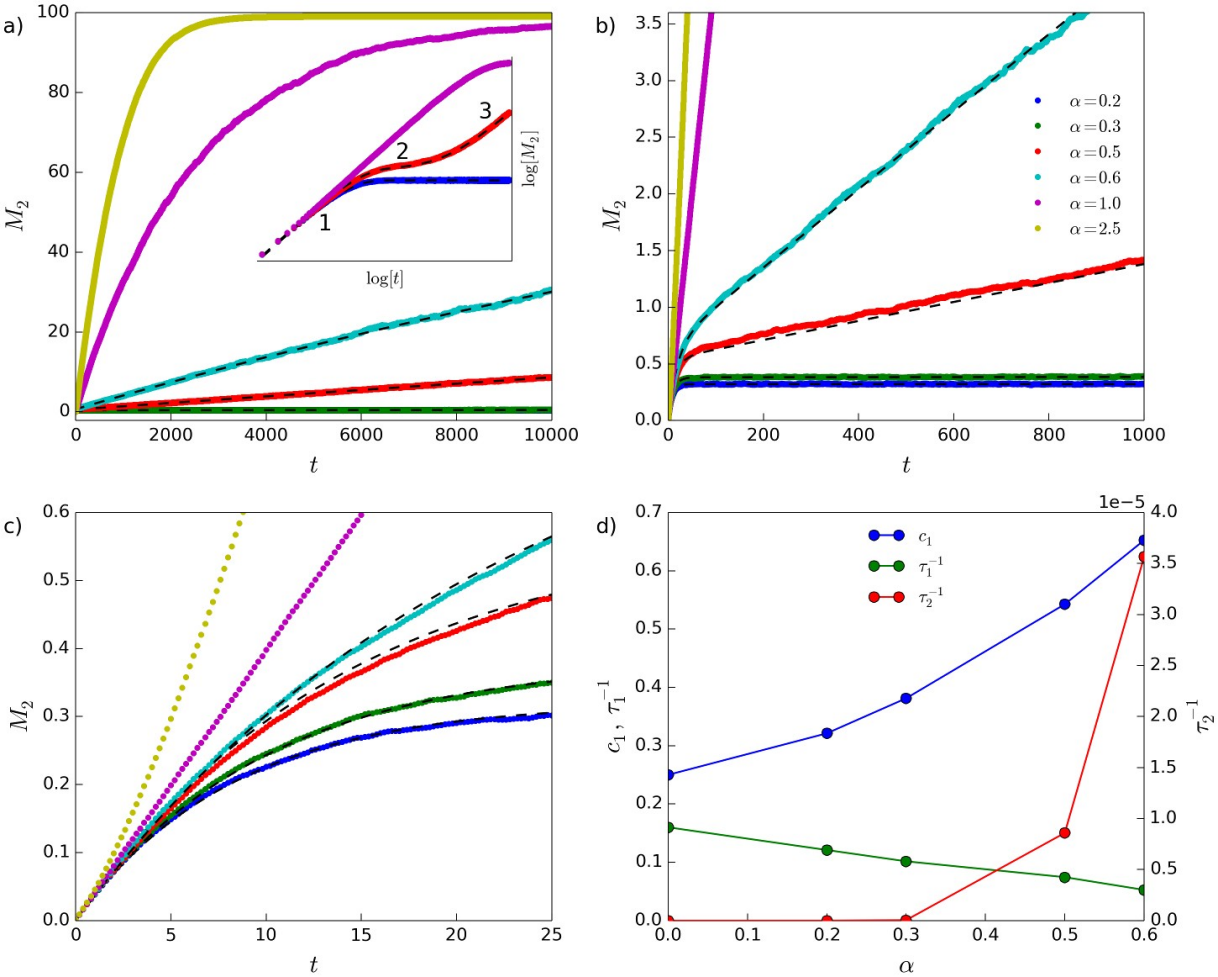
Evolution of *M*_2_(*t*) for *α* > 0. Values of *α* are indicated in the legend in (b), and *δ* = 0.2 for all curves. (a) shows rapid ascent of *M*_2_(*t*) to upper plateau value for *α* = 1.0 and *α* = 2.5 (main plot), and the inset of (a) shows three of the curves on log-log scale, with stages 1-3 of the evolution of *M*_2_(*t*) indicated for the *α* = 0.5 curve. (b) and (c) show the main plot of (a) at different magnifications, to allow inspection of the fit of Eq 7 (dashed black lines) to the curves with *α* ≤ 0.6. (d) fit parameters, with y axis scale for $\tau_{2}^{-1}$ shown on righthand side (parameters for *α* = 0 are from Eq 7, assuming *τ*_2_ = ∞). *N* = 100, *n*_*r*_ = 500.

In this section we quantify the time evolution and the long-lived behaviour of the status distributions produced by the model by examining the evolution of the variance of the status distribution, *M*_2_(*t*), when *α* > 0 (Fig 4a–4c).

As noted in section 3.2, for large values of *α*, the status distribution runs away to an end-state in which a single individual possesses virtually all of the status in the society, and all other individuals have virtually zero status. In this totalitarian end-state, the variance *M*_2_ approaches an upper plateau

$$\begin{array}{c} c_{2}=\left(N-1\right){\bar{S}}^{2}=N-1, \end{array}$$ (6)

where the average status is defined in the model to be $\bar{S}=1$, without loss of generality. The upper plateau *c*_2_ is the maximum possible value of *M*_2_ (indicating the maximum level of inequality of the society). For finite-sized systems, *M*_2_ rises to this upper plateau at large times. This large-time ascent to *c*_2_ happens quickly for large values of *α*, and much more slowly for smaller values of *α* (main plot of Fig 4a).

There therefore appear to be two relevant time-scales in the dynamics of the status distributions: one controlling the evolution away from the initial condition, and a second controlling the long-time approach to the totalitarian end-state. To capture the dynamics of *M*_2_(*t*) for *α* > 0, we attempt to fit, to the simulation data, a sum of exponential functions:

$$\begin{array}{c} M_{2}=c_{1}\left(1-e^{-t/\tau_{1}}\right)+\left(c_{2}-c_{1}\right)\left(1-e^{-t/\tau_{2}}\right), \end{array}$$ (7)

where *τ*_2_ is a characteristic time controlling the rate of approach of *M*_2_ to the upper plateau. The first term in Eq 7 relates to the short-time dynamics of the status distribution, while the second term relates to the long-time dynamics. Long-lived states are produced for values of the model parameters *α* and *δ* for which *τ*_2_ is much larger than the time, *τ*_*obs*_, over which the system is observed (simulated), and *τ*_1_. When *τ*_1_ ≪ *t* ≪ *τ*_2_, Eq 7 becomes $M_{2}\left(t\right)\approx c_{1}\left(1-e^{-t/\tau_{1}}\right)$ where, for *α* > 0, *c*_1_ represents an operational plateau value of *M*_2_(*t*) corresponding to the long-lived state.

Fits of Eq 7 to simulated data are shown by the black dashed lines in Fig 4. For these fits, *c*_2_ is fixed at its upper plateau value *c*_2_ = *N* − 1. Although the fits are imperfect (Fig 4c), they do appear to capture the short-time “elbow” controlled by *τ*_1_ and *c*_1_, and the long-time ascent toward the upper plateau controlled by *τ*_2_ (Fig 4b). Fig 4d shows the fit parameters as functions of *α*: as *α* is increased, both *c*_1_ and *τ*_1_ increase, resulting in a slower evolution of *M*_2_ (and therefore, of the shape of the status distribution) away from the initial condition. On the other hand, as *α* increases, *τ*_2_ decreases, leading to a more rapid (long-time) approach to the upper plateau. As *α* is increased to larger values, it becomes difficult to resolve the early-time “elbow”, and thus difficult to obtain a meaningful fit of Eq 7. Furthermore, as shown in Fig 4c, the shape of *M*_2_(*t*) appears to approach a straight line (at early times) as *α* → 1, and then, to bend upwards away from such an initial straight line when *α* > 1.

For small *α* > 0, the status distributions pass through three phenomenological stages, beginning with an egalitarian initial condition and ending in the totalitarian end-state (see Section E in S1 Appendix for details). The first stage pertains to the evolution of the status distribution away from its initial condition over time scales of the order *τ*_1_ and into a distribution with a form similar to that of the *α* = 0 steady-state distribution. This “stage 2” distribution changes only very slowly, eventually transitioning into a stage (“stage 3”) where high status individuals are nearly guaranteed to win all fights. The duration of stage 2 decreases and essentially disappears as *α* is increased, explaining the inability of Eq 7 to represent *M*_2_(*t*) for larger values of *α*. In the asymptotic state of the evolution *t* ≫ *τ*_2_, all individuals have *S* ≈ 0, except for a single individual with status equal to the total status of the system.

Our findings are largely independent of the system size *N*. As shown explicitly in Section C in S1 Appendix, the parameters controlling the early-time behaviour of *M*_2_ (*c*_1_ and *τ*_1_) remain constant as the system size, *N*, is increased, whereas the parameters controlling the long-time behaviour of *M*_2_ (*c*_2_ and *τ*_2_) both scale linearly with *N*. A proof that the time required to reach the end-state, *τ*_*end*_, scales linearly with *N* in the extreme scenario where *δ* = 1 and *α* = ∞ is also included in Section D in S1 Appendix, as a demonstration of the configurational reasons why the long-time (approach to the end-state) evolution of the model dynamics increases in proportion to the system size *N*.

### 3.4 Phenomenology of the characteristic time *τ*_2_ when *α* > 0

The characteristic time *τ*_2_ controls the rate at which the system approaches the totalitarian end-state when *α* > 0. This characteristic time increases as *α* is decreased from large positive values, as shown in Fig 4d. We can also see, from comparison of Eqs 3 and 7, that *τ*_2_ = ∞ when *α* = 0. We would like to know the functional relationship between *τ*_2_ and the model parameters in order to quantitatively characterize the transition between long-lived and runaway behaviours. To further explore this relationship, we consider an analogy with the barrier-like or “activated” processes typical of many physical systems [47]. We find that the resulting Arrhenius equation provides a good description of the relationship between *τ*_2_ and the model parameters *α* and *δ*. This allows us to determine, as a function of the model parameters, the observation times over which status distributions can be considered long-lived, which we summarize in the following section.

The rate of an activated process is proportional to an exponential term containing an energy barrier scaled by temperature. The exponential term is multiplied by a pre-factor called the attempt frequency, which is typically independent of temperature. This type of relationship between rate and temperature can be found in many diverse physical phenomena, including the rate of chemical reactions [47], the relationship between diffusion coefficients and temperature [47], the rate of nucleation according to the classical nucleation theory [48], the viscosity of strong glass-formers [49], and the blocking transition in superparamagnetism [50], as well as in biology regarding, for example, the rate of chirping in crickets and of flashing in fireflies, and in psychology, where human perception of time is related to body temperature through a relationship of this form [51].

**Fig 5:**
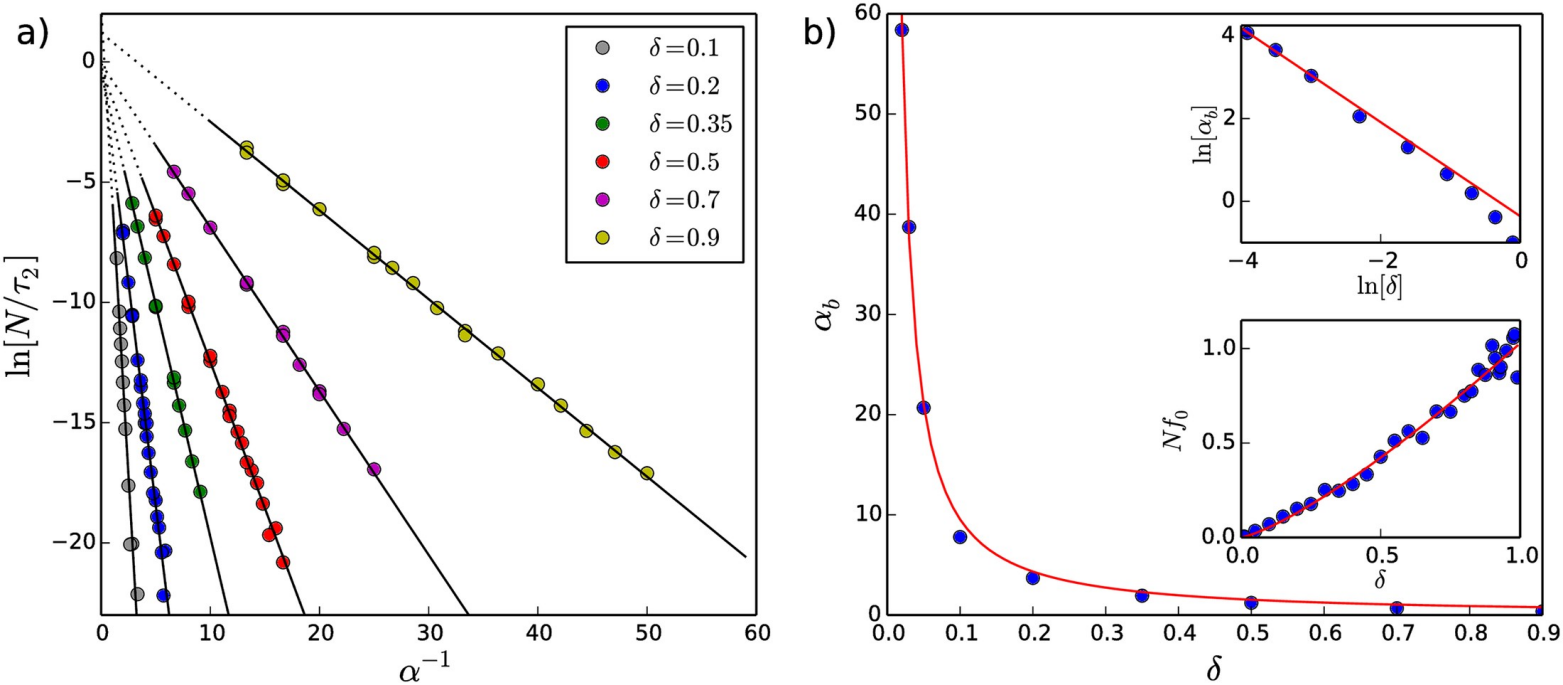
Arrhenius relationship between *τ*_2_ and *α* and *δ*. a) Plots of ln[*N*/*τ*_2_] vs. *α*^−1^ for various values of *δ* confirm the relationship proposed in the Arrhenius equation (Eq 8). The slope of each linear fit is −*α*_*b*_(*δ*). A discussion regarding the evaluation of errors on the extracted values of *τ*_2_ is included in Section G in S1 Appendix. b) Dependence of *α*_*b*_ and *Nf*_0_ on the parameter *δ*: the red line in the main plot (linear scale) and upper inset (logarithmic scale) corresponds to *α*_*b*_ = 0.53*δ*^−1.21^; the red line in the lower inset corresponds to *Nf*_0_ = 1.03*δ*^1.28^.

If the characteristic time *τ*_2_ is regulated by *α* according to an activated process, then one would expect the relationship between *τ*_2_ and *α* to follow an Arrhenius equation of the form:

$$\begin{array}{c} \frac{1}{\tau_{2}}=f_{0}e^{-\alpha_{b}/\alpha }, \end{array}$$ (8)

where *α*_*b*_ is a term that plays a role similar to an energy barrier in an activated process and *f*_0_ is analogous to an attempt frequency. To test this idea, we plot the logarithm of *N*/*τ*_2_ vs. *α*^−1^ in Fig 5a. The factor of *N* is included due to the fact that *τ*_2_ scales linearly with *N*, as discussed in section 3.3 above and as shown in Section C in S1 Appendix.

The linear behaviour seen in Fig 5a confirms the relationship between *τ*_2_ and *α* proposed in the Arrhenius equation (Eq 8). In the figure, the *δ*-dependent slopes of the linear fits correspond to −*α*_*b*_, and the intercepts to ln[*Nf*_0_]. The values of *α*_*b*_ extracted from the linear fits in Fig 5a are shown as a function of *δ* in Fig 5b. As can be seen, *α*_*b*_ diverges as *δ* is decreased. The red line in Fig 5b (main plot and upper inset) shows the function *α*_*b*_ = 0.53*δ*^−1.21^.

In Fig 5a, the y-intercepts of the linear fits appear to cluster around 0, suggesting that the prefactor *Nf*_0_ in the expression for *N*/*τ*_2_ following from the Arrhenius equation is of the order of 1 for the values of *δ* considered. The y-intercepts do not, however, give a robust determination of the prefactor *Nf*_0_. This may be due to a change in functional form of Eq 7 as *α* increases such that *τ*_1_ vanishes. Alternatively, the prefactor *Nf*_0_ can be directly determined by setting *α* = ∞ (equivalent to *p* = 1 in Eq 1) in the simulations and extracting *τ*_2_(*α* = ∞) from *M*_2_(*t*). In so doing, we have assumed that *t* ≫ *τ*_1_ and *c*_2_ ≫ *c*_1_ such that Eq 7 becomes $M_{2}\left(t\right)\approx c_{2}\left(1-e^{-t/\tau_{2}}\right)$. *Nf*_0_ is then equal to *N*/*τ*_2_(*α* = ∞). This approach provides more robust determinations of *Nf*_0_, which are shown in the lower inset of Fig 5b, along with a fit (red line) of the function *Nf*_0_ = 1.03*δ*^1.28^.

Substituting the expressions for *Nf*_0_ and *α*_*b*_ into the Arrhenius equation (Eq 8) gives:

$$\begin{array}{c} \frac{N}{\tau_{2}}\approx 1.03\delta^{1.28}e^{-0.53/(\alpha \delta^{1.21})}. \end{array}$$ (9)

The Arrhenius equation and Eq 9 show that *τ*_2_ diverges exponentially as *α* → 0. This is consistent with Eq 3, which indicates that *τ*_2_ = ∞ when *α* = 0. Moreover, the parameter *δ* appears in both the pre-factor and the argument of the exponential of Eq 9, such that as *δ* → 0, *τ*_2_ → ∞, regardless of the value of *α*. This is consistent with the fact that for *δ* = 0 no status is exchanged during a pairwise interaction and the initial status distribution is trivially stable. These observations strongly suggest that stable status distributions only exist for *α* ≤ 0 as well as *δ* = 0. When *α* > 0, Eq 9 allows us to identify the time scale of the transition between long-lived societal structures and runaway toward the totalitarian end-state as we discuss in more detail below.

### 3.5 *δ* − *α* phase diagram

**Fig 6:**
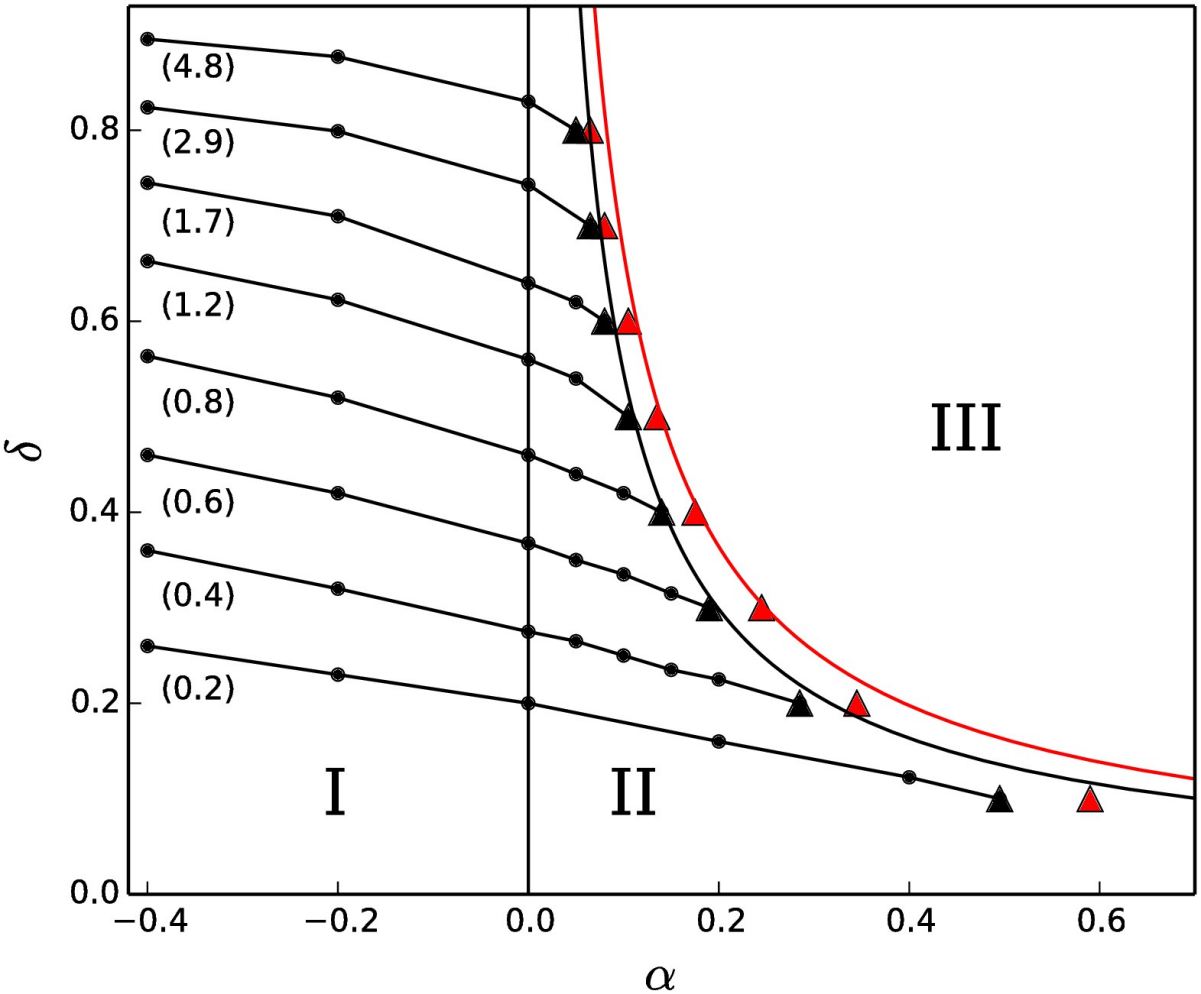
*δ* − *α* phase diagram. The model exhibits three regions of behaviour in *δ* − *α* parameter-space: I (*α* ≤ 0 or *δ* = 0): true (infinite-duration) steady-state status distributions; II (small values of *α* > 0): long-lived status distributions; III (large values of *α* > 0): runaway behaviour. Within regions I and II, equi-*M*_2_ lines (lines of equal standard deviation of the status distribution) are shown with *M*_2_ values indicated in parentheses below each equi-*M*_2_ line. The location of the transition between regions II and III is observation time-dependent, and is marked by the black triangular points for *τ*_*obs*_ = 10^4^ and by the red triangular points for *τ*_*obs*_ = 10^3^, as determined directly from the simulation data. The location of the transition as determined by the Arrhenius relation between *τ*_2_ and *α* is shown by the black (*τ*_*obs*_ = 10^4^) and red (*τ*_*obs*_ = 10^3^) curves. System size *N* = 1000.

As shown in sections 3.2-3.4, for all positive values of *δ*, the model gives rise to three regions of behaviour: true steady-state status distributions (*α* ≤ 0), long-lived status distributions that eventually arrive at the totalitarian end-state (small values of *α* > 0), and runaway (rapid evolution) toward the totalitarian end-state (large values of *α* > 0). These three regions and the transitions between are portrayed in a *δ* − *α* phase diagram in Fig 6.

Fig 6 is a summary of the main results of our model. In it, we see the three regions of behaviour described in sections 3.2-3.4. The region (marked with a roman numeral I) of infinite-duration steady-state status distributions is separated from a region (II) of long-lived status distributions by a transition that occurs due to an exponential divergence of the characteristic time *τ*_2_ as *α* → 0^+^. Runaway behaviour (region III) occurs when a noticeable slope is observed in the evolution of *M*_2_(*t*0 (see the inset of Fig 3 for *α* = 0.6 and *α* = 0.8). Whether such a slope is observed or not depends on *τ*_2_(*δ*, *α*) and on the time, *τ*_*obs*_, over which the system is observed. The location, in *δ* − *α* parameter-space, of the transition between regions II and III therefore depends on *τ*_*obs*_, and can be determined directly from the data or via Eq 9, as described below.

We determined the location of the transition between regions II and III in two ways. First, we used a simple criterion to equate the onset of runaway with the appearance of a positive slope in the long-time portion of *M*_2_(*t*). In this way, the long-lived state corresponds to a plateau value of *M*_2_(*t*) over a particular *τ*_*obs*_ (where *τ*_*obs*_ ≫ *τ*_1_). The long-lived state is considered to be lost when, instead of a plateau, a positive slope is observed in *M*_2_(*t*) after a time *τ*_*obs*_ has transpired. The black and red triangular markers in Fig 6 indicate the onset of runaway as determined by this criterion, for *τ*_*obs*_ = 10, 000 and *τ*_*obs*_ = 1, 000, respectively. Secondly, the location of the transition can be determined using the Arrhenius relation presented in section 3.4 to determine the value of *α* for which *M*_2_(*t*) increases by a sufficient amount after a time *τ*_*obs*_ has transpired. The location of the transition as determined by the Arrhenius relation is shown by the curving curving black and red lines in Fig 6. Details about how these two approaches were conducted are contained in Section I in S1 Appendix.

While *α* largely determines the stability of the asymptotic status distributions, the exact value of *δ* > 0 influences the shape of the status distribution, as already seen for *α* = 0 in section 3.2. This is also true for the long-lived distributions. Essentially identical distributions can be produced (within a particular simulation time) for different combinations of the parameters *δ* and *α* (see Section H in S1 Appendix for details). Sets of such points are plotted as “equi-*M*_2_” lines (lines of equal standard deviation of the status distribution) within regions I and II in Fig 6.

### 3.6 Status distributions in the extended model

In section 2.1, we presented a simple extension to the original (two-parameter) model that restricts which individuals can fight each other based on the proximity in their statuses. This “extended model” introduces two new parameters: *η*, which sets a threshold $\eta \bar{S}$ (where $\bar{S}$ is the average status of the system), such that individuals with statuses separated by an amount more than this threshold are restricted from fighting with each other; and *ϵ*, the probability with which two individuals that have a separation of statuses greater than $\eta \bar{S}$ do nevertheless fight each other.

**Fig 7:**
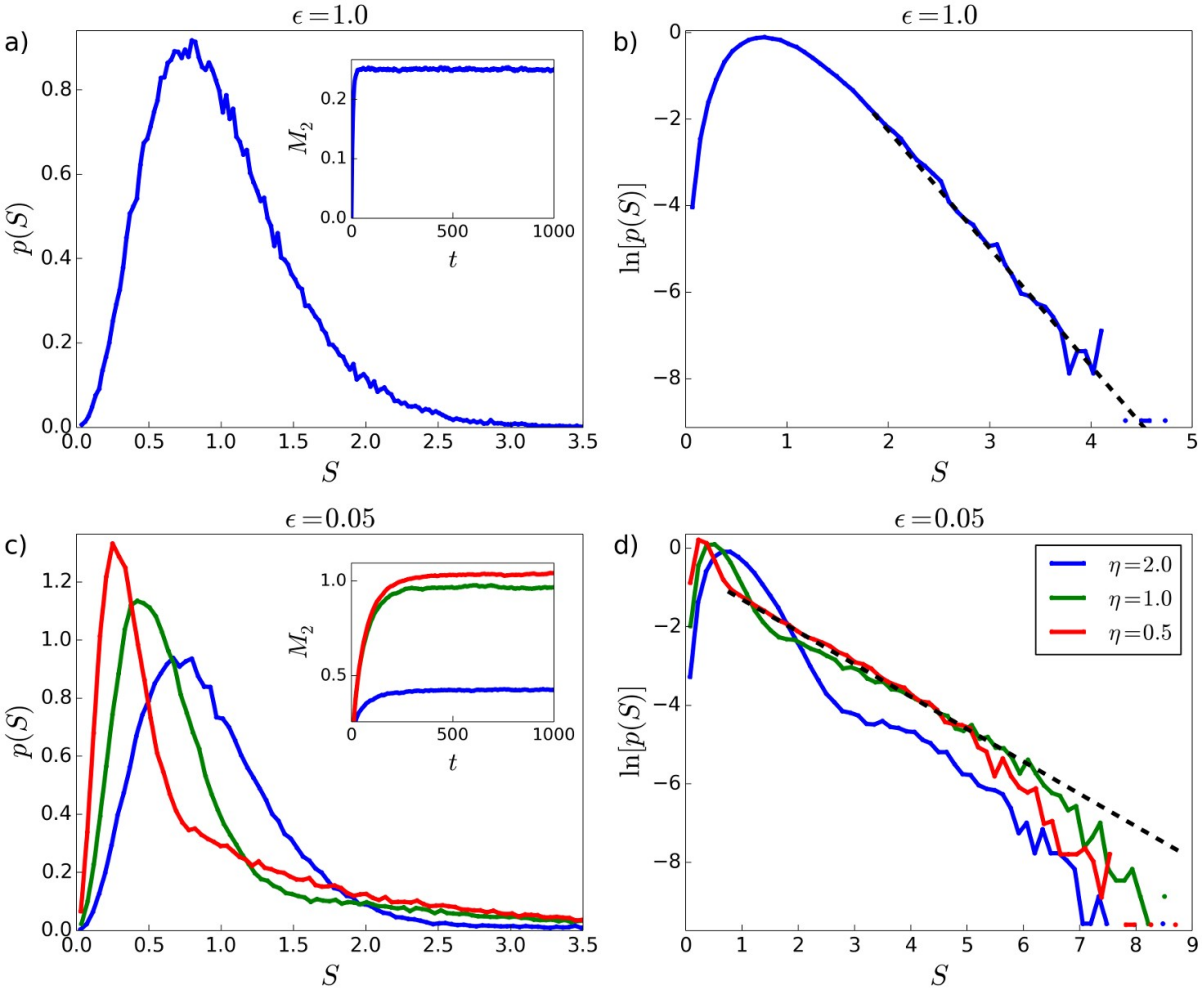
Status distributions in the extended model. *δ* = 0.2 and *α* = 0 in all plots. Plots (b) and (d) show the distributions on a logarithmic scale, in order to allow for inspection of the large-*S* tail. When *ϵ* is decreased from 1, a “break” in the distribution emerges, (particularly evident on the logarithmic scale) corresponding to a society with distinguishable low status and high status groups or classes. The black dashed lines in (b) and (d) are maximum likelihood fits of exponential distributions with lower-bound at *S* = 2.25 in (b) and [lower-bound, upper-bound] at *S* = [0.75, 5.0] in (d), where plotted fit line in (d) is extrapolated beyond *S* = 5.0. System size *N* = 10^5^.

Fig 7 shows distributions of status generated by simulations of the extended model. A log-linear scale is used in the righthand column of plots to allow inspection of the high-status tails of the status distributions. In all subplots, *α* = 0 and *δ* = 0.2. When *ϵ* = 1 (Fig 7a and 7b) the original (two-parameter) model is recovered, such that the parameter *η* has no effect on the shape of the status distribution. Also, when *ϵ* = 1, the high-status tail of the distribution decays exponentially, in accordance with the analytic result found by Ispolatov et al. [31] (Fig 7b). When *ϵ* is decreased, a “break” in the high-status tail of the distribution emerges, as can be seen in the log-linear plot in Fig 7d. Following this break, the distribution enters a second exponentially-decaying regime (black dashed line in Fig 7d) that ends with a cutoff. Increases to *η* cause the location of the break as well as the location of the peak of the distribution to shift to higher values of *S*. The plateaus in *M*_2_(*t*) (insets in Fig 7) for *ϵ* > 0 indicate that these distributions are in steady-state.

When *ϵ* = 0 (not shown), *M*_2_(*t*) does not obtain a plateau and continues to increase over the duration of the simulation time. For this value of the parameter *ϵ*, the system approaches an end-state in which the majority of individuals have status approaching zero, and a small minority of individuals have large statuses. In this end-state, the few high-status individuals are prevented from interacting with each other because their statuses are separated by amounts greater than $\eta \bar{S}$. The specific configuration of the *ϵ* = 0 end-state depends on the particular sequence of interactions. A positive value of *ϵ* is therefore needed in order for the simulation to obtain a unique steady-state.

In the plots in Fig 7, *α* = 0. The distributions produced with *α* = 0, *η* > 0, and *ϵ* > 0 show a plateau in *M*_2_(*t*). When *α* > 0, *M*_2_(*t*) behaves qualitatively in the same way as the original (two-parameter) model. That is, a long-lived state is observed for small values of *α* > 0, and a runaway for large values of *α* > 0, where the location of the transition between the long-lived state and runaway depends on the observation (simulation) time. Figs J1 to J4 in S1 Appendix show how the extended model distributions evolve for representative values of *α* > 0.

## 4 Comparison of model results with real-world data

In this section, we compare the results of our model to data from real-world social hierarchies in two ways. First, we consider data on agonistic interactions (fights) from animal observation studies, in section 4.1. We are able to make some general comments about the parameter values and the stage in the time evolution of the system for which the win and loss patterns in the simulations resemble and are consistent with the animal behaviour data. We note that the available data in this case is for small system sizes. Thus, the history of each particular pair of individuals’ past interactions might be important since the individuals do recognize and remember one another. This feature is not captured by our model, which is a better description for large groups of individuals, in which there is a large probability that an individual *i*’s next interaction will be with an opponent with whom *i* did not interact recently.

Unfortunately, there are currently no observational interaction data for sufficiently large groups of individuals. In order to compare the distributions of societal status from the model with real-world social hierarchies, we therefore seek a measurable quantity that serves as a proxy for societal status in large social groups. Such a proxy must allow the assignment of a status value to all individuals in a large society. We have reviewed potential proxies for status in non-human animals, and found that body-size in insects seems to be the only such quantity for which data is currently available for large groups. We present a comparison to our model in section 4.2.1. In the case of humans, socioeconomic data about large groups is available and we justify the use of household income as a proxy for societal status in large human societies and compare this proxy to status distributions from our model in section 4.2.2.

### 4.1 Agonistic interactions in small groups of animals

In many studies across a wide range of taxa [52], researchers of animal social behaviour have observed and recorded agonistic interactions between pairs of individuals. These interactions, which include aggressions, physical and non-physical threats, and submissive behaviours [1] can be considered as “fights” in which a winner and loser can be identified. From these observations, an “interaction matrix” can be created, with a row and column for each individual, and where each entry (*i*, *j*) of the matrix represents the number of times *i* has defeated *j* in a pairwise fight. Various methods exist to determine a rank-ordering of the individuals in the society from the data contained in the interaction matrix [26, 53–55]. Correlations can then be investigated between hierarchical rank and outcomes such as health, reproductive success, quality of social relationships, and preferential access to resources of the individuals in the social group.

A common observation in many such studies is that the higher-ranked individual in a pair wins the large majority of the fights against the lower-ranked individual. However, in most datasets, a small number of interactions occur in which the lower-ranked individual wins the fight (called “reversals” in the animal behaviour literature). In our model, the distributions of status that most closely resemble this scenario are highly-skewed distributions in which *p* ≈ 1 (see Eq 1) for many pairs of individuals, such that reversals are rare but still possible. This excludes *α* ≤ 0, since reversals are frequent in this range of parameter space, and highlights the relevance of the long-lived states observed in our model (see Fig 6). For small values of *α* > 0, the said distributions occur at late stages in the evolution of the system, and for larger values of *α* > 0, at the said distributions occur at the stage immediately following the evolution of the system away from the initial condition (see the discussion regarding the three phenomenological stages of the evolution of the system described in section 3.3 above and in Section E in S1 Appendix).

**Fig 8:**
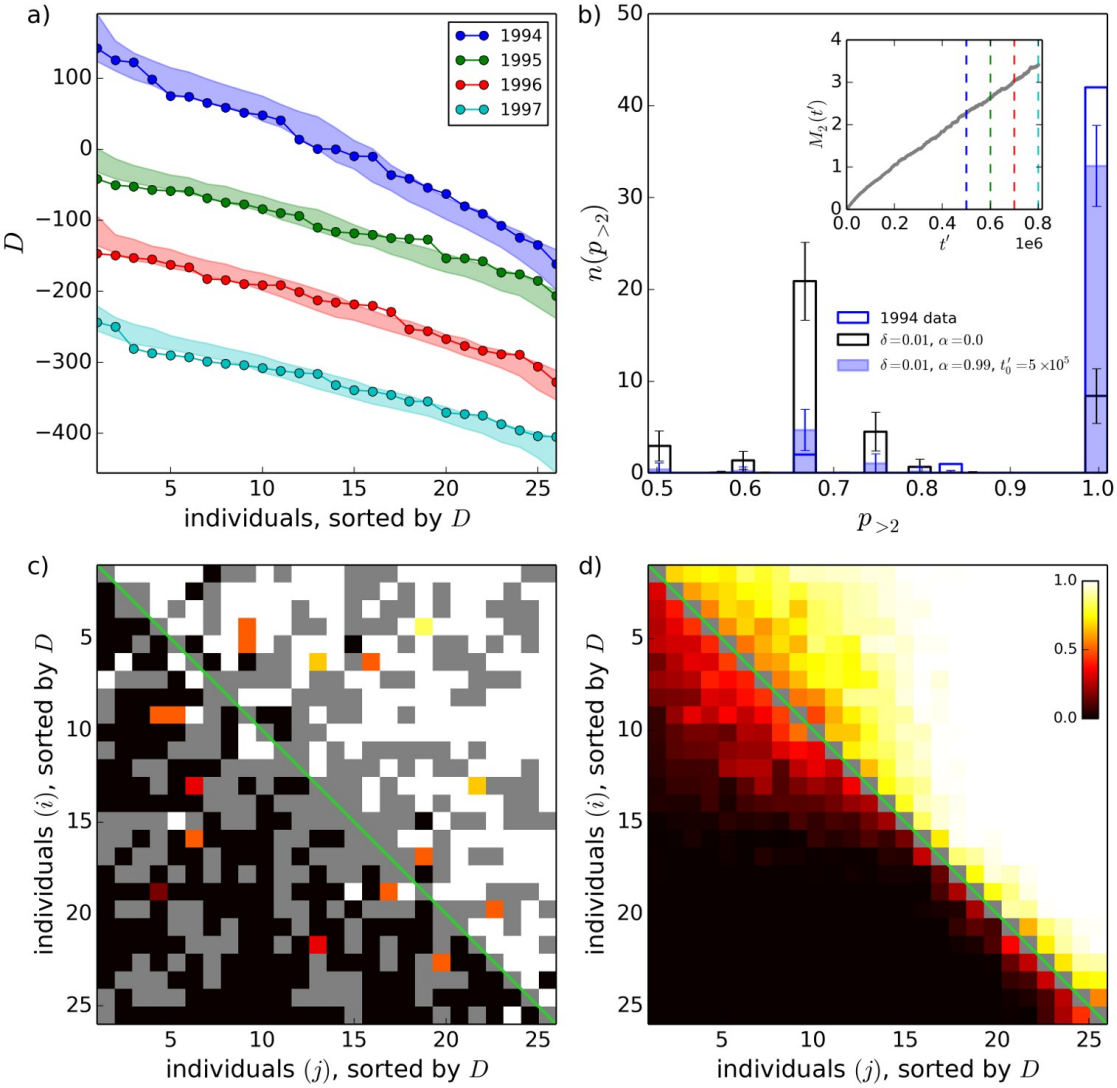
Comparison of model results with animal interaction data. (a) David’s Scores, *D*, calculated from the mountain goat interaction data of Ref. [56], for each of four summers from 1994-1997 (solid points). Individuals are ordered by decreasing *D* along the x-axis, and the value of *D* is plotted on the y-axis. The coloured bands show 5%–95% ranges for *D* calculated from interaction matrices obtained from simulations of the original (two-parameter) model with *δ* = 0.01, *α* = 0.99, and $t_{0}^{\prime }=5\times 10^{5}$ interactions, where $t_{0}^{\prime }=N\;t_{0}/2$ as per the definition of time in section 3.1. *n*_*r*_ = 100 realizations of the simulation were performed. (b) Histogram of *p*_>2_, the probability that the more successful individual won in a pairwise interaction, considering only those pairs of individuals that engaged in three or more interactions. *p*_>2_ was calculated from the interaction matrices corresponding to the blue points (animal data from 1994) and blue band (simulation) in panel (a), and for a simulation with *α* = 0 (black). For the simulations, the height of each histogram bar shows the average and the error bars show the standard deviation of the number of counts per bin. Inset of (b): *M*_2_(*t*) for the simulations from which the coloured bands in (a) and filled coloured histogram bars in (b) were obtained, showing (dashed coloured lines) the periods in the simulation during which interaction matrices were recorded. Panels (c) and (d): heatmaps showing individual *i*’s probability of defeating individual *j* in a pairwise interaction, calculated (c) from the 1994 mountain goat data, and (d) from the simulations from which the blue band in (a) was obtained (averaged over the *n*_*r*_ = 100 realizations). Grey squares indicate that no interaction occurred between *i* and *j*. Probability values indicated in the colour-bar in the top-right corner of (d) are applicable to both (c) and (d).

In Fig 8, we compare interaction matrices from an animal observational study with “simulated interaction matrices” from our model. We use the largest interaction matrices included in the recent review of Shizuka and McDonald [52], which were from a study of adult female mountain goats by Côté [56]. Côté published interaction matrices recorded over four summers, from 1994-1997, where *N* = 26 individuals were present in all four years. We ran simulations of the model for a system size *N* = 26, in which we recorded interaction matrices for the individuals in the simulation. These “simulated interaction matrices” were recorded over four separate time periods, where the duration of each time period was equal to the number of interactions recorded during each of Côté’s summers of observation (Côté recorded an average of 279 interactions/summer for the 26 goats, using ad libitum sampling). The simulated interaction matrices were each separated by a time period corresponding roughly to the number of pair-wise interactions that *N* = 26 female mountain goats are expected to have in one year, estimated here to be approximately 10^5^ interactions from the rate of 3 interactions/individual/hour found in Ref. [57]. We allowed each simulation to evolve to a time $t_{0}\gg \hat{\tau_{1}}$ before recording the first simulated interaction matrix, to bypass the initial transient evolution of the simulation away from the egalitarian initial condition. The mountain goat interaction matrices change very little from year to year. This corresponds to a very slowly evolving simulation, where the status distribution remains essentially unchanged between recordings of the simulated interaction matrices, highlighting again the relevance of the long-lived states observed in our model. This occurs for small values of the parameter *δ* (*δ* = 0.01 was used in Fig 8).

In Fig 8a, we show a comparison of the “David’s Score”, *D*, calculated from the simulated and real interaction matrices. *D* is a commonly-used score that allows a ranking of the individuals in an interaction matrix. It is defined as follows [53, 58]. Let *s*_*ij*_ be the number of times individual *i* has won in an fight against individual *j*, and let *n*_*ij*_ be the total number of fights between *i* and *j*. *P*_*ij*_ = *s*_*ij*_/*n*_*ij*_ is then the proportion of wins that *i* has experienced in fights with *j*. The proportion of losses that *i* has experienced in fights with *j* is 1 − *P*_*ij*_ = *P*_*ji*_, and when *n*_*ij*_ = 0, *P*_*ij*_ and *P*_*ji*_ are set equal to 0 [53]. Let $w_{i}=\sum_{j=1,j\neq i}^{N}P_{ij}$ and $w_{i,2}=\sum_{j=1,j\neq i}^{N}w_{j}P_{ij}$, such that the sum in *w*_*i*,2_ is weighted by the *w*_*j*_ of each opponent *j*. Similarly, let $l_{i}=\sum_{j=1,j\neq i}^{N}P_{ji}$ and $l_{i,2}=\sum_{j=1,j\neq i}^{N}l_{j}P_{ji}$. The David’s Score of an individual is *D*_*i*_ = *w*_*i*_ + *w*_*i*,2_ − *l*_*i*_ − *l*_*i*,2_. *D* thus depends not only on the proportions of wins and losses experienced by an individual, but also on the win and loss proportions of those with whom the individual has fought. For example, if an individual *i* defeats an opponent who has won a large proportion of his or her fights, this causes a large increase *D*_*i*_, whereas if *i* loses to an individual who has lost a large proportion of his or her fights, this causes a large decrease in *D*_*i*_.

Fig 8a shows that the set of *D*’s calculated from the simulated interaction matrices resembles the one from the mountain goat interaction matrices, and that this resemblance is maintained after allowing a large number of interactions (10^5^) to take place between recording the simulated interaction matrices. Fig 8b shows that, in the mountain goats, the more successful individual wins the fight almost all of the time, considering those pairs of individuals that engaged in three or more fights. A similar result was obtained from the simulation for the parameter values used in Fig 8a. Fig 8b also shows that, when *α* = 0 (such that *p* = 0.5 in Eq 1), there are few pairs for which the more successful individual wins all fights (as expected), such that *α* > 0 is required in order to have a good comparison with the animal data. Only those pairs of individuals that had engaged in three or more fights were considered in the histograms in Fig 8b, in order to avoid high fluctuations due to small numbers of fights. Also included in Fig 8 are heatmap plots showing the probability that individual *i* defeats individual *j* in a pairwise fight, calculated from the mountain goat interaction matrices (Fig 8c) and the simulated interaction matrices (Fig 8d), showing visually that reversals are rare but non-negligible.

Many animal observation studies have found similar interaction matrices to Côté’s mountain goat matrices, in that they have small numbers of reversals [52]. The results from our model therefore suggest that many animal groups have highly-skewed distributions of status and relatively large values of the authoritarianism, *α*. On the other hand, it is known that animal species with more complex social organization can have a larger number of reversals and intransitive relationships (where individual *i* wins the majority of fights against *j*, who wins the majority of fights against *k*, who in turn wins the majority of fights against *i*) [3, 59]. This may correspond to a less highly-skewed distribution of status in our model. Little is presently known about the frequency of reversals and intransitive relationships in large social groups due to a lack of interaction data for large groups. A key problem in this regard is that the proportion of pairs of individuals for which no interactions are observed generally grows with system size in interaction matrices from observational studies, due to the increasing difficulty of observing all pairs [52].

Due to the small value of *δ* required to obtain quasi-stationarity of the status distribution over large numbers of fights in the simulations in Fig 8, the model suggests that relatively small amounts of status (e.g., one one-hundredth of an individual’s current status when *δ* = 0.01) are exchanged in a single interaction. In female mountain goats, rank is strongly correlated with age, and older goats almost always win interactions against younger goats [56]. Larger values of *δ* and smaller values of *α* may apply in other animal societies in which ranks change more frequently or where age is a less important factor, including in some primate species in which complex power struggles leading to takeovers are commonly observed [60, 61].

We also note that for the *N* = 26 individuals present in all 4 years of Côté’s study, there was no tendency for individuals to interact more frequently with those close in rank than with those far away in rank, although such a tendency is observed when considering all individuals in a single summer, as shown in Fig 6 of Ref. [56] (see Section A in S2 Appendix for further details). Our subset of *N* = 26 individuals leaves out those individuals who enter the group (primarily, by ageing to the age of maturity of 3 years old) and who leave the group (primarily, by dying at old age) from one year to the next, which indicates that the tendency observed by Côté for individuals to interact more frequently with those closer in rank is mostly due to interactions involving the oldest and youngest individuals within the group. The absence of this tendency in the *N* = 26 subgroup that we consider supports our use of the original (two-parameter) model in the simulations in Fig 8.

Lastly, for the model simulations shown in Fig 8, the characteristic time *τ*_2_ ≈ 59 years, significantly longer than the 12-15 year life expectancy of mountain goats [62]. The ultimate collapse of the system to the totalitarian end-state exhibited by our model may therefore not be relevant for mountain goats, since factors such as births, deaths, maturation of juveniles, and immigration, which are not considered in our model but which occur on time-scales significantly shorter than *τ*_2_, will change the long-term dynamics of the system significantly.

### 4.2 Proxies for status in large social groups

In the comparison of our model with animal interaction data shown in section 4.1, we recorded the simulated interaction matrices starting from an arbitrary initial distribution of status, *S*(*t*_0_). In principle it is possible, for an arbitrary value of *α*, to estimate the {*S*_*i*_} values of the animals in an observed interaction matrix. This would require enough interaction data to estimate the win probabilities for a sufficient number of pairs of individuals such that the ratio *S*_*i*_/*S*_*j*_ could be calculated from Eq 1 for these pairs, and then all {*S*_*i*_} calculated from these ratios. In so doing, one of the {*S*_*i*_} can be set to an arbitrary value since the dynamics of the model are independent of the value of the (conserved) average status, $\bar{S}$, of the system. The data in published animal interaction matrices (including in Ref. [56]) is insufficient for this purpose, because there are few pairs of individuals where the win probability *p* < 1, making it impossible to obtain a meaningful estimate of {*S*_*i*_}. Given the difficulty of determining statuses of individuals in the model directly from observed interactions in animal behaviour studies, to compare our model results with real-world societies we seek a measurable quantity that can be used as a proxy for societal status in (large) social groups.

#### 4.2.1 Intraspecific body size distributions in social insects

We have reviewed a number of measurable quantities that may serve as proxies for societal status in non-human animal groups and these are presented in Table B in S2 Appendix. The only such quantity for which we were able to find data that would allow one to assign a status value to all individuals in a large group (*N* ≥ 100) is body size in social insects. Data collected and reviewed by Gouws and co-workers shows that body size distributions in social insects are typically right-skewed as opposed to being normally distributed in non-social insects [63]. The distributions of status formed in our model are also right-skewed (e.g. see Fig 2), and thus compare favourably with the body size distributions of social insects. While a more detailed comparison between simulated status distributions and intraspecific body size distributions in social insects would be desirable, it is currently problematic because published data either does not involve individuals from a single colony or does not contain enough information about the distribution to allow a comparison (specifically, both the variance and skewness of the proxy distribution are at least required).

While we were unable to find other proxy data for societal status in large groups of non-human animals, such data does exist for large groups of humans. We thus focus the remainder of this section on a comparison of the distributions of status produced by our model to a proxy for societal status (household income) in humans.

#### 4.2.2 Income distributions in humans

In humans, researchers use a theoretical construct called socioeconomic status (SES) to assign positions to individuals in the social hierarchy. Measurable characteristics such as income, education, occupation, and wealth are used as single-factor indicators of SES, or are combined in various ways to obtain composite indicators of SES [64]. When income data is available, it is considered to be a critical component in determining SES [65] and, when used as a single-factor indicator of SES, has been found to have greater explanatory power (for example, of the relationship between SES and mortality) than education or occupation [66]. Here, we use household income in Canada and the USA as a proxy for societal status, because income data is readily available in online datasets for these two countries (unlike data on wealth) and it is quantitative (unlike level of education or occupation), making it straightforward to analyze and compare to our model results. We use household income, rather than personal income, as our proxy for societal status, in order to avoid artifacts related to income sharing within a nuclear family unit, such as when a spouse decides not to work or to work below his or her maximum market value [67]. We thus follow Ref. [66] and many other studies (e.g. [68–70]) and use household income as an established proxy for socioeconomic status.

**Fig 9:**
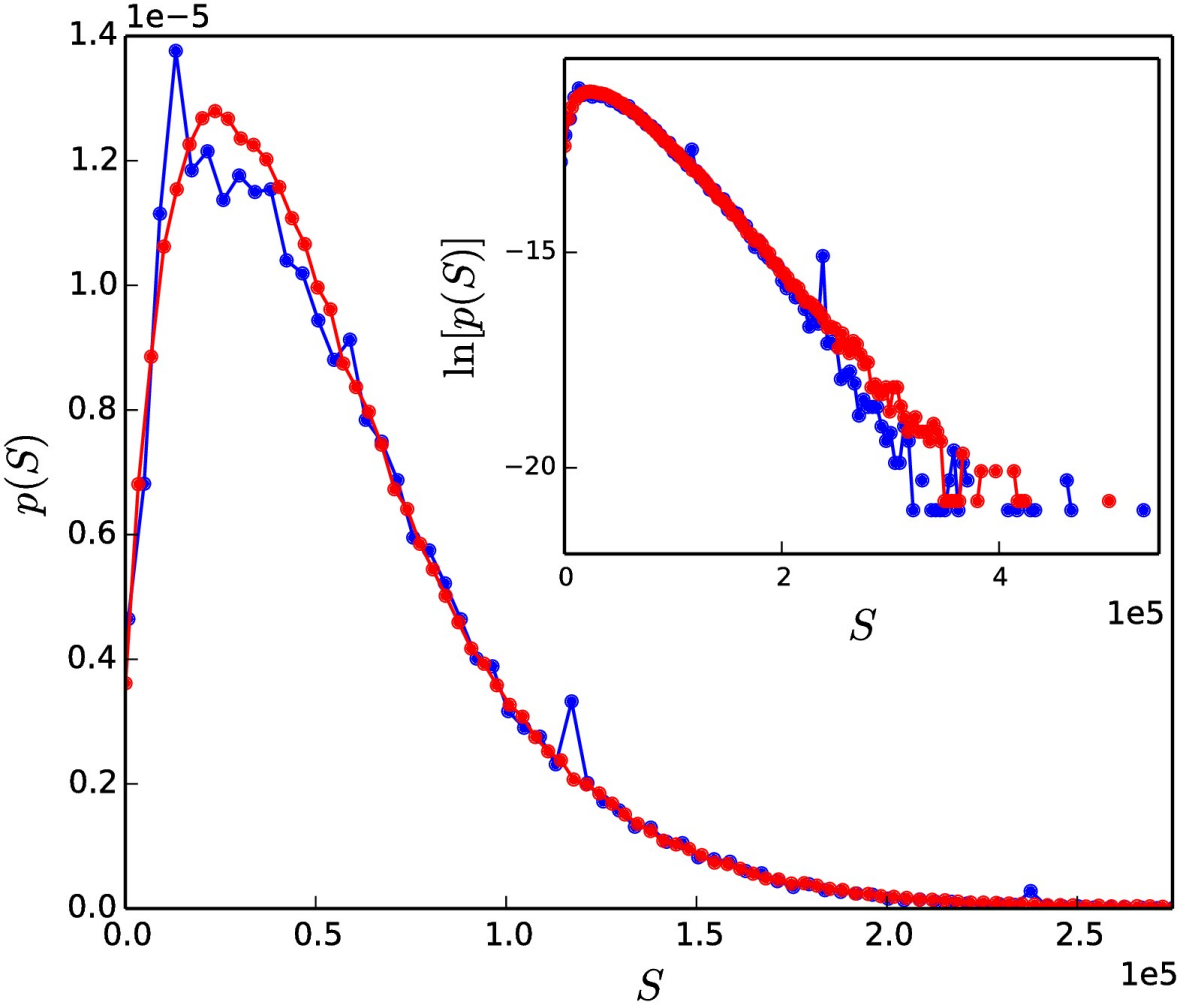
Fit of original (two-parameter) model status distribution to Canadian household income distribution. Simulated distribution (red curve, *δ* = 0.35 and *α* = 0), compared to the distribution of Canadian household incomes from the year 2000 (blue curve). Scaled variance, $M_{2}/\bar{S}=0.536$ and skewness, *γ* = 1.3 for the simulated distribution, and $M_{2}/\bar{S}=0.534$ and *γ* = 1.4 for the proxy distribution. The x-axis of the main plot has been cut at *S* = 2.5 × 10^5^. The large peak in the data at $12, 000 (*S* = 0.12 × 10^5^) is most likely due to welfare benefits provided by the government, and the peak at $12, 000 (*S* = 1.2 × 10^5^) comes from an upper income cutoff applied to the public-use data by Statistics Canada [72].

Fig 9 shows the (pre-tax) distribution of Canadian household incomes from the 2001 Canadian census [71], as well as a model-generated status distribution for the two-parameter version of our model (section 2). The simulated system contained *N* = 312, 513 individuals, which is the number of households in the public-use census sample. The average status of each individual was set equal to the average household income in the data, such that $\bar{S}=55,536$. The model parameter *α* was set equal to 0, in order to produce true steady-state status distributions, for convenience in obtaining a fit to the real data that is independent of observation time, *τ*_*obs*_. The parameter *δ* was then adjusted until a good fit was obtained with the income data (*δ* = 0.35 for the plot in Fig 9). We note that essentially the same status distributions—albeit not steady-state but only long-lived—can be obtained for (*δ*, *α*) values that trace out an equi-*M*_2_ line in *δ* − *α* parameter-space (see Fig 6 above and Section H in S1 Appendix).

The original (two-parameter) version of our model is sufficient to fit the Canadian household income distribution. This suggests that our very simple model may capture some essential features of the interactions that give rise to social hierarchy in large groups of individuals. The two model parameters, *δ* and *α*, which determine the outcomes of pairwise interactions, may be interpreted as societal features since their values are held constant for all interactions that occur within the society. Particular societies or species may have different values of one or both of these parameters, leading to different societal structures represented by the distribution of status. Additional comments about the potential real-world implications of the time evolution behaviour of the model are included in section 5.

A shortcoming of the Canadian data is that upper income cutoffs were applied to the public-use dataset by Statistics Canada, for the purpose of protecting confidentiality of wealthy individuals. This results in poor data quality at the upper income end of the Canadian household income distribution. However, there is evidence that income distributions from many countries have a low-to-middle-income part that is well described by a distribution function containing an exponential decay, and a separate, high-income part that decays more slowly [30, 73]. The inset of Fig 9 shows the Canadian household income distribution and the model-generated status distribution on a log-linear scale, in order to allow inspection of the large-*S* tail. The expected slower-than-exponential decay of the upper-tail is not seen in this data. This motivates us to examine the USA household income distribution, in which the distinct low-to-middle and upper income parts of the distribution are present.

**Fig 10:**
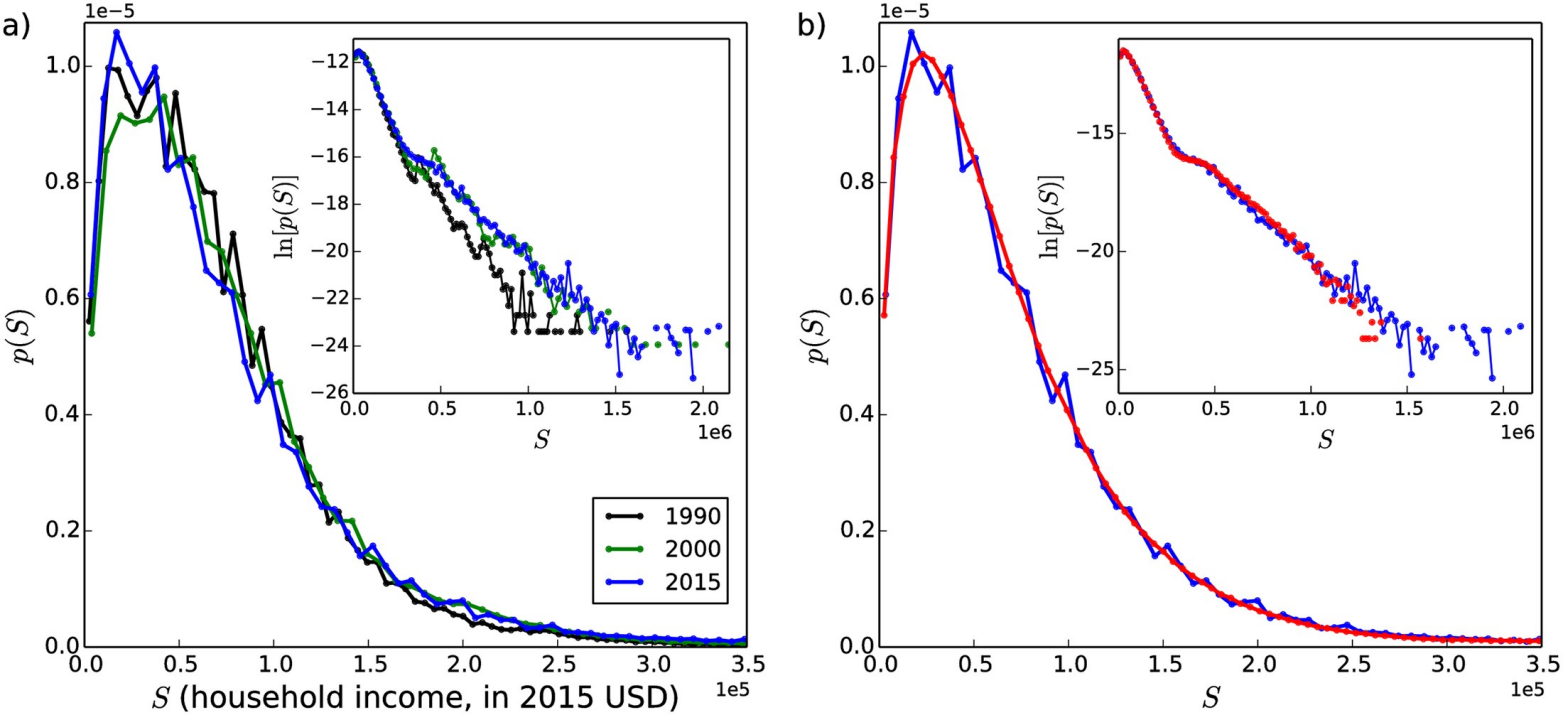
USA household income distributions and fit of extended model. (a) USA household income distributions for three different years (indicated in legend). Dollar values have been converted to 2015 values to allow comparison of the different datasets [75]. A “break” in the data separating the high-income tail from the low-to-middle part of the income distribution can be seen in the inset at *S* ≈ $250, 000 (*S* = 0.25 × 10^6^). Upper-income cutoffs have been applied to the American data by the governmental agency that provided the data, causing the plateau in ln[*p*(*S*)] (inset) for the highest income values. (b) Fit of extended model status distribution to USA household income distribution. Simulated distribution (red curve) with parameters *δ* = 0.4, *α* = 0, *η* = 3.5, *ϵ* = 0.08 compared to the distribution of USA household incomes from the year 2015 (blue curve).

Fig 10a shows United States household income distributions for the years 1990, 2000, and 2015 [74]. The USA datasets are 1-in-100 national random samples of the population. Dollar values for each of the three datasets have been adjusted to 1999 values in order to permit a comparison across time. In these distributions, the presence of an approximately exponentially-decaying low-to-middle-income part (initial straight-line decrease in the inset of Fig 10a beginning after the peak at *S* ≈ 0.25 × 10^5^ and ending at *S* ≈ 0.25 × 10^6^), separated from a more slowly-decaying high-income tail is visible. The “break” point between the lower and upper parts of the data is present for all three curves and can be seen in the inset of Fig 10a at *S* ≈ 0.25 × 10^6^. This break is observed in the income distributions of many different countries [30, 73].

Fig 10b shows the 2015 USA household income distribution and a simulated distribution from the extended model. The main plot (linear scale on the y-axis) shows that the simulated distribution fits well to the low-to-middle income part of the distribution. The inset (logarithmic scale on the y-axis) shows that the simulated distribution also contains a break between the low-to-middle and high-income parts of the distribution, mirroring the break in the data. The value of the parameter *η* was chosen such that $\eta \bar{S}=S_{B}$, where *S*_*B*_ is the location of the “break” point in the data, estimated from the data to be at $275, 000. Changes to the value of *ϵ* result in a poorer fit (see Figs C1 and C2 in S2 Appendix). As expected, *ϵ* is small, such that only 8% (*ϵ* = 0.08) of the pairwise interactions that would not occur according to the threshold criterion do occur. Two alternative ways to restrict the pairwise interactions between competing individuals that lead to quantitatively similar behaviour are described in Section E in S2 Appendix; for example, a simple, additional extension to the model that allows all individuals with statuses greater than $\eta \bar{S}$ to interact with each other shows an improved fit to the proxy data.

**Fig 11:**
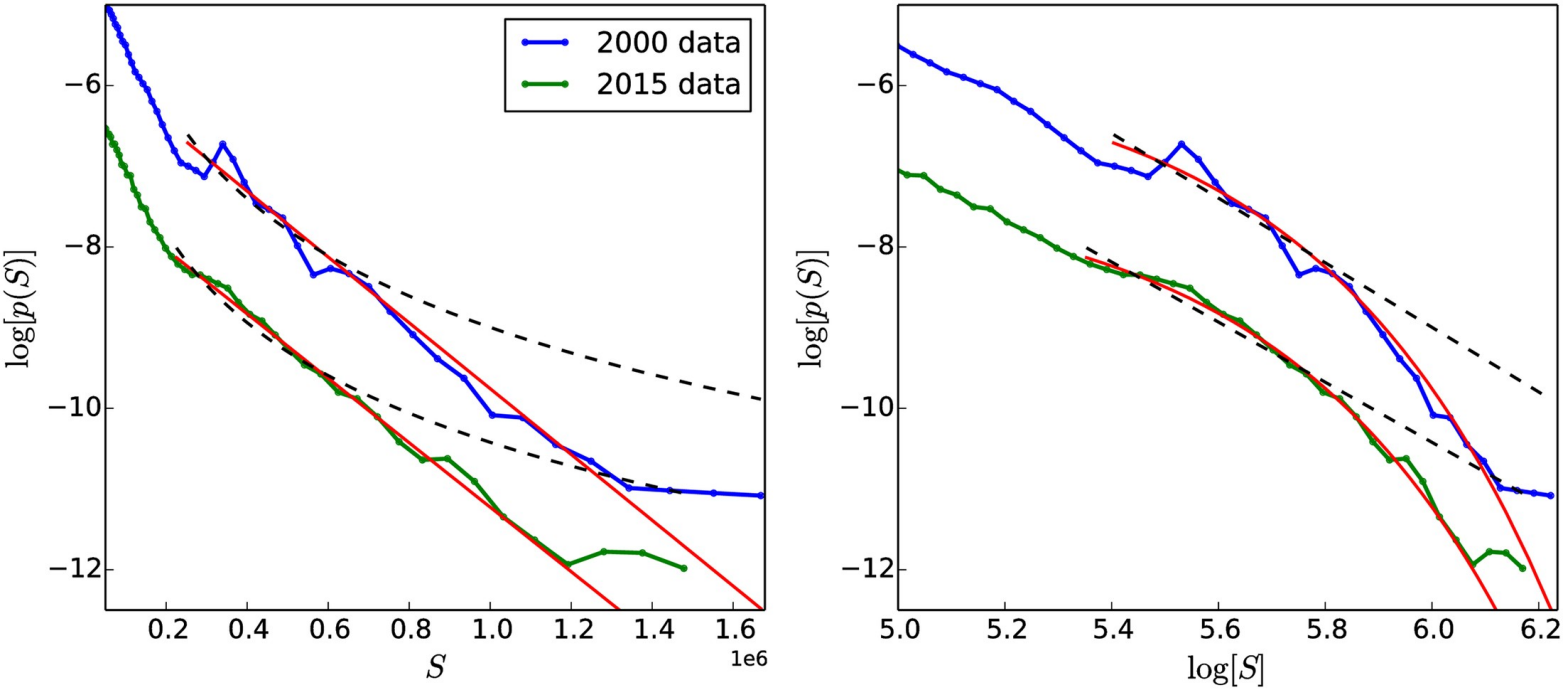
Functional form of high-income tail of USA household income distribution. Power-law (dashed black line) and exponential (solid red line) distributions with lower and upper bounds on the fitted distribution chosen to correspond to the full high-income tail. *S* represents USA household income data in 1999 USD. The curves for 2015 have been shifted down in the plots for better visualization.

Several econophysics studies have found power-law distributions in the high-income tail of personal income distributions [30, 73]. However, a graphical analysis (Fig 11) comparing best fits of exponential and power-law distributions to the high-income tails of the 2000 and 2015 USA household income distributions shows that the household income data that we use as a proxy for societal status is not consistent with a power-law distribution but is consistent with an exponential distribution over large ranges. This is also confirmed by Kolmogorov-Smirnov tests for both distribution types. Further details are provided in Section D in S2 Appendix [76–79].

The distinct low-to-middle and upper status parts of the data show the presence of two distinct groups or classes within the society. In the model, these two distinct groups emerge when the frequency of interactions between individuals with large differences in status is reduced. The model thus suggests that societal conditions that limit interactions between individuals with large differences in societal status may produce or maintain distinct social classes. Individuals belonging to the upper status class may have a self-interest in reinforcing such societal conditions in order to preserve their positions in society. Societies with policies that promote interactions between individuals with large differences in societal status may have less distinct class structures.

## 5 Discussion

We have presented a simple winner-loser model of the formation and evolution of social hierarchy, based solely on interactions (fights) between individuals that result in the transfer of societal status from the loser of the fight to the winner. We showed that the model exhibits regions in parameter-space in which the asymptotic distributions of status produced by the model either show a continuous unimodal behavior or take on a degenerate form, in which a single individual possesses all of the society’s status. In the latter case, intermediary distributions are long-lived for small positive values of the model parameters. Here, “long-lived” refers to quasi-stationarity of the status distribution, where, over a sufficiently short observation time, the status distribution remains essentially unchanged. This is quantified through the characteristic time *τ*_2_, which controls the evolution of the system toward the end-state.

Our model thus suggests that there are two fundamental characteristics of status-determining interactions in a society—the level of intensity of interactions (*δ*) and the degree of authoritarianism (*α*)—that determine both the outcomes of the interactions and whether the society’s structure will be stable or preserved for long times before undergoing eventual deterioration. These two parameters together with optional parameters restricting the interactions between individuals control the shape of the (intermediary) status distribution, which becomes more unequal (larger variance) as either *α* or *δ* is increased.

In comparing the status distributions produced by simulations of the original (two-parameter) and extended models with the proxies for societal status in section 4.2, the parameter *α* was set equal to 0. However, as shown in Section H in S1 Appendix, essentially the same long-lived status distributions can be obtained for (*δ*, *α*) values that trace out an equi-*M*_2_ line in *δ* − *α* parameter-space. Starting with a given value of *δ* and *α* = 0, one can locate an equi-*M*_2_ line in Fig 6. Then, following the equi-*M*_2_ line in the direction of increasing *α*, one eventually arrives at the transition between long-lived states and runaway (where the location of the transition depends on the time over which the society is observed). If the value of *δ* of a society can be determined by fitting the model (with *α* = 0) to a proxy for the society’s status distribution, then the corresponding equi-*M*_2_ line may provide an indication of the maximum level of authoritarianism for which essentially the same status distribution can be maintained over a specific time interval. This maximum level of authoritarianism would be reached when the equi-*M*_2_ line intersects with the boundary separating regions II and III for this specific time interval, see Fig 6 for examples.

Similarly, the presence of a characteristic time scale controlling the longevity of the intermediary distributions suggests a limit on the extent to which societal inequality can increase (e.g., due to societal changes that cause an increase of one or more of the parameters) before a runaway deterioration occurs. Whether a real society in fact approaches the end-state might depend on how this characteristic time scale compares with other time scales neglected in our model, such as the rate at which the society experiences external perturbations including wars with other societies or major environmental changes, and internal perturbations related to the effects of birth, death, immigration, and aging of individuals. For example, in the comparison of the model with agonistic interactions in animals shown in section 4.1, *τ*_2_ ≈ 59 years, significantly longer than the 12-15 year life expectancy of mountain goats [62], such that births, deaths, maturation of juveniles, and immigration may change the long-term dynamics of the system in such a way that it remains far from the end-state predicted by our model.

Extending our original (two-parameter) model by introducing an additional model rule that adjusts the probability with which individuals interact with one another based on the differences in their statuses, we can produce stable and long-lived status distributions which have identifiable low-to-middle and upper status regions. The status distributions in our extended model show good agreement with the distribution of household incomes in the USA (see Fig 10b), which we use as a proxy for societal status in large social groups. This appears to be the first model in which the two-part structure of the proxy distribution emerges by self-organization based solely on interacting individuals, without requiring any exogenous influence such as redistribution through taxation. Several analyses of personal income distributions have found that the low-to-middle-income part of the distribution decays exponentially and that the high-income tail decays like a power-law [30, 73]. Based on these analyses, various econophysics models have been propopsed with the goal of generating distributions of income with two-part shapes in which the lower-to-middle part of the income distribution follows an exponential distribution, and the upper tail follows a power-law distribution [39, 80, 81]. Among the models that generate a distribution of income or wealth as a self-organizing process based on interactions between individuals, several are able to produce either a power-law decay in the upper tail, or an exponential decay in the lower part of the distribution, but not both. The status distributions produced by the original (two-parameter) version of our model do not have two-part structures, and therefore never contain a “break” in the large-*S* tail similar to that seen in the insets of Fig 10. But the extended version of our model can produce two-part structures, where both the regime leading up to and the regime following the “break” decay exponentially. Our model thus suggests that societal structures containing distinct social classes arise when interactions between individuals with large differences in social status are limited or restricted, as occurs, for example, in residential segregation in the USA [45, 82, 83].

A necessary foundation for more advanced studies of social hierarchy is the exploration of the simplest possible realistic models, including the determination of their limits. This was the goal of the present article. Future, network-oriented models may incorporate features such as the histories of interactions between individuals [84–86] and cooperative behaviours including the formation of coalitions [43, 87–89] and mobbing [90, 91]. Such models may provide deeper understanding about the origins and evolution of societal structures, including the mechanisms responsible for societal destabilization or collapse.

## Supporting information

S1 Appendix Supporting information for section 3. (PDF)

S2 Appendix Supporting information for section 4. (PDF)
