# Supplementary material for: Self-organization and time-stability of social hierarchies
Source: PLoS One. 2019 Jan 29;14(1):e0211403. doi: 10.1371/journal.pone.0211403 (PMC6350989; doi:10.1371/journal.pone.0211403)
Supplement: S1 Appendix — (PDF) [file pone.0211403.s001.pdf]

S1 Appendix

Joseph Hickey and Jörn Davidsen

A Comparison of simulation results to Ispolatov et al. analytic solution

In order to demonstrate that the definition of time as $t = 2t'/N$ in the simulation corresponds to the definition of time in the analytical solution of Eq 3 of the main text, we show (Fig A1) a plot of $M_2(t)$ when the parameter $\alpha = 0$ with time defined as $t = 2t'/N$. Each cluster of curves in the figure corresponds to a particular value of the model parameter δ. Within a single cluster of curves, simulation results are shown for three different system sizes, N. The red line shows a fit of Eq 5 of the main text, where the fit parameters τ_1 and c_1 are equal to their corresponding analytic values from Eq 3 of the main text. For example, for $\delta = 0.8$ and $\bar{S} = 1$, the fit parameters are $c_1 = 4.00$ and $\tau_1 = 6.29$, whereas Eq 3 of the main text gives $\hat{c_1} = 4$ and $\hat{\tau_1} = 6.25$.

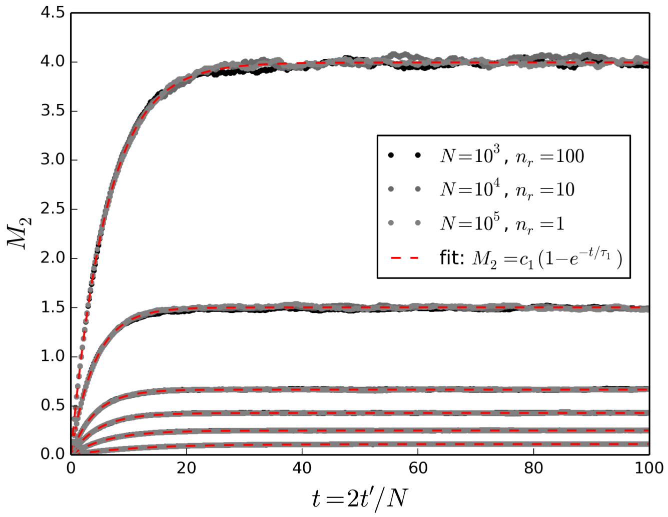

Fig A1 – Evolution of $M_2(t)$ for $\alpha = 0$ and various values of δ (from bottom, δ = 0.1, 0.2, 0.3, 0.4, 0.6, 0.8). Time is scaled so that $t = 2t'/N$. Fits are for $N = 10^5$. Data averaged over n_r realizations of the simulation.

B Time evolution of distributions

B1 Beginning from an egalitarian initial condition

Fig B1 shows the time evolution of systems prepared in an "egalitarian" initial condition, in which all individuals have the same initial status of $S = 1$. Each row in the figure corresponds to a different value of δ, and each column to an instant in time, with time increasing from left to right across the subplots. For all values of δ, the system undergoes an early-time evolution away from the initial condition. When $\alpha = 0$ (blue curves), the system arrives at a steady-state in which the distribution of status remains constant in time. The steady-state distribution is obtained within a time equal to a few multiples of $\hat{\tau_1}$, as expected from Eq 5 of the main text. When $\alpha > 0$ (green and red curves), the distribution continues to evolve after the transient period, and approaches the totalitarian end-state at a rate that depends on δ and α.

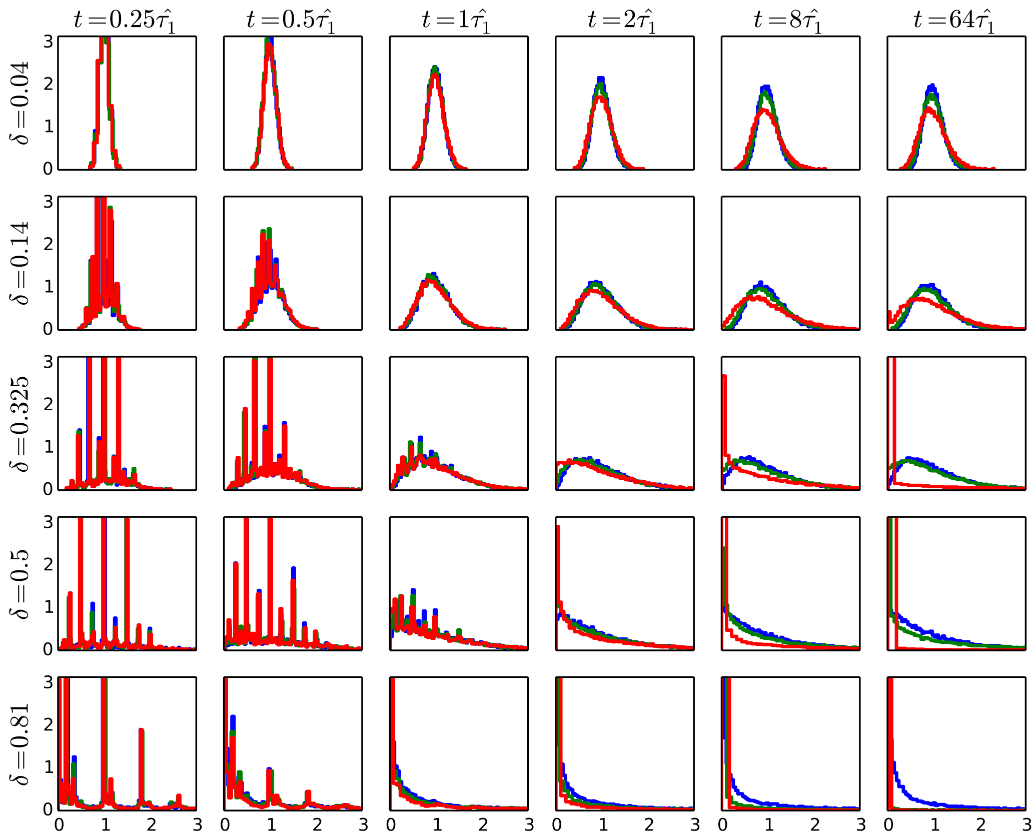

Fig B1 – Time evolution of distributions for different values of δ, with $\alpha = 0$ (blue), $\alpha = 0.2$ (green), and $\alpha = 0.5$ (red), starting from an initial condition in which all individuals have status $S = 1$ ("egalitarian initial condition"). x-axes in the sub-plots correspond to status, S, and y-axes to the probability density, $p(S)$. $N = 10^3$, $n_r = 20$.

B2 Beginning from a uniform initial condition

Figures showing the evolution of the model beginning from a uniform initial condition are included below, for the same values of δ and α shown in Fig. B1. In the uniform initial condition, the initial status of each

individual is chosen randomly from a uniform distribution with average status $\bar{S} = 1$. The time evolution beginning from this initial condition is qualitatively the same as for the egalitarian initial condition.

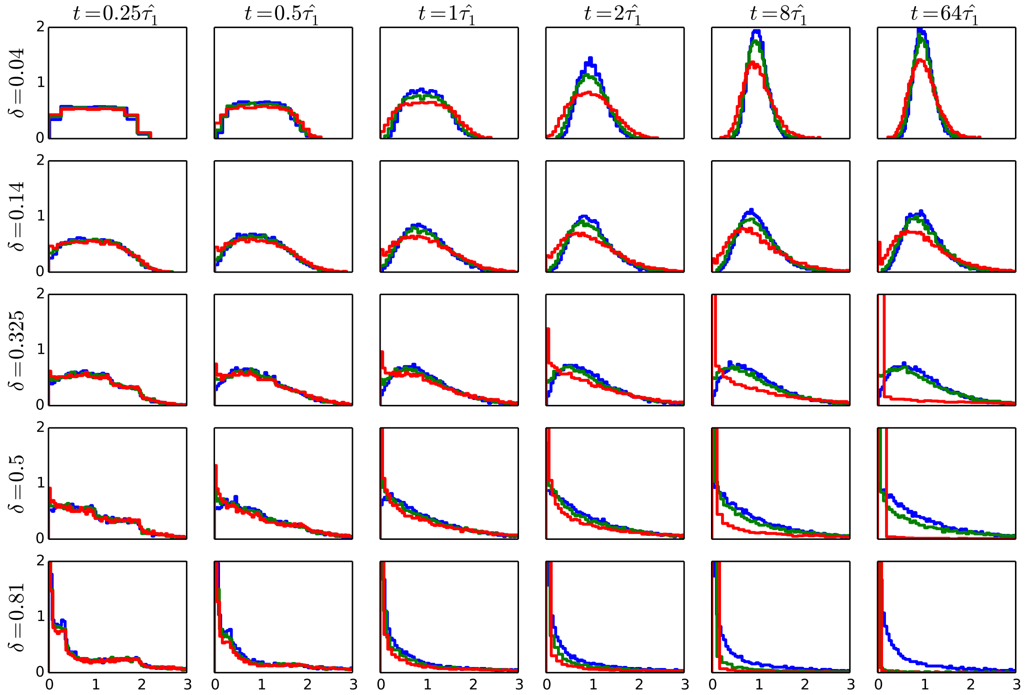

Fig B2 – Time evolution of distributions for different values of δ, with $\alpha = 0$ (blue), $\alpha = 0.2$ (green), and $\alpha = 0.5$ (red), starting from an initial condition in which status is uniformly distributed ("uniform initial condition"). x-axes in the sub-plots correspond to status, S, and y-axes to the probability density, $p(S)$. $N = 10^3$, $n_r = 20$.

C Behaviour of τ_1 and τ_2 as a function of system size, N

In order to examine the behaviours of the characteristic times τ_1 and τ_2 as a function of system size, N, plots of $M_2(t)$ for different system sizes are shown in Fig C1. The parameters controlling the early-time behaviour of M_2 (c_1 and τ_1) remain constant as system size, N, is increased. The parameters controlling the long-time behaviour of M_2 increase linearly with N. For example, for $\alpha = 0.5$, the values of τ_2 extracted from a fit of Eq 7 of the main text are 1.17×10^5, 1.18×10^6, and 1.21×10^7 for system sizes $N = 10^2$, 10^3, and 10^4, respectively. Likewise, the parameter c_2, which represents the "upper plateau" end-state value of M_2 increases linearly with N in the large-N limit (Eq 6 of the main text).

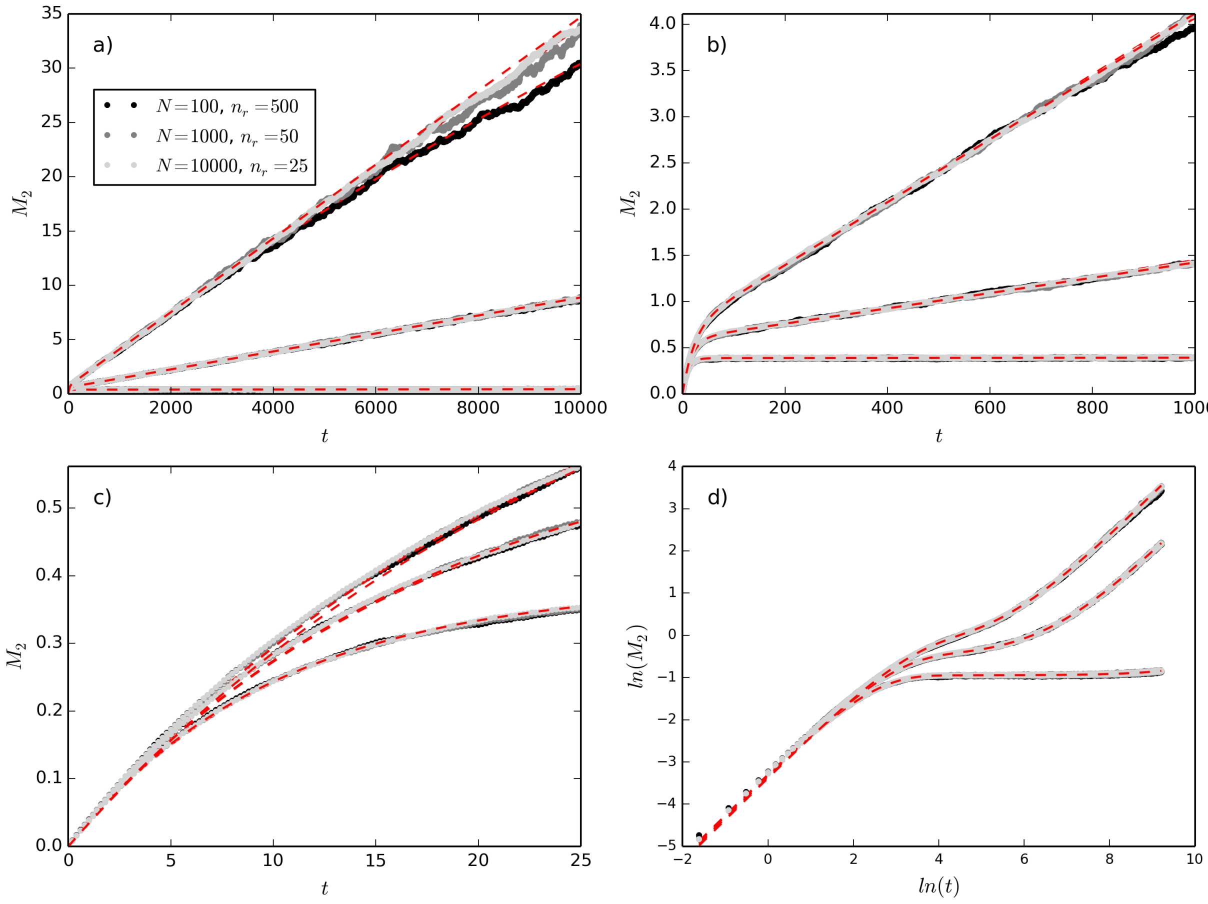

Fig C1 – Evolution of $M_2(t)$ for $\delta = 0.2$ and $\alpha > 0$ (from bottom, $\alpha = 0.3$, 0.5, 0.6) for different system sizes N. (a)-(c) different (linear) scales on x and y axes. (d) log-log scale. Fits are of Eq 7 of the main text (red dashed lines) to simulated data.

D Proof that N^2 fights are required to reach end-state when $\delta = 1$ and $\alpha = \infty$

In order to explore how the long-time behaviour of the evolution of the status distribution depends on system size N, we consider the extreme scenario of a system in which the higher-status individual in each fight wins with probability $p = 1$, and in which the winner of each fight receives all of the loser's status, leaving the loser with zero status. This extreme scenario corresponds to $\delta = 1$ and $\alpha = \infty$. In the model presented in the main text, δ is strictly less than 1, meaning that no single individual can ever possess all of the societal status of the system. However, in the following we set $\delta = 1$ in order to allow the system to obtain (after a finite time and for a finite system size) the end-state in which a single individual possesses all of the societal status and all other individuals have zero status. This simplifies the analysis, leading to the proof described below.

The goal of this exercise is to determine the time, τ_{end}, required to reach the totalitarian end-state beginning from an egalitarian initial condition. To do so, we will work backwards from the totalitarian end-state, in which a single individual possesses all of the societal status.

Working backwards from the end-state, we can consider the sequence of events that were required to arrive at the end-state. The first required event, working backwards, is a fight between two individuals possessing non-zero status. Since $\delta = 1$, one of these individuals loses and exits the fight with status equal to zero. The other individual wins the fight and receives all of the status of the loser, which, added to its pre-fight status, equals the total status of the system. Therefore, the end-state is preceded by a configuration of the system in which only two individuals possess non-zero status. The probability that these two particular individuals are selected to fight each other is the probability of selecting the first individual OR the second individual AND the probability of selecting the remaining individual:

$$\rho_2 = \left(\frac{1}{N} + \frac{1}{N}\right)\frac{1}{N-1} = \frac{2}{N(N-1)}. \tag{S1.1}$$

Continuing to work backwards, in order to obtain the configuration of the system in which there are two individuals with non-zero status, a preceding configuration having three individuals with non-zero status is required. The probability that two of these three individuals are selected to fight is:

$$\rho_3 = \binom{3}{2}\frac{2}{N(N-1)}. \tag{S1.2}$$

Following through with this logic, we find $\rho_N = \binom{N}{2}\frac{2}{N(N-1)}$. Now, the expected number of fights that will elapse between the required configuration k and the subsequent required configuration $k-1$ in this sequence is ρ_k^{-1}. Therefore, the expected number of fights to reach the end-state is:

$$\begin{aligned}\tau'_{end} &= \frac{N(N-1)}{2}\left(\frac{1}{\binom{2}{2}} + \frac{1}{\binom{3}{2}} + \frac{1}{\binom{4}{2}} + \ldots + \frac{1}{\binom{N}{2}}\right) \\ &= N(N-1)\sum_{k=2}^{N}\frac{1}{k(k-1)}. \end{aligned} \tag{S1.3}$$

The sum in Eq. S1.3 approaches 1 for large N, such that $\tau_{end} \approx N^2$. The time to reach the end-state when $\delta = 1$ and $\alpha = \infty$ is therefore $\tau_{end} \approx 2\tau'_{end}/N = 2N$, for large N.

This proof is included as a demonstration of the configurational reasons why the long-time (approach to the end-state) evolution of the model dynamics increases in proportion to the system size N.

E Phenomenology of the time-evolution of status distributions when $\alpha > 0$

In this section we provide some additional details about the phenomenology of the evolution of the status distributions when $\alpha > 0$.

Fig E1a shows a plot of $M_2(t)$ for a system with $N = 1000$, $\delta = 0.2$, and several values of α.

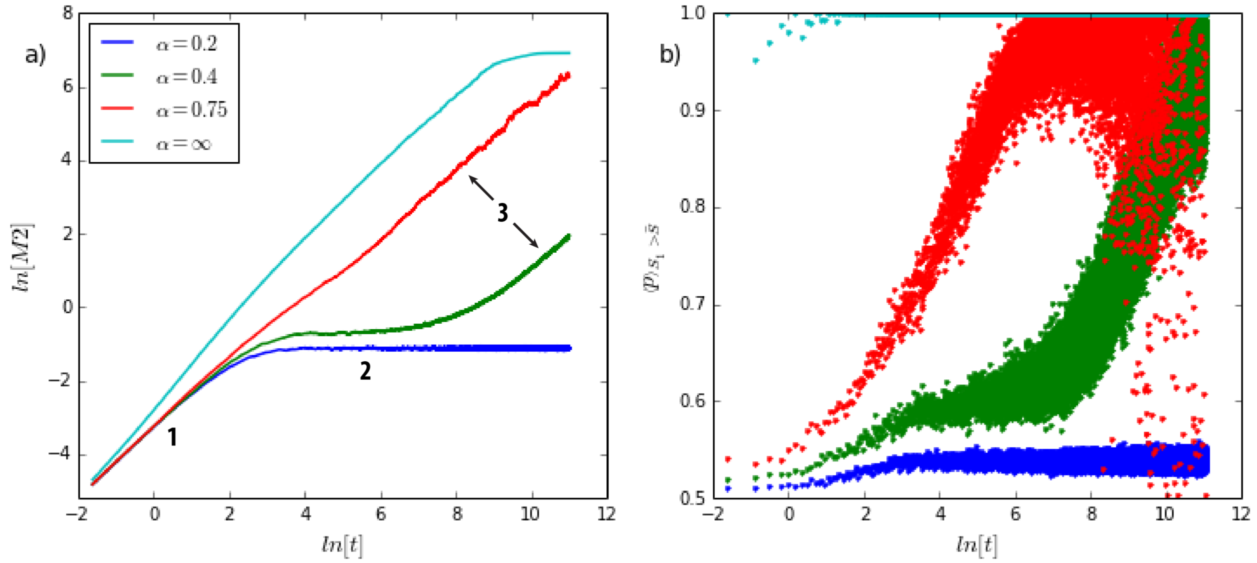

Fig E1 – (a) $M_2(t)$ on a log-log scale, with four stages of evolution indicated. $\delta = 0.2$ for all curves. (b) Probability p (averaged over preceding 100 fights, considering only those fights in which $S_1 > \bar{S}$) as a function of time. $N = 1000$ (a and b) and $n_r = 10$ (a) and $n_r = 1$ (b)

Three distinct stages in the evolution of $M_2(t)$ can be identified, as indicated on the log-log plot of $M_2(t)$ shown in E1a. First (stage 1), there is a rapid change away from the initial condition into a distribution similar in shape to the $\alpha = 0$ (true steady-state) distribution. This distribution shape is essentially maintained during stage 2, such that $M_2(t)$ changes only very slowly with time. For example the curve corresponding to $\alpha = 0.2$ (blue) in Fig E1a remains in stage 2 over the time scale of the simulation (to demonstrate this, distributions corresponding to specific points in time along this curve are shown in Fig E2). The duration of stage 2 decreases with increasing α, as can be seen in the curve corresponding to $\alpha = 0.4$ (green) in Fig E1a (distributions corresponding to specific points in time along this curve are shown in Fig E3). When α is increased further, stage 2 essentially disappears (red curve in Fig E1a – distributions corresponding to specific points along this curve are shown in Fig E5).

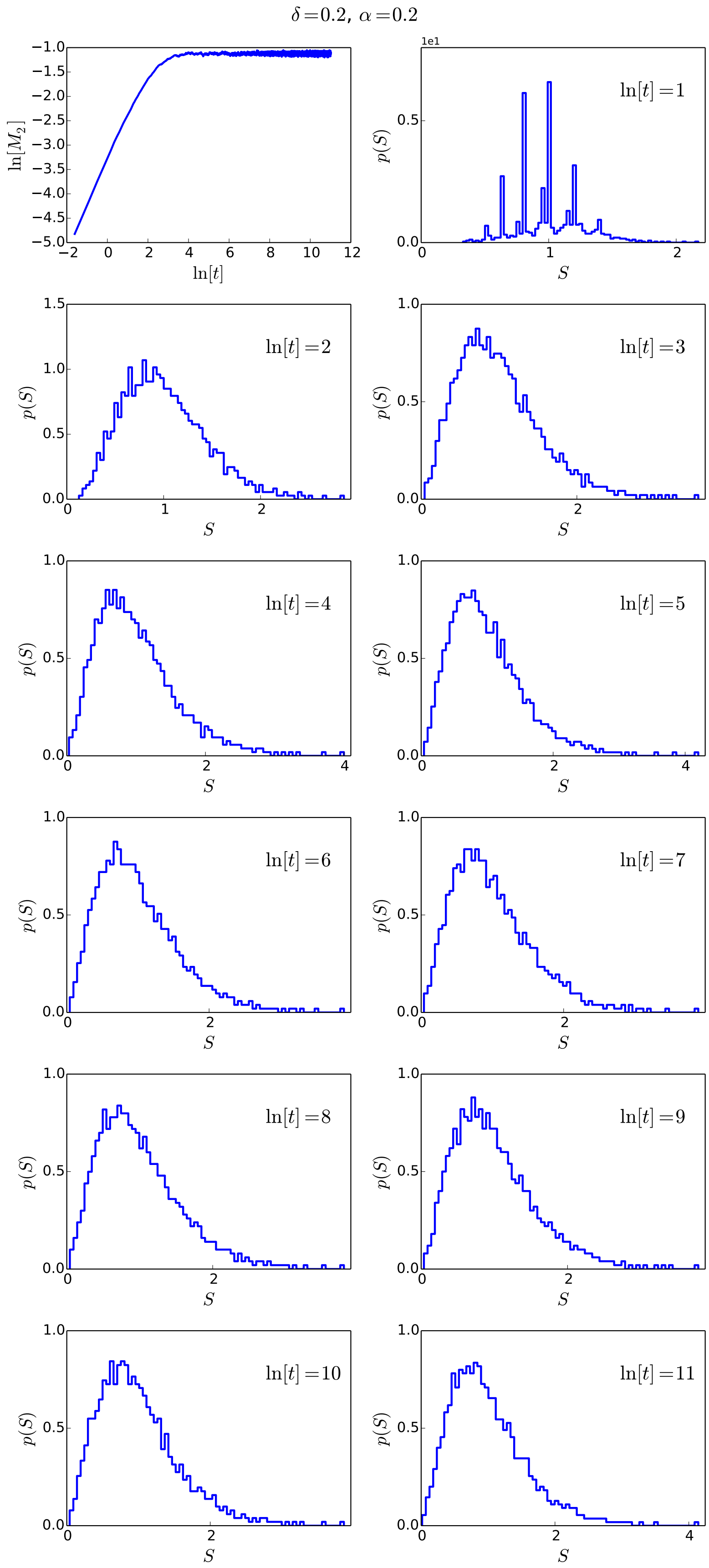

Fig E2 – Time evolution of distributions for $\delta = 0.2$, $\alpha = 0.2$. $N = 1000$, $n_r = 10$.

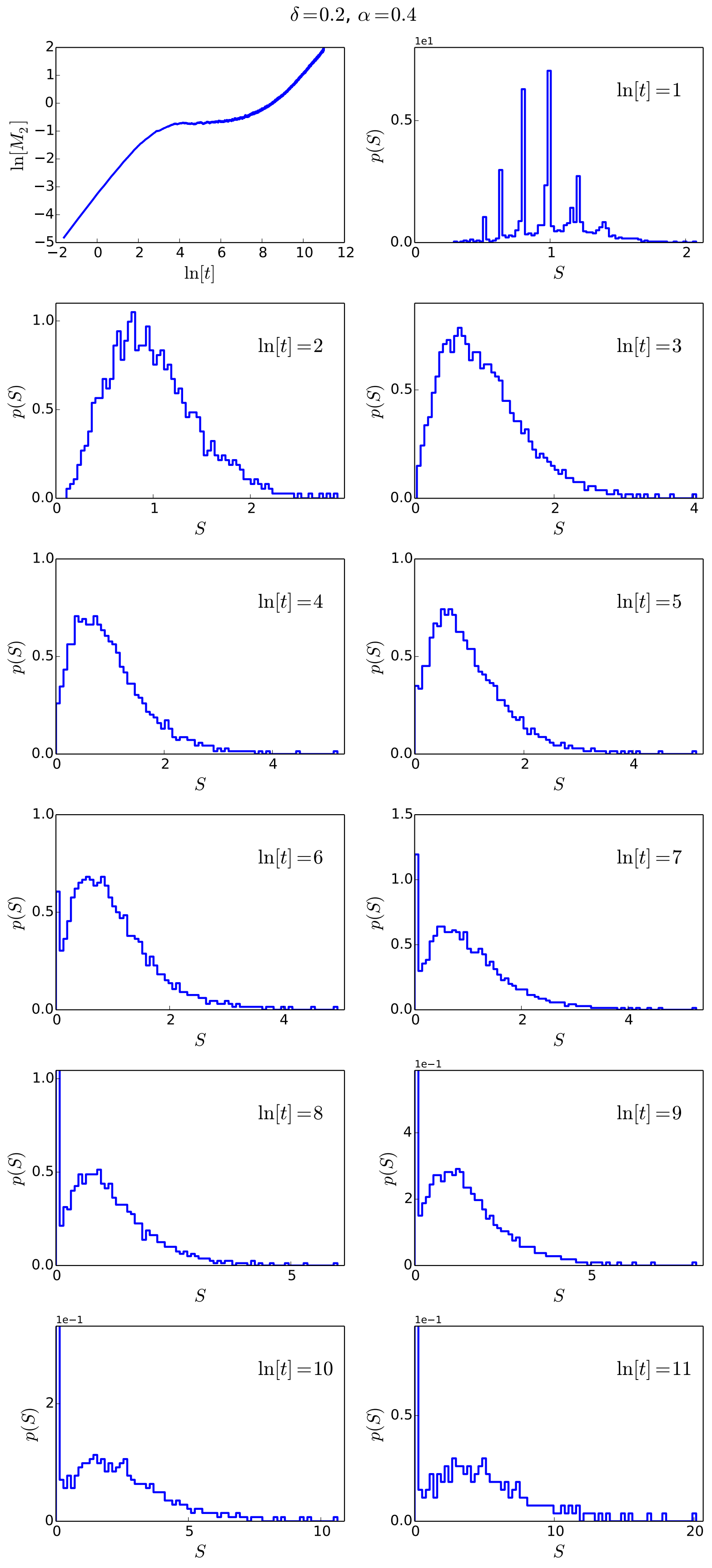

Fig E3 – Time evolution of distributions for $\delta = 0.2$, $\alpha = 0.4$. y-axis scale adjusted in plots for $\ln[t] = 8$ to 11 to allow visualization of large-S range of distribution. $N = 1000$, $n_r = 10$.

Transitioning from stage 2 to stage 3, the distribution accumulates many individuals with very low statuses, and several higher-status individuals emerge. Stage 3 then consists of the slow evolution of this highly skewed distribution. A particular characteristic of stage 3 is that, for fights involving at least one individual who still retains sizeable status (e.g., $S_1 > \bar{S}$), the probability $p \to 1$. This is illustrated in Fig E1b, in which p is averaged over the preceding 100 fights, considering only those fights in which $S_1 > \bar{S}$, giving the quantity $\langle p \rangle_{S_1 > \bar{S}}$. The fact that $\langle p \rangle_{S_1 > \bar{S}} \to 1$ signifies that the higher-status individual in any given fight (excluding fights in which neither individual has greater than average status) is virtually guaranteed to win. For finite values of $\alpha > 0$, in order for this to occur, there must be a large relative status between the two individuals. Stage 3 is therefore characterized by a distribution in which the ratio of S_2/S_1 for any two randomly-selected individuals is small enough such that $\langle p \rangle_{S_1 > \bar{S}} \to 1$ for fixed α (as per Eq 1 of the main text). The large-time decrease in $\langle p \rangle_{S_1 > \bar{S}}$ (e.g. for the red curve corresponding to $\alpha = 0.75$ in Fig E1b, at $\ln[t] = 8$ to $\ln[t] = 11$) occurs as individuals with $\bar{S} < S < S_H$ begin to experience decreases in status. Here, S_H is the status of the highest-status individual. This is illustrated in Fig E4, which plots the sum of statuses of all individuals with $\bar{S} < S < S_H$ as a function of time.

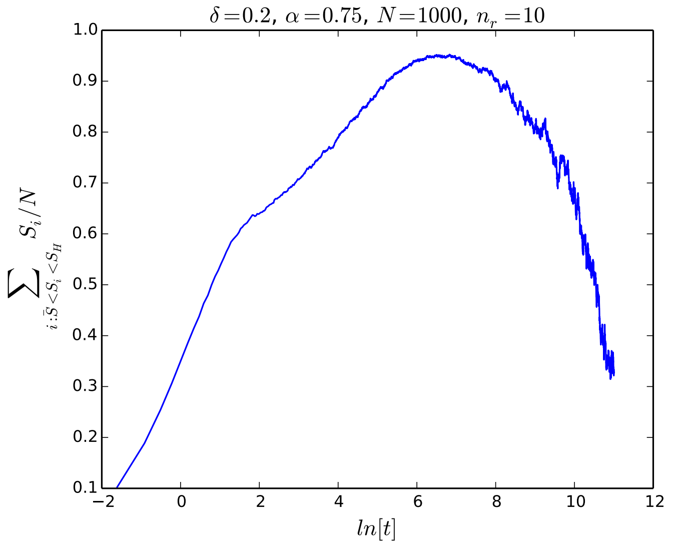

Fig E4 – Sum of statuses of all individuals with $\bar{S} < S < S_H$ vs. t, for $\delta = 0.2$ and $\alpha = 0.75$. $N = 1000$, $n_r = 10$.

The transition from stage 3 to the totalitarian end-state involves the decrease in the sum of statuses shown in Fig E4 following the maximum at $\ln[t] \approx 7$. This shows that in its evolution toward the end-state, the distribution must pass through a series of configurations (stage 3) in which there are a number of individuals with higher-than-average statuses, these individuals having extracted their large statuses from the mass of very low status individuals that accumulates in the transition from stage 2 to stage 3. Only after stage 3 has been obtained are the conditions present in which fights between high status individuals can lead to the emergence, in the end-state, of a single individual with status approaching the total status of the system.

In the end-state, $S_H \to N\bar{S}$ and $M_2(t) \to c_2$. The fourth (light blue) curve in Fig E1 corresponds to $\alpha = \infty$, signifying that the higher-status individual in any fight is guaranteed to win (several points in Fig E1b for which $\langle p \rangle_{S_1 > \bar{S}} < 1$ for this curve result from fights in which $S_1 = S_2$ due to the egalitarian initial condition). Distributions at specific points along this curve are shown in Fig E6.

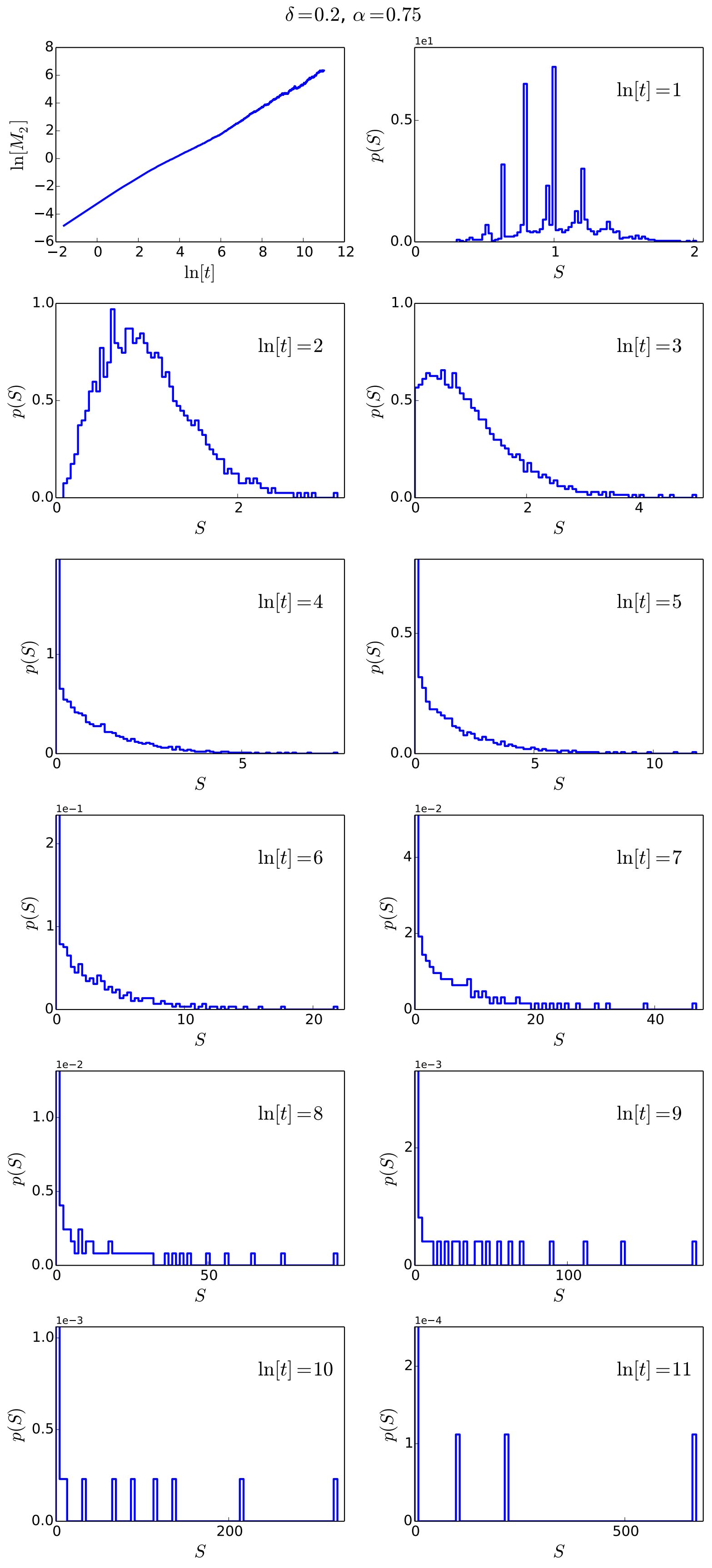

Fig E5 – Time evolution of distributions for $\delta = 0.2$, $\alpha = 0.75$. y-axis scale adjusted in plots for $\ln[t] = 4$ to 11 to allow visualization of large-S range of distribution. $N = 1000$, $n_r = 10$.

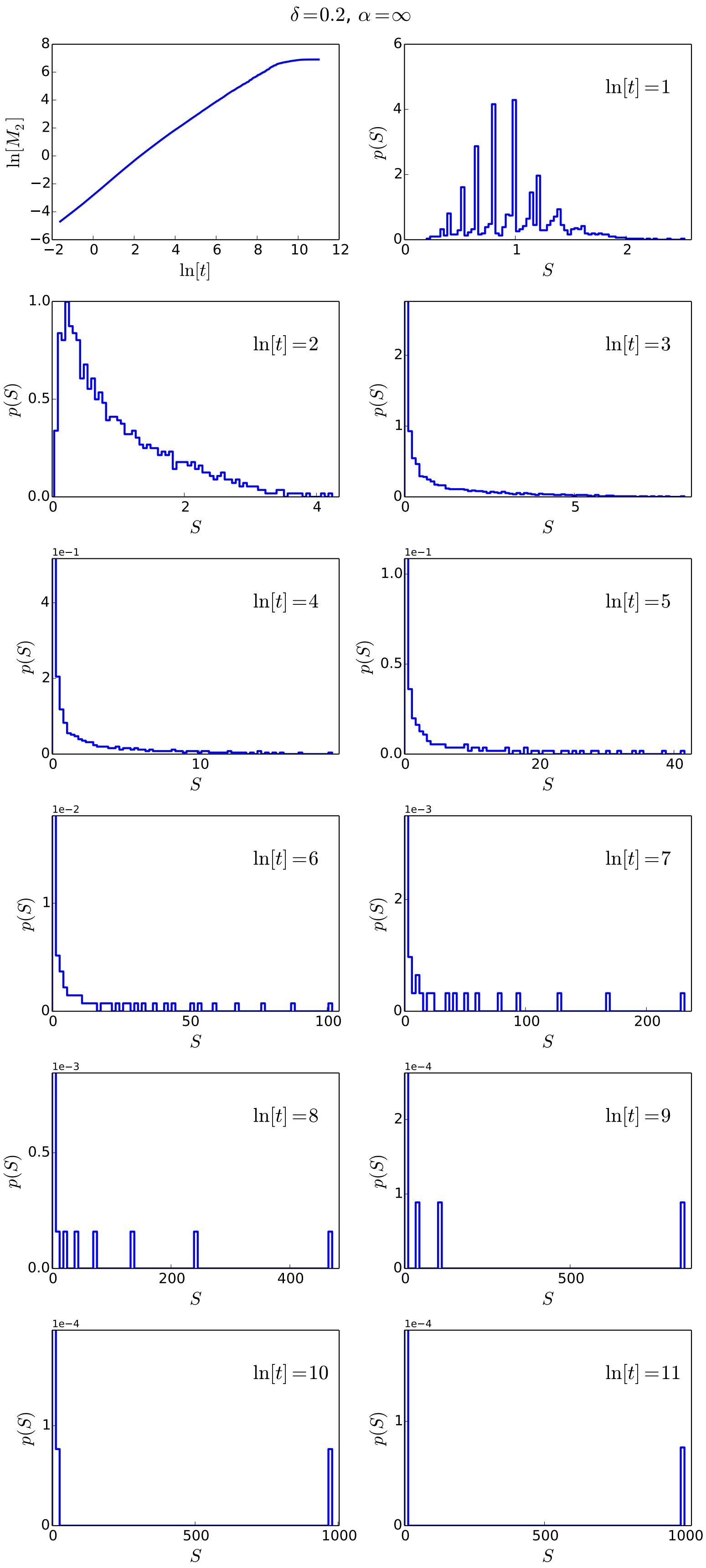

Fig E6 – Time evolution of distributions for $\delta = 0.2$, $\alpha = \infty$. y-axis scale adjusted in plots for $\ln[t] = 3$ to 11 to allow visualization of large-S range of distribution. $N = 1000$, $n_r = 10$.

A plot showing the same quantities as in Fig E1, but where α is fixed and δ is varied, is included below for comparison. As can be seen, as δ is increased, the duration time of stage 2 decreases.

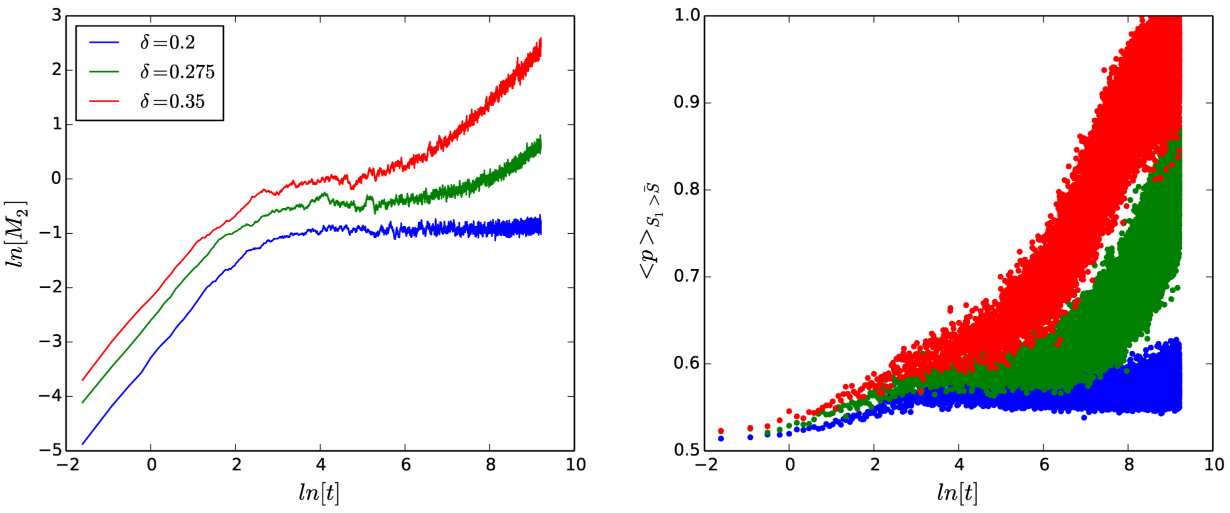

Fig E7 – (a) $M_2(t)$ on a log-log scale. (b) Probability p (averaged over preceding 100 fights, considering only those fights in which $S_1 > \bar{S}$) as a function of time. $\alpha = 0.3$ for all curves. $N = 1000$, $n_r = 10$.

F Proof that a single individual with non-negligible status remains in the totalitarian end-state

When $\alpha > 0$, the system approaches an end-state in which a single individual possesses virtually all of the society's status and all other individuals have status approaching zero. An argument that only a single individual with non-negligible status remains in the end-state (and not, for example, multiple high-status individuals), is as follows.

Consider a large (but finite) system of N individuals. When $\alpha > 0$, beginning from an egalitarian initial condition, the system evolves such that there are relatively few individuals with large status and relatively many individuals with small status (see section E). Since the average status of the system is constant, we can use it as a reference point to define "large status" to mean greater than average status, and "small status" to mean less than average status. The subsequent dynamics can be understood in terms of two timescales. The first timescale, λ_1, sets the time (measured in number of fights) that transpires, on average, between fights involving the same two particular individuals. Since pairs of individuals are picked randomly from among the population, λ_1 is determined by the system size N and remains unchanged as the system evolves.

The second timescale, λ_2, relates to the time required for an individual with less than average status to achieve an upset victory over an individual with greater than average status. Once the system has evolved to a point in which there are few individuals with high statuses and many individuals with low statuses, such an upset is the only way in which an individual with lower than average status can obtain greater than average status. λ_2 is determined by the average probability, $\langle p_{upset} \rangle$, with which an upset occurs (the smaller $\langle p_{upset} \rangle$, the larger λ_2). The probability for such an upset to occur (in any particular fight) is less than 50%, by Eq 1 of the main text. Therefore, fights that involve one individual with greater than average status and one individual with less than average status will generally result in a transfer of status from the lower-status individual to the higher-status individual. This creates a net transfer of status from individuals with lower than average status to individuals with greater than average status. One outcome of this net transfer of status is a reduction in $\langle p_{upset} \rangle$. Consequently, the timescale λ_2 must increase with time (number of fights).

Since λ_1 remains constant whereas λ_2 increases as the system evolves, eventually λ_1 dominates the dynamics such that any two particular individuals fight one another much more frequently than any one particular individual with lower than average status achieves an upset that transforms it into an individual with greater than average status. Since fights between any two particular high-status individuals occur with higher frequency than fights in which a low-status individual achieves an upset over a high-status individual, there is a net flow of the losers of fights between two high-status individuals into the ranks of those with lower than average status.

The rate at which this net flow of losers occurs increases as λ_2 increases, and after an infinite number of fights ($t \to \infty$), only a single individual with greater than average status remains.

G Technical aspects regarding τ_2 extraction and errors

For small values of α, τ_2 diverges, such that very long simulations are required in order to evaluate τ_2. In order to simplify matters, we consider a linear expansion of Eq 7 of the main text in the region where $\tau_1 \ll t \ll \tau_2$:

$$M_2 = c_1 + (c_2 - c_1)(1 - e^{-t/\tau_2}) \approx c_1 + \frac{c_2 - c_1}{\tau_2} t. \tag{S1.4}$$

A linear fit to $M_2(t)$ data in the $\tau_1 \ll t \ll \tau_2$ can then be made, with intercept c_1 and slope $s = (c_2 - c_1)/\tau_2$. This allows τ_2 to be calculated, since c_2 is known from Eq 6 of the main text.

An example is shown in Fig G1a. Here, a set of 10 realizations of the simulation were performed. The plot contains the 10 different $M_2(t)$ curves overlayed one top of each other, with a linear fit (red line) to the combined data for all 10 curves. Fig G1b shows the distribution of residuals. The residuals are skewed, such that large increases in M_2 are more likely than large decreases.

Propagation of errors shows that $\Delta \ln[N/\tau_2] \approx \Delta s/s$, assuming that $\Delta s \gg \Delta c_1$ (justified below). This means that as α is decreased and s approaches 0, it eventually becomes impossible to obtain a meaningful fitted value of τ_2. Following this reasoning, we include points on the plot of $\ln[N/\tau_2]$ vs. α shown in Fig 5a of the main text for values of α for which $\Delta s/s < 1$.

Since $M_2(t)$ has a skewed (non-Normal) distribution of residuals and contains time-correlations, it is difficult to obtain a meaningful value of Δs from a regression. We therefore estimate Δs using uncorrelated, normally-distributed synthetic data with a width that is representative of the fluctuations in $M_2(t)$. This is done by generating random data from a Normal distribution with width equal to the average (over all time points) of the standard deviation taken, at each time t, across the 10 realizations of the simulation. An example of this synthetic data is shown in Fig G1b (black curve). A least squares fit provides a correct determination of the error on the slope and intercept of this synthetic data, which we use as estimates for Δs and Δc_1. In this way, we see that $\Delta s \gg \Delta c_1$.

This procedure provides an estimate of the (small α) limit beyond which points cannot be added to the plot of Fig 5a of the main text (obtaining further points would require longer simulation times that are beyond the scope of this study). The procedure does not provide a reasonable estimate of error bars for Fig 5a of the main text. However, $\Delta \ln[N/\tau_2]$ is expected to increase with decreasing α (since s decreases, while Δs remains approximately constant), and this is reflected in increased fluctuations of $\ln[N/\tau_2]$ about the straight black lines in Fig 5a of the main text as α decreases.

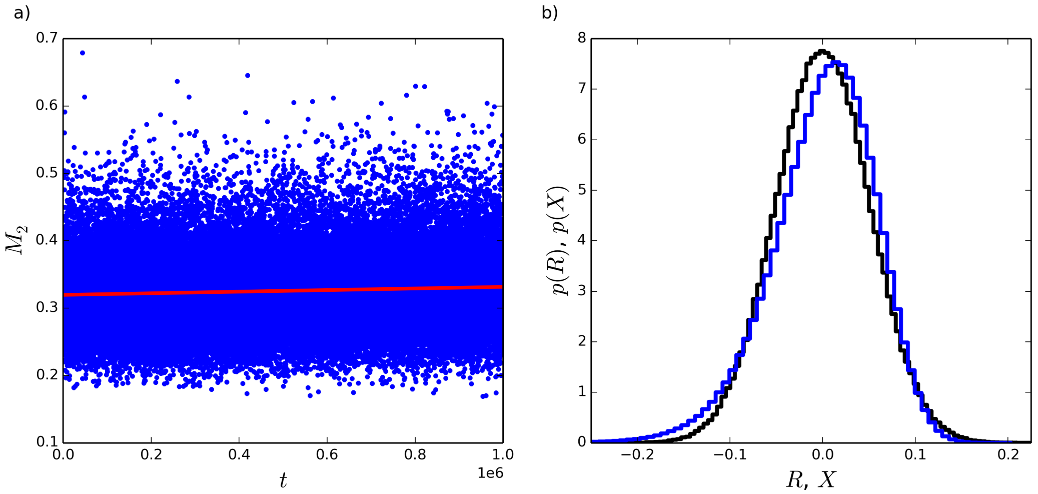

Fig G1 – (a) 10 overlaid realizations of $M_2(t)$ for $\delta = 0.2$, $\alpha = 0.2$, $N = 100$, $n_r = 10$, and linear fit (red line). (b) Distribution of residuals $R = y_{fit} - M_2$ (blue curve) and a Normal distribution with width equal to $\langle\sigma(M_2(t))\rangle$ of a random variable X (black curve).

H Equi-σ distributions

Essentially identical distributions can be produced (within a particular simulation time) for different combinations of the parameters δ and α. This is shown in Fig H1, which plots status distributions generated for three different points in $\delta - \alpha$ parameter-space. The inset to Fig H1 shows the distributions with a logarithmic scale on the y-axis, in order to allow closer inspection of the tails of the distributions. To compare the shapes of the distributions in Fig H1, in addition to visual inspection of the plotted curves, we use the time-and-realization-averaged standard deviation, $\langle\sigma\rangle$ of the status distributions. This is defined as follows: first, we average the standard deviation of the status distributions over 500 realizations of the simulation, giving us $\sigma(t)$. Then, we average $\sigma(t)$ over a range of time ($500 \leq t \leq 1000$) during which $\sigma(t)$ is approximately constant corresponding to the long-lived state. This gives us $\langle\sigma\rangle$. Repeating this procedure for the skewness of the status distributions gives us $\langle\mu_3/\sigma^3\rangle$, which we also use to compare the shapes of the distributions in Fig H1.

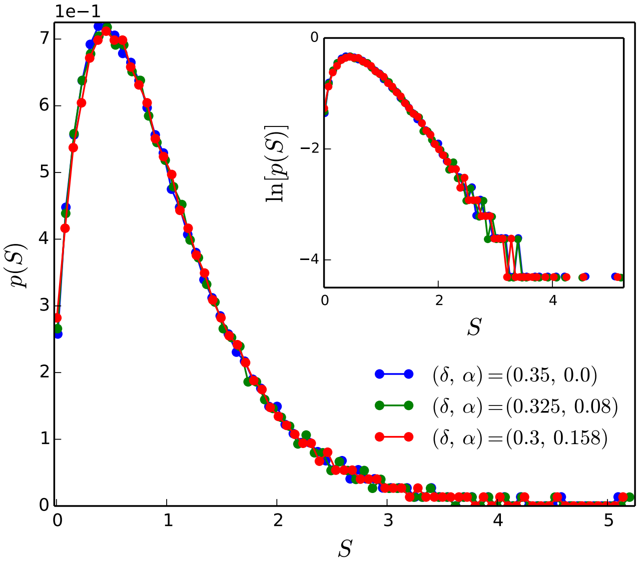

Fig H1 – Equi-σ status distributions, obtained at $\tau_{obs} = 1000$, for different pairs of (δ, α) values. Blue curve: $\langle\sigma\rangle = 0.733 \pm 0.001$; green curve: $\langle\sigma\rangle = 0.732 \pm 0.001$; red curve: $\langle\sigma\rangle = 0.732 \pm 0.001$. $\langle\mu_3/\sigma^3\rangle = 1.46 \pm 0.01$ for all three distributions.

The value of $\langle\sigma\rangle$ for the three distributions is the same, within error (caption of Fig H1). The error value used here is the standard deviation of $\sigma(t)$ taken over the range of time $500 \leq t \leq 1000$. Likewise, $\langle\mu_3/\sigma^3\rangle$ is the same, within error, for the three different distributions. There is, however, one subtle difference between the distributions that can be seen in Fig H1: the value of $p(S)$ at $S = 0$ becomes systematically larger going from smaller (larger) to larger (smaller) α (δ). Except for this caveat, Fig H1 shows the existence of sets of (δ, α) points producing essentially identical distributions.

I Determining the location of the transition between regions II and III in Fig 6 of the main text

I1 Using a criterion based on the slope of $M_2(t)$

The location of this transition between regions II and III of Fig 6 of the main text depends on the time, τ_{obs}, over which the system is observed (simulated). A simple criterion equates the onset of runaway with the appearance of a positive slope in the long-time portion of $M_2(t)$. Whether such a slope is observed in $M_2(t)$ depends on the size of the fluctuations in the simulation data. We ran $n_r = 25$ realizations of the simulation for system size $N = 1000$. In order to determine if the system is in the long-lived state for a particular value of α, we first fit a straight line to $M_2(t)$ over a range $[t_0,\, \tau_{obs}]$, where $t_0 \gg \tau_1$ such that $M(t_0)$ is on the $M_2(t)$ plateau that follows the initial transient evolution away from the initial (egalitarian) distribution. The transition is considered to have occurred at the value of α for which the long-time part of the $M_2(t)$ curve increases by 2.5% over the observation (simulation) time τ_{obs}. For the choice of N and n_r used, this amount of increase corresponds to approximately 5 times the standard deviation of the residual $R(t) = \hat{M}_2(t) - M_2(t)$, where $\hat{M}_2(t)$ is the linear fit, and $M_2(t)$ is averaged over the $n_r = 25$ realizations. Therefore, the distribution is considered to be long-lived over the interval $[t_0,\, \tau_{obs}]$ as long as $m(\tau_{obs} - t_0) < 0.025b$, where m is the slope and b the intercept of the linear fit.

I2 Using the Arrhenius relation between τ_2 and α

The criterion (see above, section I1) of a 2.5% increase in $M_2(t)$, used to determine the location of the transition between the long-lived state and runaway, can be written in terms of Eq 7 of the main text as:

$$M_2(\tau_{obs}) = 1.025c_1 \approx c_1 + (c_2 - c_1)(1 - e^{-\tau_{obs}/\tau_2}), \tag{S1.5}$$

where the approximation comes from the assumption that $\tau_{obs} \gg \tau_1$. Rearranging Eq. S1.5 in terms of τ_{obs} gives the following relationship:

$$\tau_{obs} \approx -\tau_2 \ln\left(1 - \frac{0.025c_1}{N - 1 - c_1}\right). \tag{S1.6}$$

Now, we fix τ_{obs} and adjust α until the relationship in Eq. S1.6 is satisfied. Inserting the relationships $\alpha_b = 0.53\delta^{-1.21}$ (Fig 5b of the main text) and $Nf_0 = \delta^{1.3}$ (lower inset of Fig 5b of the main text) into Eq 8 of the main text and rearranging, we find:

$$\begin{aligned} \alpha_t &\approx \frac{\alpha_b}{\ln[\tau_2(\alpha_t) f_0]} \\ &\approx \frac{0.53\delta^{-1.21}}{\ln\left[\frac{-\tau_{obs}\delta^{1.3}}{N\ln\left(1-\frac{0.025c_1}{N-1-c_1}\right)}\right]}, \end{aligned} \tag{S1.7}$$

where α_t is the location of the transition (as defined by the criterion stated above) and $\tau_2(\alpha_t)$ is the value of τ_2 when $\alpha = \alpha_t$.

A factor of c_1 appears twice in Eq. S1.7. While c_1 is in fact a function of δ and α, it changes slowly with α (Fig 4d of the main text). Therefore, we set c_1 to be equal to its value when $\alpha = 0$, such that $c_1 = \delta/(1-\delta)$ (see Eq 3 of the main text). With these assumptions in place, Eq. S1.7 produces the solid black ($\tau_{obs} = 10^4$) and red ($\tau_{obs} = 10^3$) curves in Fig 6 of the main text.

I3 N-dependence of α_t

The N-dependence of α_t is contained within the denominator of the argument of the logarithm in the denominator of Eq. S1.7. Fig I1 shows a plot of this part of Eq. S1.7 as a function of N. The plot becomes effectively constant for large N, showing that the N-dependence of α_t vanishes for increasing N such that the location of the transition between the long-lived state and runaway effectively does not depend on system size.

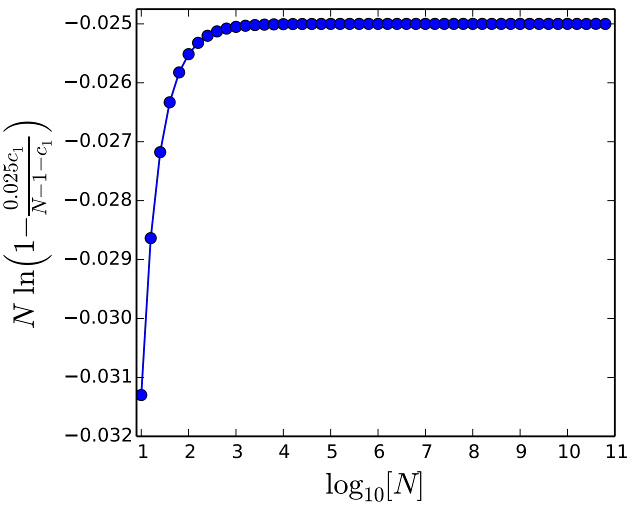

Fig I1 – The part of Eq. S1.7 containing a dependence on N plotted vs. powers of N, and becomes effectively constant at large N.

I4 Alternative location of the transition between regions II and III according to Arrhenius relation

In some physical systems governed by an Arrhenius relation, the location of the operational transition between two regimes is taken by the researcher to be at the value of the control parameter (usually temperature, in physical systems) for which the transition rate τ is equal to the measurement time τ_m. This is the case in the blocking transition of superparamagnetism, for example [1].

We could use this reasoning to locate the transition between the long-lived state and runaway in our model at a value of $\alpha = \alpha_t^*$ for which $\tau_2 = \tau_{obs}$. Re-arranging Eq. 8 of the main text and substituting in the relationships for α_b and Nf_0, we find:

$$\begin{aligned} \alpha_t^* &= \frac{\alpha_b}{\ln[\tau_{obs} f_0]} \\ &= \frac{0.53\delta^{-1.21}}{\ln\left[\frac{1.03\delta^{1.3}\tau_{obs}}{N}\right]}. \end{aligned} \tag{S1.8}$$

In a physical experiment, the criterion for setting the location of the operational transition is primarily determined by the measurement type. For some measurement types, it may be appropriate or natural to set the location of the transition at a value of the control parameter analogous to α_t^*. In our system, this would correspond to identifying the transition between the long-lived state and runaway at a value of α for which $M_2 = M_2(t = \tau_2) \approx c_2(1 - 1/e)$. This represents a significant evolution of the system toward the totalitarian end-state, which is not suitable for our purpose. Rather, our goal is to determine the value of α for which, on the scale of the observation time considered, the long-lived state plateau value of M_2 is lost and a noticeable increase in $M_2(t)$ is observed. We therefore place the location of the transition at α_t.

J Additional details about evolution of extended model distributions

The figures in this section are included to show how the status distributions produced by the extended model (presented in section 2.1 of the main text) evolve in time. In the upper left panel of each figure, the evolution of $M_2(t)$ on a log-log scale is shown. The status distributions are shown at various points in time (indicated within the plots) in the remaining panels. For each figure, the simulation had system size $N = 1000$, and the results are averaged over $n_r = 5$ realizations of the simulation. The simulation parameters $\delta = 0.2$, $\eta = 1.0$, and $\epsilon = 0.1$ are fixed for all figures, and the parameter α is varied.

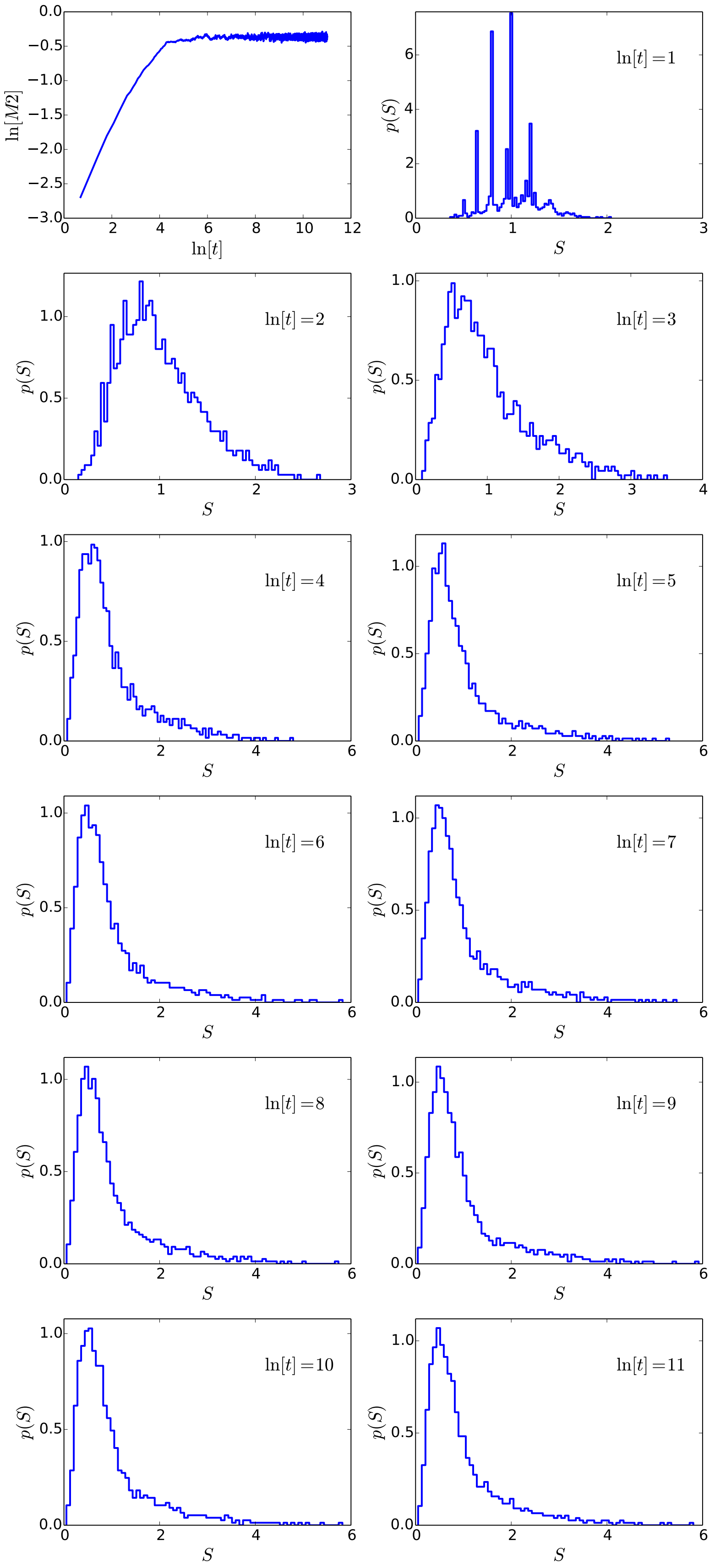

Fig J1 – Time evolution of distributions for $\delta = 0.2$, $\alpha = 0.0$, $\eta = 1.0$, $\epsilon = 0.1$.

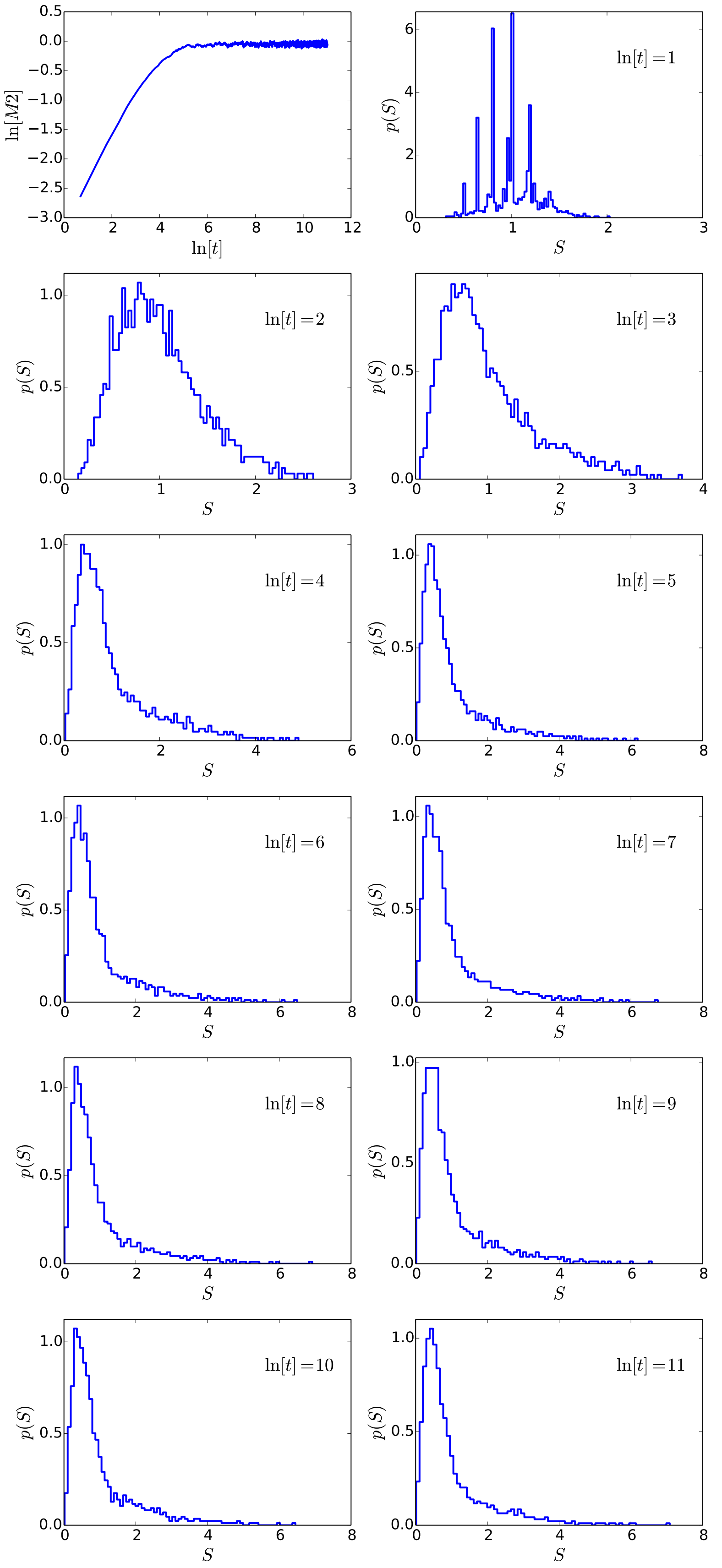

Fig J2 – Time evolution of distributions for $\delta = 0.2$, $\alpha = 0.2$, $\eta = 1.0$, $\epsilon = 0.1$.

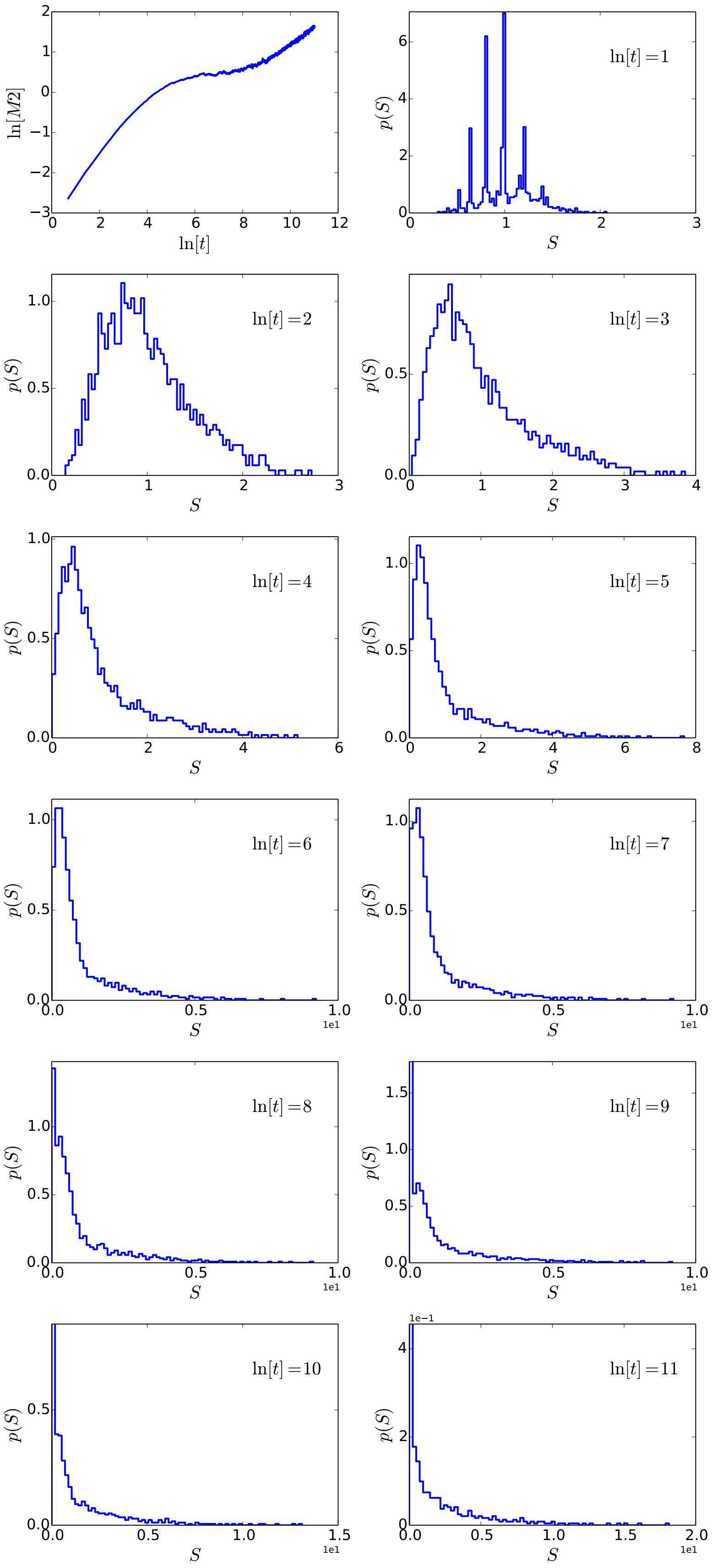

Fig J3 – Time evolution of distributions for $\delta = 0.2$, $\alpha = 0.4$, $\eta = 1.0$, $\epsilon = 0.1$.

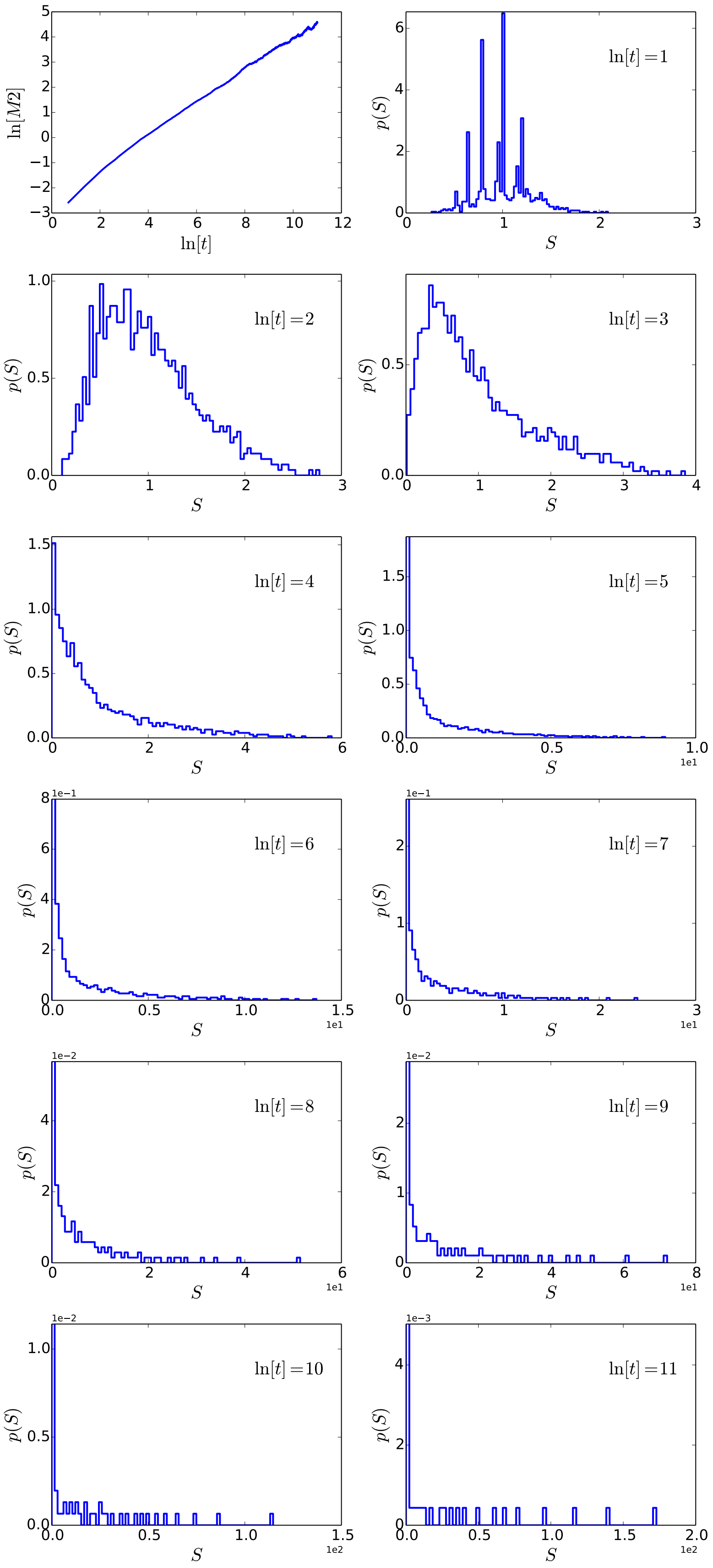

Fig J4 – Time evolution of distributions for $\delta = 0.2$, $\alpha = 0.75$, $\eta = 1.0$, $\epsilon = 0.1$.

References

[1] Knobel M, Nunes WC, Socolovsky LM, De Biasi E, Vargas JM, Denardin JC. Superparamagnetism and Other Magnetic Features in Granular Materials: A Review on Ideal and Real Systems. Journal of Nanoscience and Nanotechnology. 2008;8:2836–2857.
