# Supplementary material for: Self-organization and time-stability of social hierarchies
Source: PLoS One. 2019 Jan 29;14(1):e0211403. doi: 10.1371/journal.pone.0211403 (PMC6350989; doi:10.1371/journal.pone.0211403)
Supplement: S2 Appendix — (PDF) [file pone.0211403.s002.pdf]

S2 Appendix

Joseph Hickey and Jörn Davidsen

A Dependence of interaction probabilities on rank for mountain goats in study of Côté (2000)

In section 4.1 of the main text, we make use of data from the study of Côté [37] on agonistic interactions in female mountain goats collected during four consecutive summers. To compare results from our simulations with the mountain goat data across multiple years, we consider only those individual's in Côté's datasets that are present in all four years. This is a subset of the full dataset, since some mountain goats enter the society (e.g. by ageing to the age of three years old) and leave the society (e.g. by dying) from one year to the next.

To examine whether the mountain goats in our subset taken from Côté's data had a tendency to interact more frequently with those close in rank versus those far away in rank, we used the same approach as was used in Fig 6 of Ref. [37]. That is, for each individual, i, we calculated the percentage of i's interactions that involved one of the 10 individuals closest in rank to i, and the percentage of i's interactions that involved one of the 10 individuals furthest in rank from i. For the $N = 26$ individuals present in all 4 years of Côté's study, there was no bias, on average, toward interacting with individuals close in rank or far in rank. Results are presented in the following table.

Table A – Tendency of individuals to interact with other individuals close or far in rank in the $N = 26$ subset from Côté's mountain goat data. Each entry in columns 2-4 shows the percentage (averaged over the $N = 26$ individuals) of an individual's interactions that were against (column 2) one of the 10 closest ranked other individuals; (column 3) one of the 10 furthest ranked other individuals; or (column 4) an individual not belonging to the group of 10 closest to or 10 furthest in rank. Each error value shows the standard deviation of the percentage of interactions.

Year	10 closest	10 furthest	Other
1994	0.39 ± 0.09	0.4 ± 0.1	0.2 ± 0.1
1995	0.4 ± 0.2	0.4 ± 0.1	0.2 ± 0.1
1996	0.4 ± 0.1	0.4 ± 0.1	0.2 ± 0.1
1997	0.4 ± 0.1	0.4 ± 0.2	0.19 ± 0.09

Similar results are obtained when considering the 5 closest and 5 furthest individuals in rank and the 15 closest and 15 furthest individuals in rank.

B Measurable quantities that may serve as proxies for status in non-human animals

Table B – Measurable quantities that are relevant to the estimation of status proxies

Agonistic behaviour (fights)	• Agonistic interactions (fights) between pairs of individuals are observed in many studies and used to assign a rank to individuals in a dominance hierarchy. These can include physical aggressions, physical and non-physical (e.g. vocal, eye-contact) intimidations, and subordinations [1–4].<br>• Typically, an interaction matrix is constructed, with one row and column for each observed individual, where the entries are the numbers of times that individual i has defeated individual j in a fight [5–7].<br>• A ranking of the individuals in the study can be obtained by re-arranging the rows of the interaction matrix to satisfy a criterion (e.g. minimizing the sum of entries below the diagonal) [8] or by calculating a score from the interaction matrix (e.g. Davids Score) [9].<br>• Other studies make use of the sequence of interactions to rank individuals using a score that evolves each time an individual has an interaction. With these methods, the rank-ordering of the individuals can change as more interactions occur [5–7, 10].
Affiliative behaviours (grooming in primates)	• The grooming time that an individual receives correlates with hierarchical position (grooming is directed up the hierarchy) [11, 12], and may be given as a service in exchange for support in agonistic interactions or tolerance [13, 14]. However, individuals have limited grooming-time to give, and compete to groom higher-ranking individuals [15]. Therefore, grooming-time received by a high-status individual may underestimate her status.
Physical characteristics	• Whereas animals living in small groups may be able to assess the fighting abilities of one another by remembering the history of past interactions, when these same animals live in large groups (e.g. of $N \approx 100$ [16]), in which the role of individual recognition is reduced and where it is unlikely that every individual has interacted with every other individual, they may instead use physical characteristics (so-called "status signals") to communicate and assess fighting ability.<br>• In some cases, the status signal can change on a short time-scale, such as following a promotion or demotion in rank, such that it may signal the current fighting ability of the individual to others (e.g. the intensity of red colour in geladas and mandrills).<br>• Examples: Black facial marks in wasps [17, 18]; comb size in hens [16]; dark plumage throat-patch size in male sparrows [19]; red chest-patch colour in male geladas [20]; red face colour in male mandrills [21].

Biochemical concentrations (blood and saliva chemistry)	• Hormone and neurotransmitter concentrations are related to position in the dominance hierarchy and the dominance behaviour of the individual [1, 22, 23]. However, the relationship between biochemical concentrations and hierarchical rank is complicated in that it depends on social context. For example:<br>• High ranking individuals may have elevated levels of glucocorticoid (stress hormone), but for different reasons than low-ranking individuals with the same elevated level of glucocorticoid [24, 25].<br>• Testosterone concentration correlates with rank in the dominance hierarchy (higher-ranking individuals have higher concentrations of testosterone), but only in periods of societal instability [21, 22].<br>• Serotonin concentration correlates with rank, but is very sensitive to the presence of subordinates [26].
Body size (such as weight or length)	• Correlated with dominance rank and ability to win agonistic encounters in social insects, including ants, bees, and wasps [3, 27], crustaceans [28], fish [29, 30], and reptiles [31], although there are confounding factors such as age [32] and past fighting experience [33, 34].<br>• Less strongly related to dominance rank in more "complex" animals such as mammals [35–37].
Age	• Dominance rank is correlated with age in many species [32, 38, 39], although confounding factors include past fighting experience and body size [32].<br>• In many primates, the alpha eventually loses his/her position to a younger challenger [40–43], such that a simple linear relationship between age and dominance hierarchy rank is not accurate.
Preferential access to food	• Access to high quality food is correlated with dominance rank in many species, for example: salmon [44], caribou [45], deer [46], goats [47], macaques [48], chimpanzees [49], and baboons [50].<br>• Dominance not correlated with access to food in carrion crows [51].
Mating opportunities and reproduction	• Access to reproductive opportunities generally correlates with position in the dominance hierarchy [52, 53], however the relationship can be non-linear. For example, high ranking female baboons have been found to have more miscarriages and a higher rate of infertility [54], beta-male chimpanzees in some groups have much fewer copulations than lower-ranking males [55], and low-ranking male monkeys with affiliative relationships with females can have greater reproductive success than expected given their rank [1].

C Sensitivity of fit to USA income data to change in parameter ϵ

Fig 10b of the main text shows a fit of the extended model to the USA household income distribution. The parameter η is set such that $\eta\bar{S} = S_B$, where S_B is the "break point" in the data. The parameter ϵ in Fig 10b of the main text was chosen to be equal to 0.08 in order to obtain a good fit to the income data. Two figures are included below to show how decreasing (Fig C1) or increasing (Fig C2) the value of ϵ affects the fit to the proxy data.

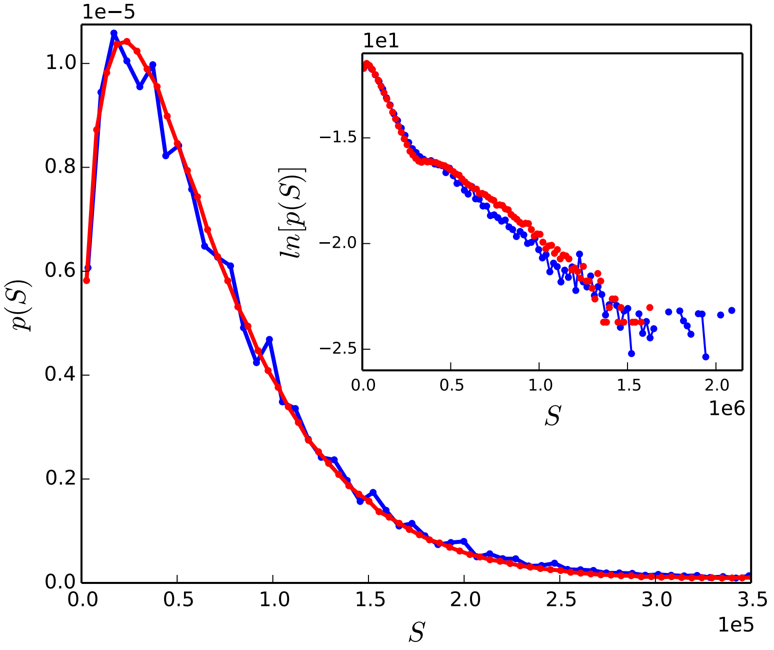

Fig C1 – Fit of extended model ($\eta = 3.5$, $\epsilon = 0.06$) to 2015 USA household income data.

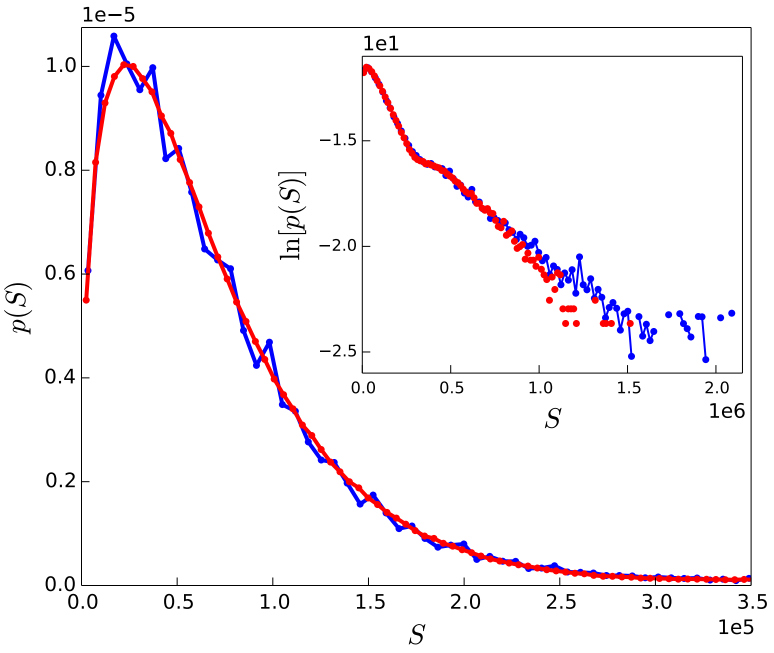

Fig C2 – Fit of extended model ($\eta = 3.5$, $\epsilon = 0.1$) to 2015 USA household income data.

D High-income tails of USA household income distributions

In Fig 11 of the main text, fits of exponential and power-law distributions to the high-income tail of the 2000 and 2015 USA household income distributions were shown. Normalized with respect to the boundaries S_l and S_h, the probability density function, $p(S)$, of the power-law distribution is:

$$p(S) = \frac{S_l^{1-\gamma} - S_h^{1-\gamma}}{\gamma - 1} S^{-\gamma}, \tag{S2.1}$$

and that of the exponential distribution is:

$$p(S) = \frac{e^{(S_l - S)/T}}{T(1 - e^{(S_l - S_h)/T})}, \tag{S2.2}$$

where the parameters γ and T are determined by maximum likelihood estimation using the data within the range $[S_l, S_h]$ [56]. Two additional figures showing different $[S_l, S_h]$ ranges are included below. As for Fig 11 of the main text, the black dashed line shows the power-law fit and the solid red line shows the exponential fit. The 2015 curves have been shifted down in the plots for better visualization.

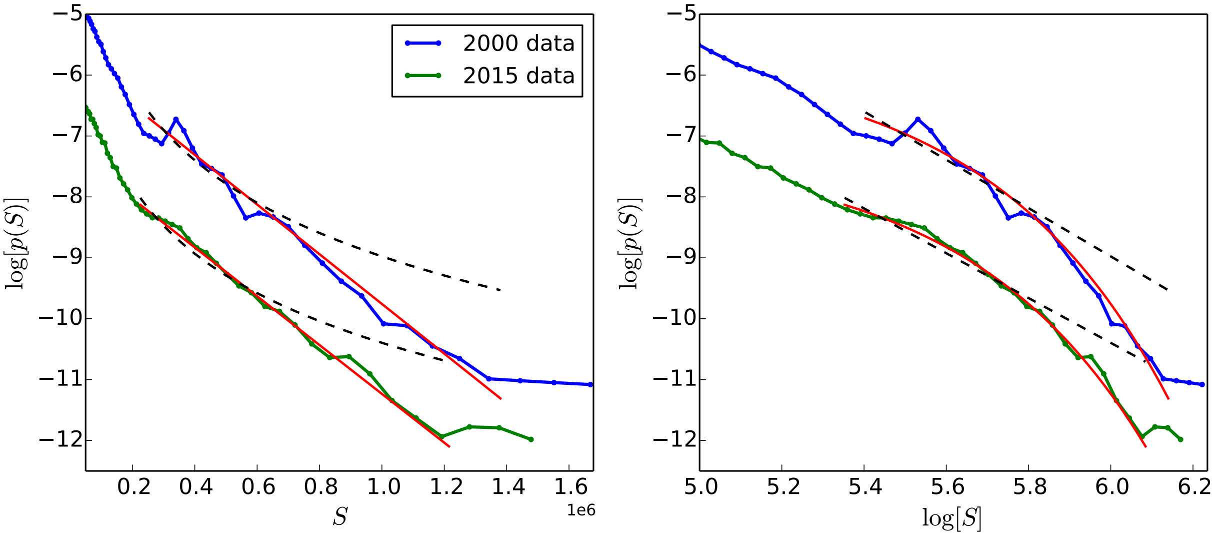

Fig D1 – Power-law (dashed black line) and exponential (solid red line) distributions with S_l and S_h chosen to correspond to the high-income tail, excluding the highest-income points. For the fits to the 2015 data shown in this figure, the choice of S_h resulted in the exclusion of the 15 largest data points. For the fits to the 2000 data shown in this figure, the choice of S_h resulted in the exclusion of the two largest data points. Exclusion of the highest-income data points is justified because high-income cutoffs have been artificially applied to the USA data by the governmental agency that provided it, for the purpose of protecting confidentiality. S represents USA household income data in 1999 USD.

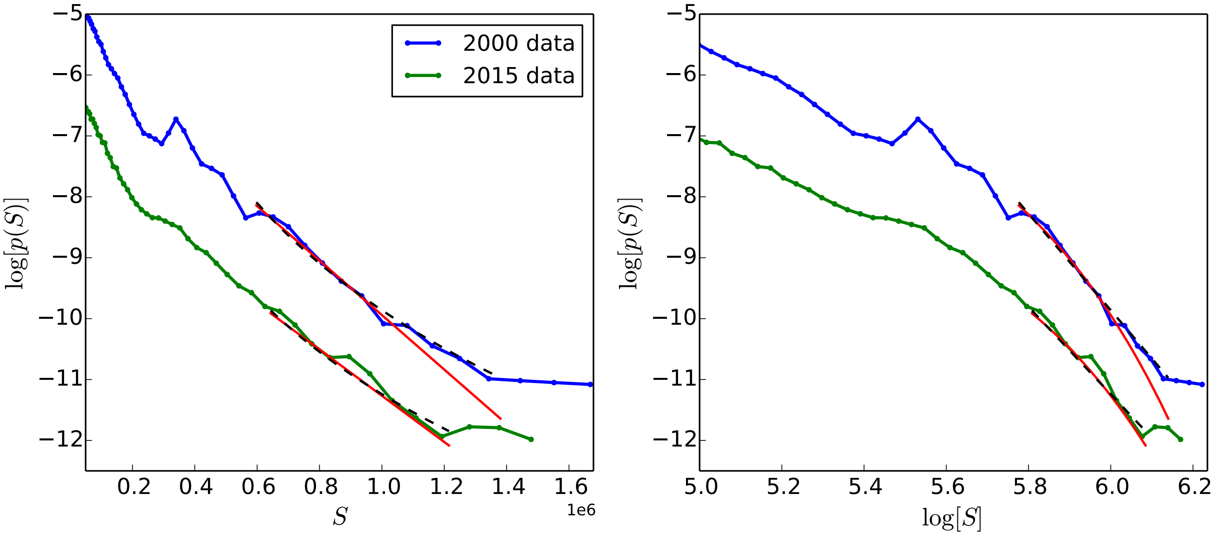

Fig D2 – Power-law (dashed black line) and exponential (solid red line) distributions with S_l and S_h chosen to correspond approximately to the latter part of the high-income tail. Graphical analysis shows that, at best, a power-law can only fit segments of the high-income tail. S represents USA household income data in 1999 USD.

D1 Kolmogorov-Smirnov (KS) test

A Kolmogorov-Smirnov (KS) test was used to evaluate whether the high-income tail of the USA household income distribution is consistent with a power-law distribution (Eq S2.1) or with an exponential distribution (Eq S2.2). The term "theoretical distribution" is used below to refer to the distribution (either power-law or exponential) that the real data is compared to in the statistical test.

The KS test relies on the "KS distance", which is the largest distance between the cumulative distribution function (CDF) of the theoretical distribution (with specified parameters) and the empirical cumulative distribution function, F_N, determined directly from the data as follows:

$$F_N(x) = \frac{1}{N}\sum_{i=1}^{N} I_{[-\infty,x]}(X_i), \tag{S2.3}$$

where $I_{[-\infty,x]}(X_i)$ is the indicator function, which is equal to 1 if $X_i \leq x$ and 0 otherwise, and N is the number of data points in the dataset. The 2015 USA household income dataset is a weighted dataset, such that every data point represents a particular number of people in the overall population. For this dataset, the weighted empirical cumulative distribution function [57] was used:

$$F_w(x) = \frac{1}{\sum_i w_i}\sum_{i=1}^{N} w_i I_{[-\infty,x]}(X_i), \tag{S2.4}$$

where w_i is the weight assigned to the i_{th} data point.

Since the parameters γ (power-law distribution) and T (exponential distribution) are unknown, they are estimated from the data using maximum likelihood estimation for the unweighted 2000 dataset and weighted maximum likelihood estimation for the 2015 dataset [58].

The KS distance, D_{data}, between the empirical CDF (F_N or F_w) and the CDF of the theoretical distribution is compared to a distribution of KS distances determined from synthetic datasets. To obtain the latter distribution of KS distances, the following steps are repeated many times: 1) a sample of synthetic data containing the same number of data points as the real data is drawn from the theoretical distribution; 2) the parameters of the theoretical distribution are re-estimated from the synthetic data, in order to avoid biases that arise if this re-estimation is not performed [59]; 3) the KS distance, D_{synth}, is determined from the empirical CDF of the synthetic data and the theoretical distribution with re-estimated parameters.

If the real data is consistent with the theoretical distribution, D_{data} should be smaller than a significant fraction of the set of $\{D_{synth}\}$. The fraction of $\{D_{synth}\}$ that is larger than D_{data} is the p-value given by the test. We consider a p-value greater than 0.1 to be evidence of compatibility with the theoretical distribution. In other words, with a p-value greater than 0.1, there is not enough evidence to reject the null hypothesis that the real data comes from the theoretical distribution.

A KS test showed that the high-income tail of the 2015 USA household income data is only compatible (p-value > 0.1) with an exponential distribution within particular ranges $[S_l, S_h]$ within the high-income tail, the largest of which is $\$700,000$ to $\$1,700,000$ ($\$492,800$ to $\$1,196,800$ in 1999 USD), and that the high-income tail is not compatible with a power-law for any of the ranges tested. The KS test for the 2000 USA household data also shows that the data is only compatible with an exponential distribution within particular ranges, the largest being from $\$800,000$ to the largest income value in the dataset ($\$1,668,400$), and that the high-income tail is not compatible with a power-law for any of the ranges tested.

E Alternative "extended" models that pre-suppose a two-class structure

E1 Addition to extended model to allow fights between high-status individuals

In the extended model presented in section 2.1 of the main text, individuals at the top-end of the status distribution are separated by large amounts of status, typically greater than the amount $\eta\bar{S}$. These high-status individuals are therefore prevented from fighting with each other by the first condition ($S1 - S2 > \eta\bar{S}$) of the extended model. This excludes many fights between high-status "dominant" individuals and higher-than-average status "challengers", whereas such dominant-challenger fights are important and common in real dominance hierarchies [24, 25, 60]. In order to allow high-status individuals to fight each other more frequently, we introduce a third condition to the extended model, such that the fight occurs if the statuses of both potential competitors are greater than the threshold amount $\eta\bar{S}$ (equivalently, if $S_2 > \eta\bar{S}$, since $S_1 >= S_2$). Under this modification to the extended model, the fight occurs if $S_1 - S_2 \leq \eta\bar{S}$ OR $S2 > \eta\bar{S}$ OR $r \leq \epsilon$, where r is a random number between 0 and 1. The additional condition $S2 > \eta\bar{S}$, pre-supposes a two-class structure *a priori*. The status distributions produced by this 3-condition extended model have large-S tails that decay exponentially over the full extent of the tail, unlike those produced by the 2-condition extended model presented in section 2.1 of the main text, which show a cutoff at very high values of S (see Fig 7d of the main text). The status distributions of the 3-condition model therefore show improved fits to the proxy data, as can be seen in Fig E1.

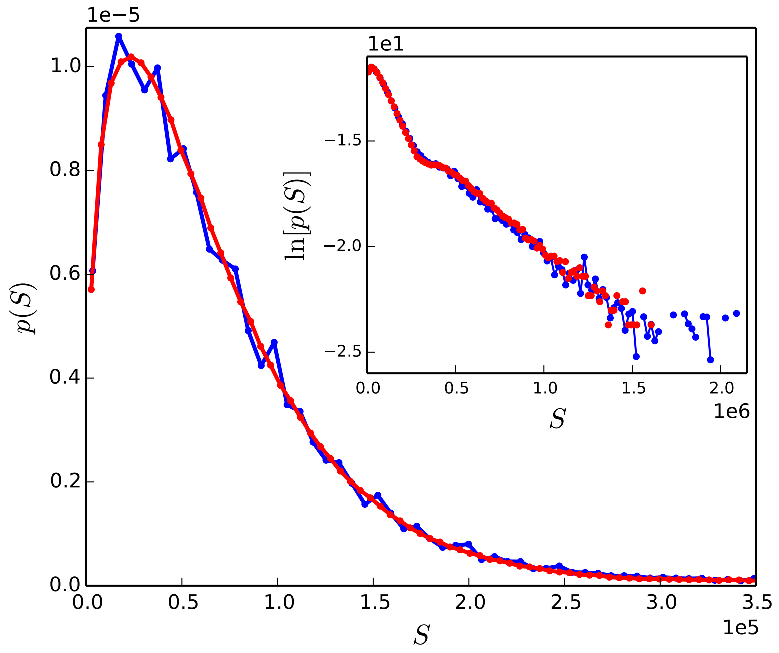

Fig E1 – Fit of 3-condition extended model status distribution (red curve) to the 2015 USA household income distribution (blue curve). Simulation parameters: $\delta = 0.4$, $\alpha = 0$, $\eta = 3.5$, $\epsilon = 0.08$.

E2 Combining two independently simulated status distributions

Here, we have used the original (two-parameter) model presented in section 2 of the main text and simulated two separate populations with different N and $\bar{S}$. The combination of the two simulated status distribution provides a good fit to the American household income distribution. This is shown in Fig E2, where the blue curve is the American household income distribution data for the year 2015, the red and

green curves represent the two independently-simulated status distributions, and the cyan curve shows the combination of the two simulated distributions. The simulation that produced the green curve contained $N_1 = 0.1N_{dat}$ individuals, where $N_{dat} = 1{,}226{,}728$ is the number of households in the dataset. For this first simulated society, $\bar{S}_1 = 225,000$. The simulation that produced the red curve contained $N_2 = 0.9N_{dat}$ individuals with $\bar{S}_2 = 64,445$. $\bar{S}_1$ and $\bar{S}_2$ were chosen so that the total status of the two simulated systems $\bar{S}_1 N_1 + \bar{S}_2 N_2$ was equal to the total household income reported in the dataset.

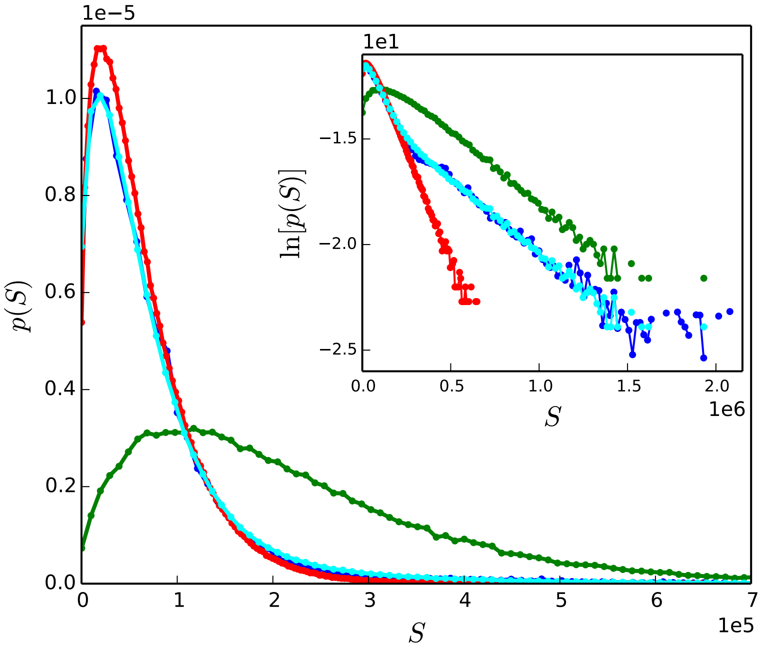

Fig E2 – Fit of simulated distribution to USA data. Cyan curve is the combination of red and green distributions. For red curve, $N = 0.9N_{dat}$, $\bar{S} = 64,445$, and $\delta = 0.4$; for green curve, $N = 0.1N_{dat}$, $\bar{S} = 225,000$, and $\delta = 0.35$. $\alpha = 0$ for both red and green curves.

As shown in Fig E2, the American household income data is well-represented by the combination of the status distributions of (i) a simulated system with a relatively small number of individuals having a relatively high average status, and (ii) a simulated system with a relatively large number of individuals having a relatively modest average status. This approach differs from those presented in sections 2.1 of the main text and section E1 above, by assuming that the society consists of two separate groups for which the members of each group engage in status-determining interactions amongst themselves, but for which there are no cross-group interactions. The extended model presented in section 2.1 of the main text makes no such assumptions.

References

[1] Sapolsky RM. The Influence of Social Hierarchy on Primate Health. Science. 2005;308(5722):648–652. doi:10.1126/science.1106477.

[2] Hsu Y, Earley RL, Wolf LL. Modulation of aggressive behaviour by fighting experience: mechanisms and contest outcomes. Biol Rev. 2018;81(2006):33–74. doi:10.1017/S146479310500686X.

[3] Hardy ICW, Goubault M, Batchelor TP. Hymenopteran contests and agonistic behaviour. In: Hardy ICW, Briffa M, editors. Anim. Contests. Cambridge University Press; 2013. p. 147–177.

[4] Shizuka D, Mcdonald DB. The network motif architecture of dominance hierarchies. J R Soc Interface. 2015;12:20150080. doi:10.1098/rsif.2015.0080.

[5] Albers PCH, De Vries H. Elo-rating as a tool in the sequential estimation of dominance strengths. Anim Behav. 2001;61:489–495. doi:10.1006/anbe.2000.1571.

[6] Neumann C, Duboscq J, Dubuc C, Ginting A, Irwan AM, Agil M, et al. Assessing dominance hierarchies: validation and advantages of progressive evaluation with Elo-rating. Anim Behav. 2011;82:911–921. doi:10.1016/j.anbehav.2011.07.016.

[7] Sánchez-Tójar A, Schroeder J, Farine DR. A practical guide for inferring reliable dominance hierarchies and estimating their uncertainty. J Anim Ecol. 2018;87:594–608. doi:10.1111/1365-2656.12776.

[8] De Vries H. Finding a dominance order most consitent with a linear hierarchy: a new procedure and review. Anim Behav. 1998;55:827–843. doi:10.1006/anbe.1997.0708.

[9] David HA. Ranking from unbalanced paired-comparison data. Biometrika. 1987;74(2):432–436.

[10] Newton-fisher NE. Modeling Social Dominance: Elo-Ratings, Prior History, and the Intensity of Aggression. Int J Primatol. 2017;38:427–447. doi:10.1007/s10764-017-9952-2.

[11] Tiddi B, Aureli F, Schino G. Grooming up the hierarchy: The exchange of grooming and rank-related benefits in a new world primate. PLoS One. 2012;7(5):3–8. doi:10.1371/journal.pone.0036641.

[12] Snyder-Mackler N, Kohn JN, Barreiro LB, Johnson ZP, Wilson ME, Tung J. Social status drives social relationships in groups of unrelated female rhesus macaques. Anim Behav. 2016;111:307–317. doi:10.1016/j.anbehav.2015.10.033.

[13] Schino G. Grooming and agonistic support: a meta-analysis of primate reciprocal altruism. Behav Ecol. 2007;18(1):115–120. doi:10.1093/beheco/arl045.

[14] Seyfarth RM. A model of social grooming among adult female monkeys. J Theor Biol. 1977;65(4):671–698. doi:10.1016/0022-5193(77)90015-7.

[15] Schino G. Grooming, competition and social rank among female primates: a meta-analysis. Anim Behav. 2001;62(2):265–271. doi:10.1006/anbe.2001.1750.

[16] D'Eath RB, Keeling LJ. Social discrimination and aggression by laying hens in large groups: from peck orders to social tolerance. Appl Anim Behav Sci. 2003;84(3):197–212. doi:10.1016/j.applanim.2003.08.010.

[17] Tibbetts EA, Lindsay R. Visual signals of status and rival assessment in Polistes dominulus paper wasps. Biol Lett. 2008;4(3):237–239. doi:10.1098/rsbl.2008.0048.

[18] Tibbetts EA, Shorter JR. How do fighting ability and nest value influence usurpation contests in Polistes wasps? Behav Ecol Sociobiol. 2009;63(9):1377–1385. doi:10.1007/s00265-009-0764-z.

[19] Møller AP. Variation in badge size in male house sparrows Passer domesticus: evidence for status signalling. Anim Behav. 1987;35(6):1637–1644. doi:10.1016/S0003-3472(87)80056-8.

[20] Bergman TJ, Ho L, Beehner JC. Chest color and social status in male geladas (Theropithecus gelada). Int J Primatol. 2009;30(6):791–806. doi:10.1007/s10764-009-9374-x.

[21] Setchell JM, Smith T, Wickings EJ, Knapp LA. Social correlates of testosterone and ornamentation in male mandrills. Horm Behav. 2008;54(3):365–372. doi:10.1016/j.yhbeh.2008.05.004.

[22] Eisenegger C, Haushofer J, Fehr E. The role of testosterone in social interaction. Trends Cogn Sci. 2011;15(6):263–271. doi:10.1016/j.tics.2011.04.008.

[23] Mccall C, Singer T. The animal and human neuroendocrinology of social cognition, motivation and behavior. Nat Neurosci. 2012;15(5):681–688. doi:10.1038/nn.3084.

[24] Gesquiere LR, Learn NH, Simao MCM, Onyango PO, Alberts SC, Altmann J. Life at the Top: Rank and Stress in Wild Male Baboons. Science. 2011;333(6040):357–360. doi:10.1126/science.1207120.

[25] Sapolsky RM. Sympathy for the CEO. Science. 2011;333(6040):293–294. doi:10.1126/science.1209620.

[26] Watanabe N, Yamamoto M. Neural mechanisms of social dominance. Front Neurosci. 2015;9(APR). doi:10.3389/fnins.2015.00154.

[27] Tibbetts EA, Dale J. A socially enforced signal of quality in a paper wasp. Nature. 2004;432:218–222. doi:10.1038/nature03004.1.

[28] Adams ES, Caldwell RL. Deceptive communication in asymmetric fights of the stomatopod crustacean Gonodactylus bredini. Anim Behav. 1990;39:706–716.

[29] Beaugrand JP, Payette D, Goulet C. Conflict Outcome in Male Green Swordtail Fish Dyads (Xiphophorus helleri): Interaction of Body Size, Prior Dominance/Subordination Experience, and Prior Residency. Behaviour. 1996;133(3):303–319.

[30] Buston P. Size and growth modification in clownfish. Nature. 2003;424:145–146.

[31] Schuett GW. Body size and agonistic experience affect dominance and mating success in male copperheads. Anim Behav. 1997;54:213–224. doi:10.1006/anbe.1996.0417.

[32] Hughes CR, Strassman JE. Age Is More Important than Size in Determining Dominance among Workers in the Primitively Eusocial Wasp, Polistes instabilis. 1Behaviour. 1988;107:1–14.

[33] Withee JR, Rehan SM. Cumulative effects of body size and social experience on aggressive behaviour in a subsocial bee. Behaviour. 2016;153(12):1365–1385. doi:10.1163/1568539X-00003382.

[34] Rutte C, Taborsky M, Brinkhof MWG. What sets the odds of winning and losing? Trends Ecol Evol. 2006;21(1):16–21. doi:10.1016/j.tree.2005.10.014.

[35] Pusey A, Williams J, Goodall J. The Influence of Dominance Rank on the Reproductive Success of Female Chimpanzees. Science (80-). 1997;277(5327):828–831. doi:10.1126/science.277.5327.828.

[36] Paoli T, Palagi E, Borgognini Tarli SM. Reevaluation of Dominance Hierarchy in Bonobos (Pan paniscus). Am J Phys Anthropol. 2006;130:116–122. doi:10.1002/ajpa.20345.

[37] Cote SD. Dominance hierarchies in female mountain goats: stability, aggressiveness, and determinants of rank. Behaviour. 2000;137:1541–1566.

[38] Higashi S, Ito F, Sugiura N, Ohkawara K. Workers' age regulates the linear dominance hierarchy in the queenless ponerine ant, Pachycondyla sublaevis (Hymenoptera: Formicidae). Anim Behav. 1994;47:179–184.

[39] Thouless CR, Guinness FE. Conflict between red deer hinds: the winner always wins. Anim Behav. 1986;34(4):1166–1171. doi:10.1016/S0003-3472(86)80176-2.

[40] Setchell JM, Smith T, Wickings EJ, Knapp LA. Stress, social behaviour, and secondary sexual traits in a male primate. Horm Behav. 2010;58(5):720–728. doi:10.1016/j.yhbeh.2010.07.004.

[41] Zhao D, Li B. Do deposed adult male Sichuan snub-nosed monkeys Rhinopithecus roxellana roam as solitary bachelors or continue to interact with former band members? Curr Zool. 2009;55(3):235–237.

[42] Uehara S, Hiraiwa-Hasegawa M, Hosaka K, Hamai M. The fate of defeated alpha male chimpanzees in relation to their social networks. Primates. 1994;35(1):49–55. doi:10.1007/BF02381485.

[43] Sapolsky RM. The physiology of dominance in stable versus unstable social hierarchies. In: Mason WA, Mendoza SP, editors. Primate Soc. Confl. Albany, NY: State University of New York Press; 1993. p. 171–204.

[44] Maclean A, Metcalfe NB. Social status, access to food, and compensatory growth in juvenile Atlantic salmon. J Fish Biol. 2001;58(5):1331–1346. doi:10.1006/jfbi.2000.1545.

[45] Barrette C, Vandal D. Social Rank, Dominance, Antler Size, and Access to Food in Snow-Bound Wild Woodland Caribou. Behaviour. 1986;97(1):118–146.

[46] Appleby MC. Social Rank and Food Access in Red Deer Stags. Behaviour1. 1980;74(3):294–309.

[47] Barroso FG, Alados CL, Boza J. Social hierarchy in the domestic goat: effect on food habits and production. Appl Anim Behav Sci. 2000;69(1):35–53. doi:10.1016/S0168-1591(00)00113-1.

[48] Sterck EHM, Steenbeek R. Female Dominance Relationships and Food Competition in the Sympatric Thomas Langur and Long-Tailed Macaque. Behaviour1. 1997;134(9):749–774.

[49] Wittig RM, Boesch C. Food Competition and Linear Dominance Hierarchy among Female Chimpanzees of the Taï National National Park. Int J Primatol. 2003;24(4):847–867. doi:10.1023/A:1024632923180.

[50] Marmot MG, Sapolsky RM. Of Baboons and Men: Social Circumstances, Biology, and the Social Gradient in Health. In: Weinstein M, Lane MA, editors. Soc. Hierarchy, Heal. Comp. Biodemography A Collect. Pap. Washington, DC: The National Academies Press; 2014.

[51] Richner H. Phenotypic correlates of dominance in carrion crows and their effects on access to food. Anim Behav. 1989;38:606–612.

[52] Cowlishaw G, Dunbar RIM. Dominance rank and mating success in male primates. Anim Behav. 1991;41:1045–1056.

[53] Ellis L. Dominance and Reproductive Success Among Nonhuman Animals: A Cross-Species Comparison. Ethol Sociobiol. 1995;16:257–333.

[54] Packer C, Collins DA, Sindimwo A, Goodall J. Reproductive constraints on aggressive competition in female baboons; 1995.

[55] Newton-Fisher NE. Hierarchy and social status in Budongo chimpanzees. Primates. 2004;45(2):81–87. doi:10.1007/s10329-003-0064-6.

[56] Baró J, Vives E. Analysis of power-law exponents by maximum-likelihood maps. Physical Review E. 2012;85(6):1–13. doi:10.1103/PhysRevE.85.066121.

[57] SAS Institute Inc. SAS/INSIGHT User's Guide. SAS Online Doc, Version 8. 2000. Available from: `http://www.okstate.edu/sas/`.

[58] Wang SX. Maximum Weighted Likelihood Estimation. Thesis. 2001;PhD Thesis:160.

[59] Capasso M, Alessi L, Barigozzi M, Fagiolo G. On approximating the distributions of goodness-of-fit test statistics based on the empirical distribution function: The case of unknown parameters. Advances in Complex Systems. 2009;12(2):157. doi:10.1142/S0219525909002131.

[60] Sapolsky RM. Cortisol concentrations and the social significance of rank instability among wild baboons. Psychoneuroendocrinology. 1992;17(6):701–709. doi:10.1016/0306-4530(92)90029-7.
